# Supplementary material for: Radical Pairing Interactions and Donor–Acceptor Interactions in Cyclobis(paraquat-p-phenylene) Inclusion Complexes
Source: Molecules. 2023 Feb 22;28(5):2057. doi: 10.3390/molecules28052057 (PMC10004262; doi:10.3390/molecules28052057)
Supplement: Supplementary file 1 [file molecules-28-02057-s001.zip › molecules-2169317-supplementary.pdf]

I. Supplementary figures and tables

Figure S1. Intermolecular BCPs found in the a) NDI-CBPQT^{4+} b) NDI-CBPQT$^{\bullet 3+}$ c) NDI-CBPQT$^{2(\bullet +)}$(T) d) NDI-CBPQT$^{\bullet +}$ e) NDI-CBPQT^{0} f) NDI-CBPQT$^{2(\bullet +)}$(S) complexes.

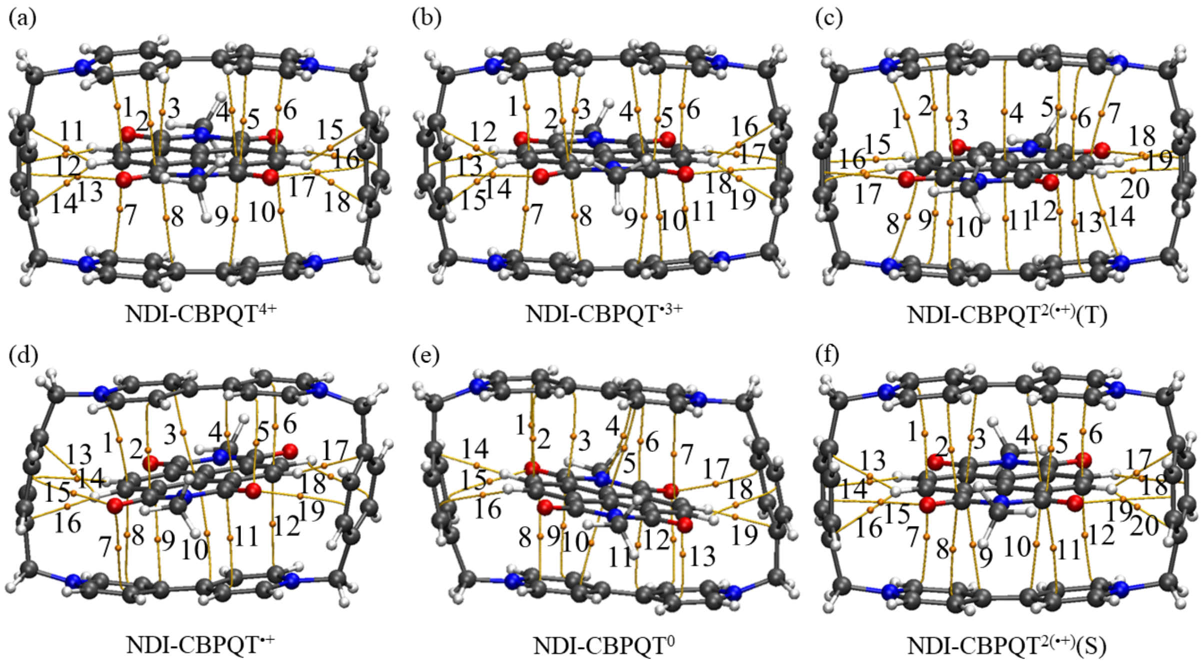

Figure S2. IGM plot iso-surfaces of NDI-CBPQT^{n+} (n=0-4) complexes. δg^{inter} = 0.005 a.u. All iso-surfaces are colored according to a BGR scheme over the range -0.03 < sign(λ_2)ρ < +0.05 a.u.

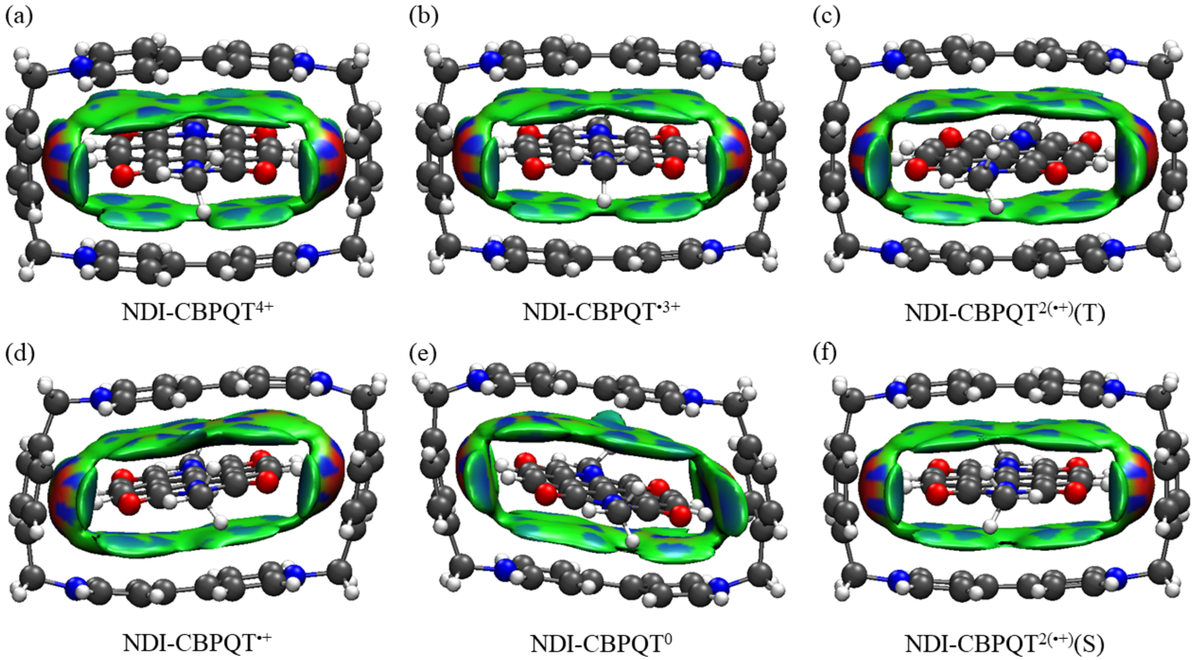

Figure S3. Intermolecular BCPs found in the a) TTF-CBPQT^{4+} b) TTF-CBPQT$^{\bullet 3+}$ c) TTF-CBPQT$^{2(\bullet +)}$(T) d) TTF-CBPQT$^{\bullet +}$ e) TTF-CBPQT^{0} f) TTF-CBPQT$^{2(\bullet +)}$(S) complexes.

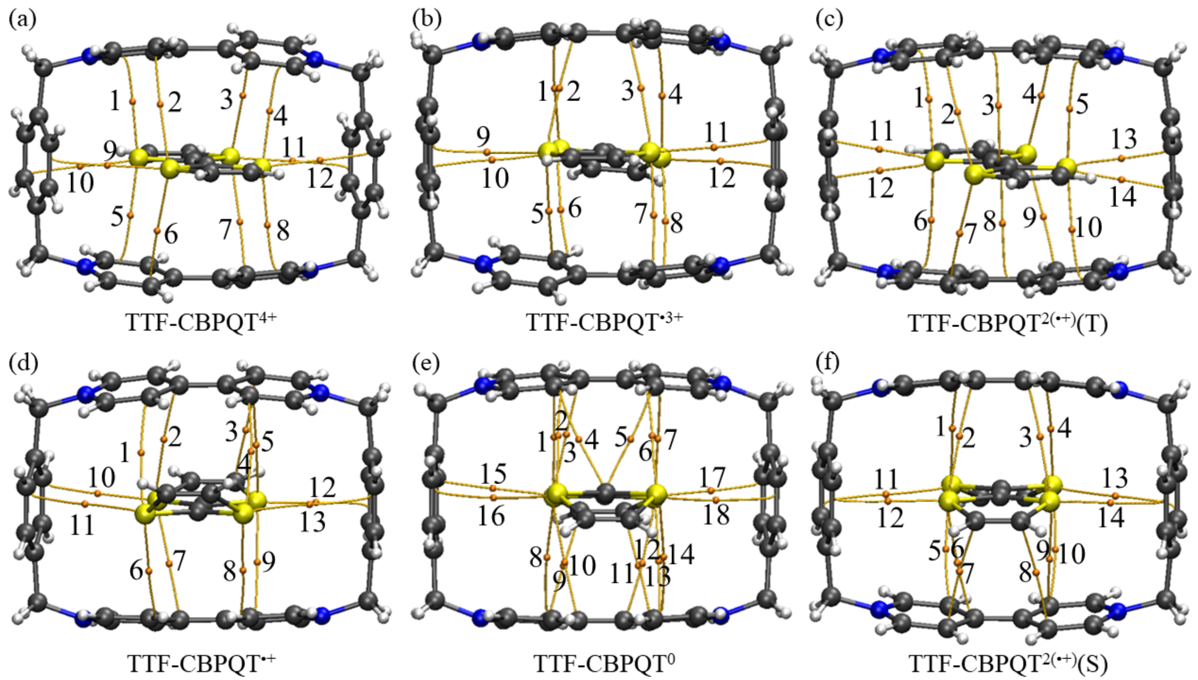

Figure S4. IGM plot iso-surfaces of TTF-CBPQT^{n+} (n=0-4) complexes. δg^{inter} = 0.005 a.u. All iso-surfaces are colored according to a BGR scheme over the range -0.03 < sign(λ_2)ρ < +0.05 a.u.

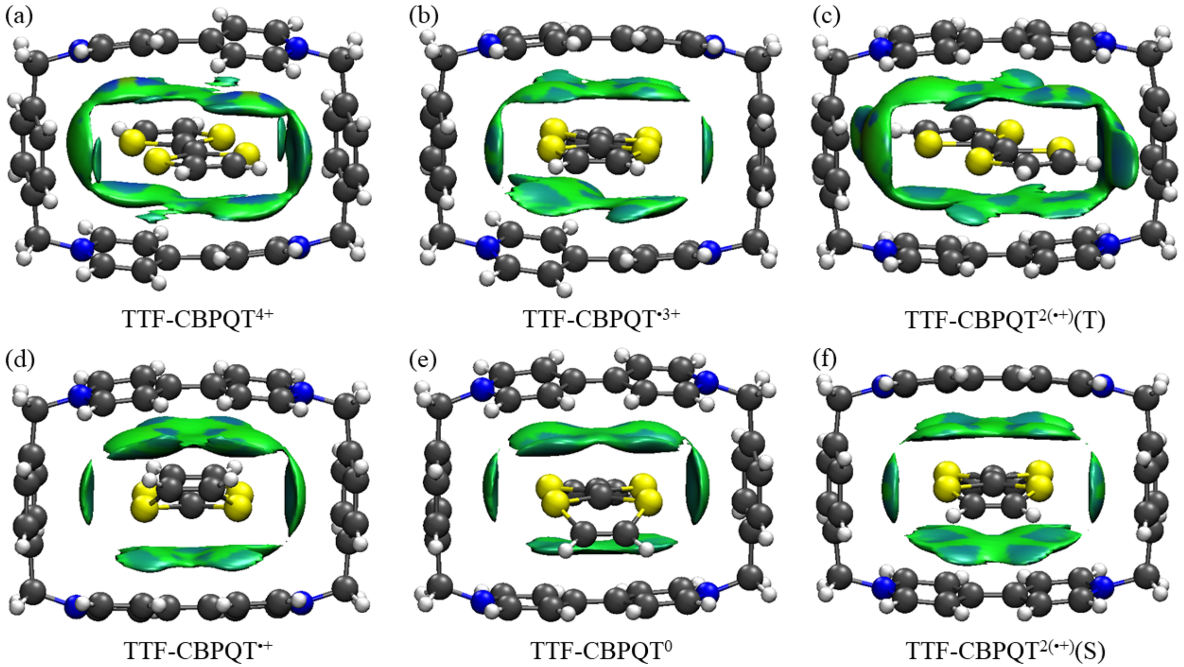

Figure S5. Intermolecular BCPs in a) BIPY$^{\bullet+}$-CBPQT^{4+} b) BIPY$^{\bullet+}$-CBPQT$^{\bullet3+}$ c) BIPY$^{\bullet+}$-CBPQT$^{2(\bullet+)}$(T) d) BIPY$^{\bullet+}$-CBPQT$^{\bullet+}$ e) BIPY$^{\bullet+}$-CBPQT^{0} f) BIPY$^{\bullet+}$-CBPQT$^{2(\bullet+)}$(S) complexes.

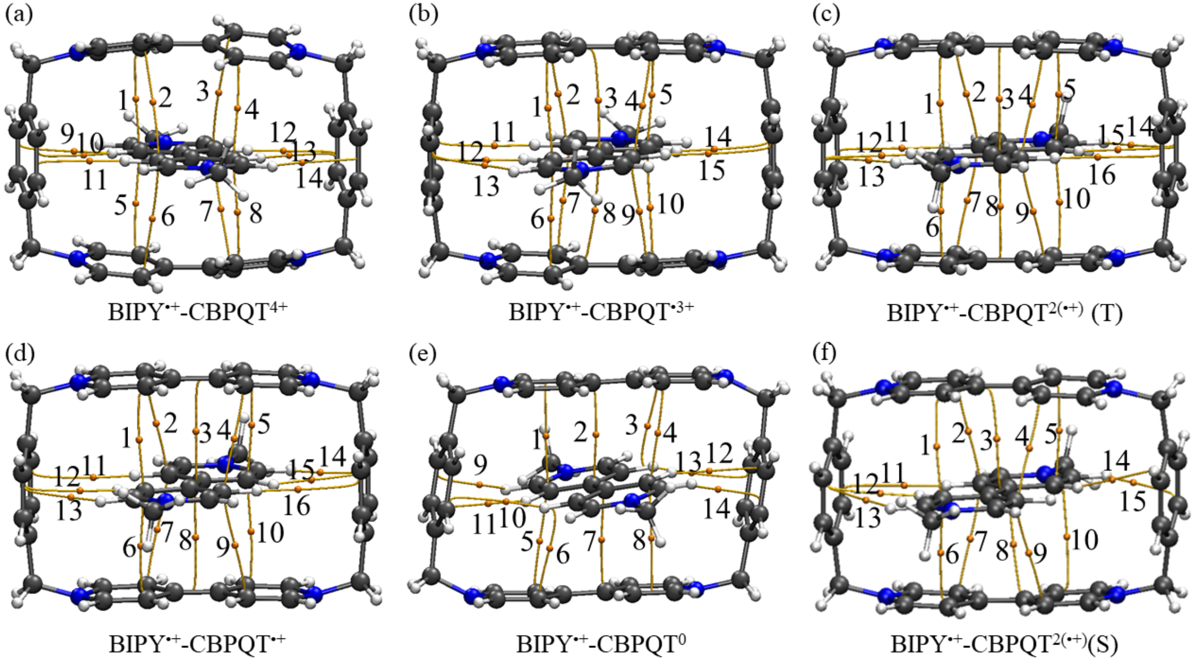

Figure S6. IGM plot iso-surfaces of $BIPY^{\bullet+}$-$CBPQT^{n+}$ (n=0-4) complexes. δg^{inter} = 0.005 a.u. All iso-surfaces are colored according to a BGR scheme over the range -0.03 < sign(λ_2)ρ < +0.05 a.u.

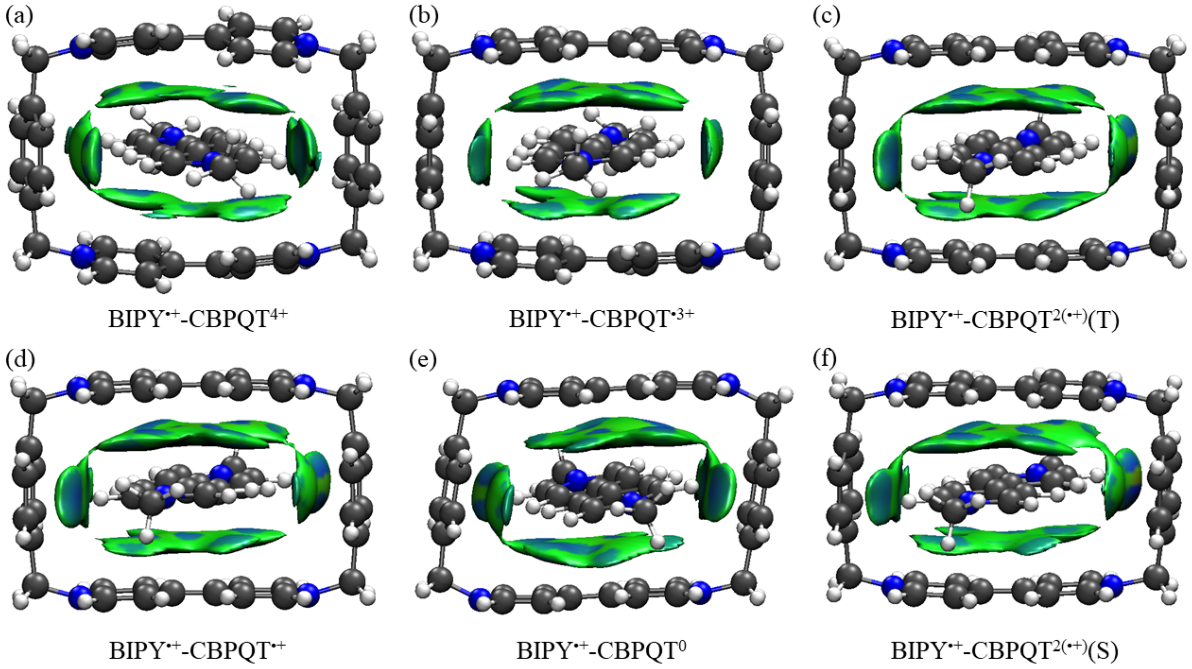

Figure S7. Intermolecular BCPs found in a) NDI$^{\bullet-}$-CBPQT^{4+} b) NDI$^{\bullet-}$-CBPQT$^{\bullet 3+}$ c) NDI$^{\bullet-}$-CBPQT$^{2(\bullet+)}$(T) d) NDI$^{\bullet-}$-CBPQT$^{\bullet+}$ e) NDI$^{\bullet-}$-CBPQT^{0} f) NDI$^{\bullet-}$-CBPQT$^{2(\bullet+)}$(S) complexes.

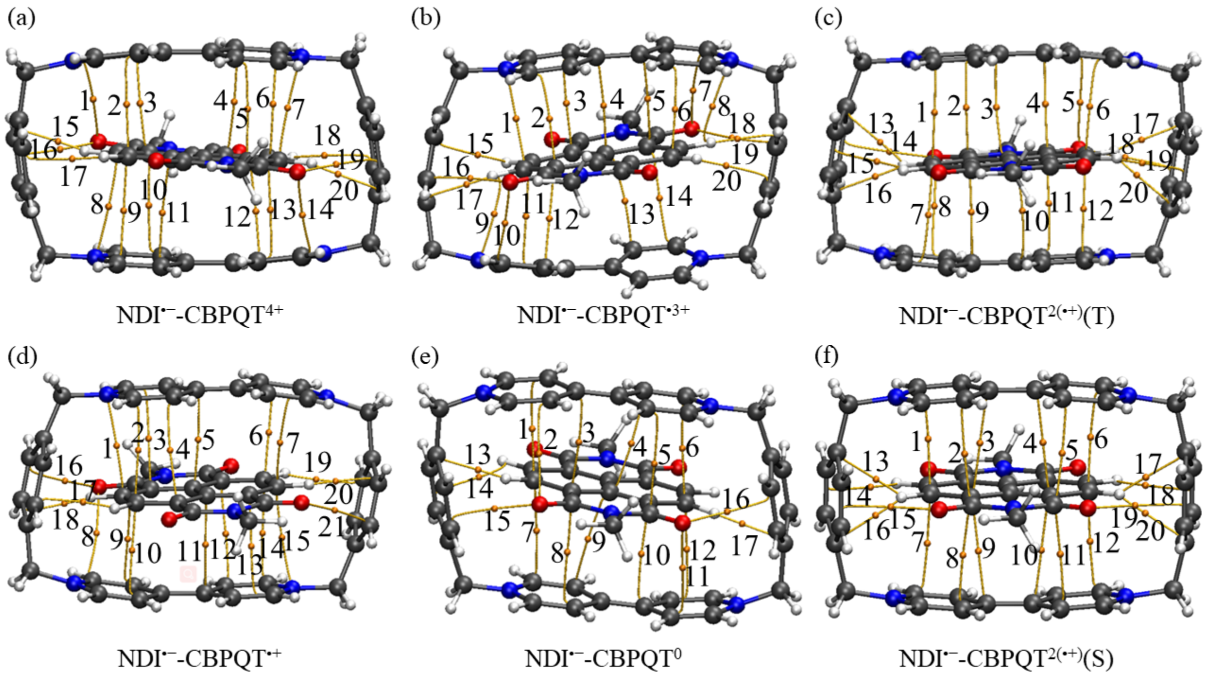

Figure S8. IGM plot iso-surfaces of $NDI^{\bullet-}$-$CBPQT^{n+}$ (n=0-4) complexes. $\delta g^{inter} = 0.005$ a.u. All iso-surfaces are colored according to a BGR scheme over the range $-0.03 < \mathrm{sign}(\lambda_2)\rho < +0.05$ a.u.

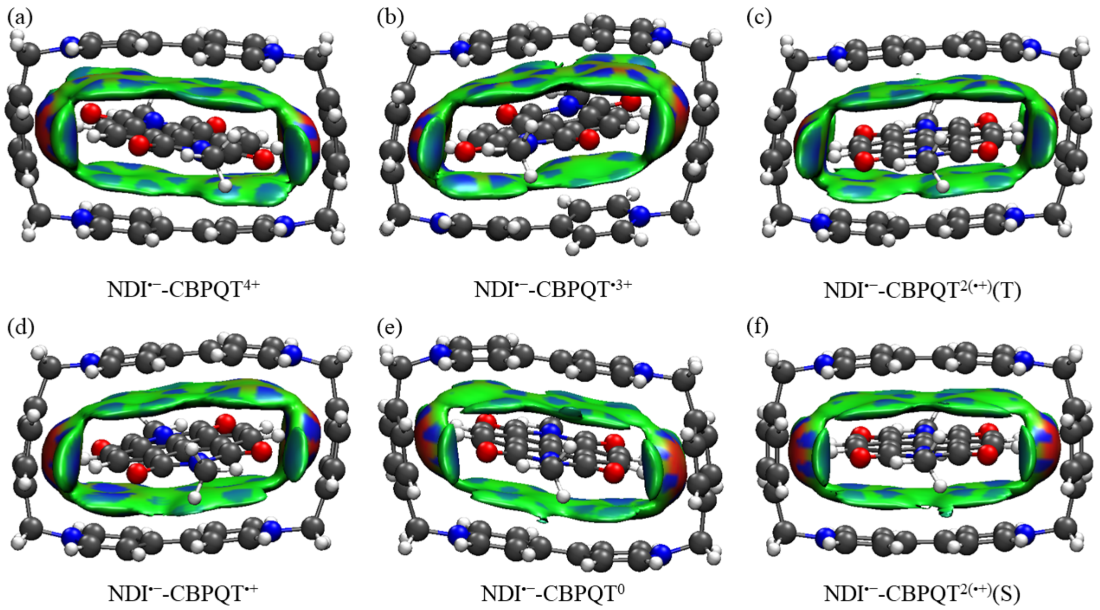

Figure S9. Intermolecular BCPs in a) $BTA^{\bullet}$-$CBPQT^{4+}$ b) $BTA^{\bullet}$-$CBPQT^{\bullet 3+}$ c) $BTA^{\bullet}$-$CBPQT^{2(\bullet +)}$(T) d) $BTA^{\bullet}$-$CBPQT^{\bullet +}$ e) $BTA^{\bullet}$-$CBPQT^{0}$ f) $BTA^{\bullet}$-$CBPQT^{2(\bullet +)}$(S) complexes.

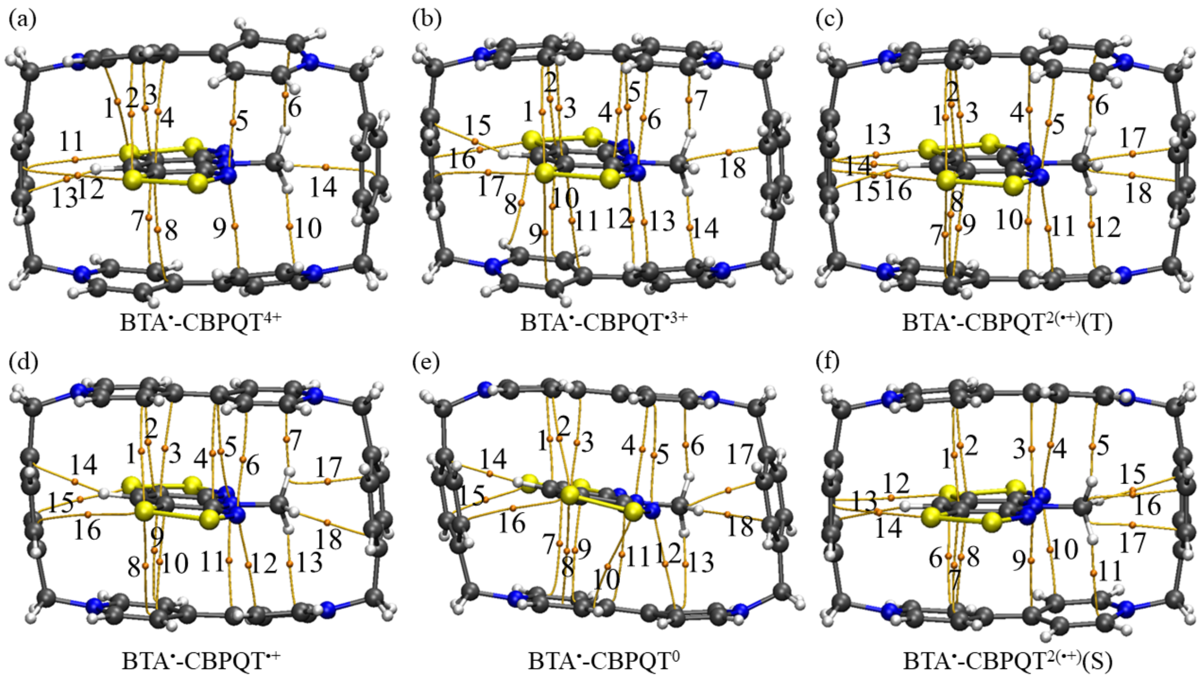

Figure S10. IGM plot iso-surfaces of $BTA^{\bullet}$-$CBPQT^{n+}$ (n=0-4) complexes. δg^{inter} = 0.005 a.u. All iso-surfaces are colored according to a BGR scheme over the range -0.03 < sign(λ_2)ρ < +0.05 a.u.

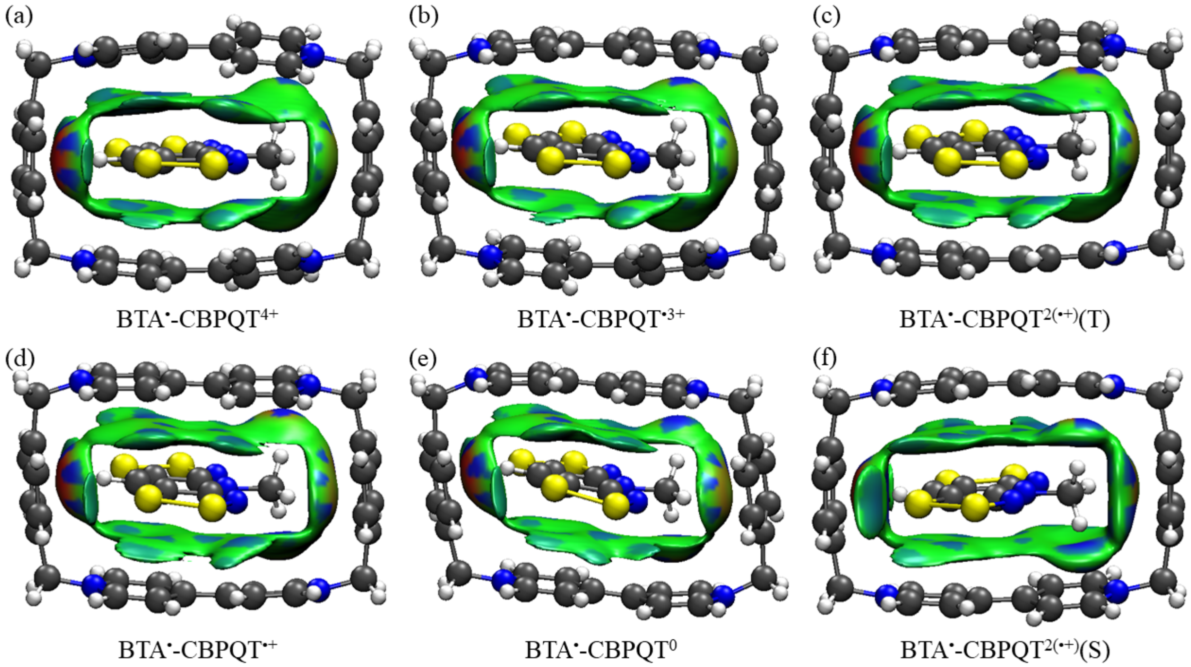

Table S1. AIM analysis results of the intermolecular interactions in the $BIPY^{2+}$-$CBPQT^{n+}$ complexes (a.u.)

	Types	ρ	$\nabla^2\rho$	$\|V\|/G$
$BIPY^{2+}$-$CBPQT^{4+}$	π...π(1)	0.0021	0.0069	0.6742
	π...π(2)	0.0046	0.0126	0.7607
	π...π(3)	0.0030	0.0097	0.7108
	π...π(4)	0.0037	0.0108	0.7610
	π...π(5)	0.0052	0.0144	0.7646
	π...π(6)	0.0022	0.0070	0.7134
	π...π(7)	0.0032	0.0103	0.6948
	C-H...π(8)	0.0071	0.0235	0.7405
	C-H...π(9)	0.0055	0.0181	0.7388
	C-H...π(10)	0.0041	0.0142	0.7088
	C-H...π(11)	0.0047	0.0159	0.6769
	C-H...π(12)	0.0019	0.0063	0.5976
$BIPY^{2+}$-$CBPQT^{\bullet 3+}$	π...π(1)	0.0075	0.0204	0.7924
	π...π(2)	0.0060	0.0162	0.7856
	π...π(3)	0.0058	0.0154	0.7860
	π...π(4)	0.0058	0.0159	0.7836
	π...π(5)	0.0075	0.0206	0.7911
	π...π(6)	0.0043	0.0119	0.7615
	π...π(7)	0.0040	0.0112	0.7572
	C-H...π(8)	0.0044	0.0149	0.7215
	C-H...π(9)	0.0058	0.0179	0.7226
	C-H...π(10)	0.0044	0.0147	0.7182
	C-H...π(11)	0.0050	0.0154	0.7088
$BIPY^{2+}$-$CBPQT^{2(\bullet +)}$(T)	π...π(1)	0.0065	0.0175	0.7894
	π...π(2)	0.0051	0.0143	0.7784
	π...π(3)	0.0053	0.0148	0.7834
	π...π(4)	0.0053	0.0150	0.7789
	π...π(5)	0.0063	0.0172	0.7881
	π...π(6)	0.0063	0.0170	0.7887

	π...π(7)	0.0054	0.0150	0.7795
	π...π(8)	0.0053	0.0146	0.7830
	π...π(9)	0.0051	0.0142	0.7773
	π...π(10)	0.0064	0.0172	0.7887
	C-H...π(11)	0.0032	0.0099	0.6655
	C-H...π(12)	0.0043	0.0143	0.7124
	C-H...π(13)	0.0028	0.0097	0.6590
	C-H...π(14)	0.0027	0.0095	0.6591
	C-H...π(15)	0.0043	0.0142	0.7120
	C-H...π(16)	0.0032	0.0099	0.6647
$BIPY^{2+}$-$CBPQT^{2(\bullet+)}$(S)	π...π(1)	0.0065	0.0179	0.7890
	π...π(2)	0.0064	0.0176	0.7876
	π...π(3)	0.0058	0.0156	0.7878
	π...π(4)	0.0062	0.0173	0.7860
	π...π(5)	0.0063	0.0174	0.7866
	π...π(6)	0.0064	0.0174	0.7867
	π...π(7)	0.0063	0.0175	0.7864
	π...π(8)	0.0059	0.0158	0.7875
	π...π(9)	0.0064	0.0176	0.7864
	π...π(10)	0.0065	0.0178	0.7879
	C-H...π(11)	0.0044	0.0137	0.7056
	C-H...π(12)	0.0034	0.0107	0.6801
	C-H...π(13)	0.0034	0.0106	0.6791
	C-H...π(14)	0.0043	0.0136	0.7049
$BIPY^{2+}$-$CBPQT^{\bullet+}$	π...π(1)	0.0114	0.0306	0.8151
	π...π(2)	0.0090	0.0251	0.8080
	π...π(3)	0.0125	0.0269	0.7961
	π...π(4)	0.0091	0.0253	0.8087
	π...π(5)	0.0114	0.0307	0.8172
	π...π(6)	0.0061	0.0162	0.7994
	π...π(7)	0.0045	0.0126	0.7803

	π...π(8)	0.0049	0.0137	0.8070
	π...π(9)	0.0043	0.0121	0.7783
	π...π(10)	0.0061	0.0161	0.8002
	C-H...π(11)	0.0024	0.0074	0.6407
	C-H...π(12)	0.0042	0.0143	0.7205
	C-H...π(13)	0.0043	0.0144	0.6863
	C-H...π(14)	0.0042	0.0141	0.7204
	C-H...π(15)	0.0044	0.0146	0.6879
	C-H...π(16)	0.0024	0.0074	0.6415
$BIPY^{2+}$-$CBPQT^{0}$	π...π(1)	0.0095	0.0250	0.8077
	π...π(2)	0.0069	0.0196	0.7931
	π...π(3)	0.0097	0.0223	0.8070
	π...π(4)	0.0069	0.0195	0.7954
	π...π(5)	0.0095	0.0252	0.8096
	π...π(6)	0.0095	0.0250	0.8081
	π...π(7)	0.0068	0.0192	0.7936
	π...π(8)	0.0096	0.0221	0.8073
	π...π(9)	0.0069	0.0196	0.7952
	π...π(10)	0.0095	0.0251	0.8089
	C-H...π(11)	0.0059	0.0198	0.7086
	C-H...π(12)	0.0039	0.0137	0.7253
	C-H...π(13)	0.0023	0.0069	0.6382
	C-H...π(14)	0.0023	0.0071	0.6403
	C-H...π(15)	0.0040	0.0140	0.7260
	C-H...π(16)	0.0060	0.0200	0.7093

Table S2. AIM analysis results of the intermolecular interactions in the NDI-CBPQT^{n+} complexes (a.u.)

	Types	ρ	$\nabla^2\rho$	$\|V\|/G$
NDI-CBPQT^{4+}	π...π(1)	0.0064	0.0189	0.7662
	π...π(2)	0.0062	0.0216	0.7166
	π...π(3)	0.0063	0.0183	0.7892
	π...π(4)	0.0061	0.0177	0.7889
	π...π(5)	0.0052	0.0174	0.7069
	π...π(6)	0.0071	0.0208	0.7669
	π...π(7)	0.0069	0.0203	0.7639
	π...π(8)	0.0060	0.0173	0.7885
	π...π(9)	0.0061	0.0176	0.7871
	π...π(10)	0.0064	0.0186	0.7630
	C-H...π(11)	0.0131	0.0490	0.8306
	C-H...π(12)	0.0074	0.0258	0.7171
	lone pair...π(13)	0.0079	0.0260	0.8349
	C-H...π(14)	0.0126	0.0480	0.8190
	C-H...π(15)	0.0126	0.0481	0.8197
	C-H...π(16)	0.0072	0.0251	0.7176
	lone pair...π(17)	0.0079	0.0263	0.8363
	C-H...π(18)	0.0133	0.0496	0.8300
NDI-CBPQT$^{\bullet 3+}$	π...π(1)	0.0070	0.0203	0.7802
	π...π(2)	0.0063	0.0212	0.7273
	π...π(3)	0.0065	0.0195	0.7971
	π...π(4)	0.0063	0.0189	0.7949
	π...π(5)	0.0063	0.0209	0.7270
	π...π(6)	0.0068	0.0199	0.7803
	π...π(7)	0.0071	0.0206	0.7627
	π...π(8)	0.0060	0.0175	0.7869
	π...π(9)	0.0063	0.0180	0.7852
	π...π(10)	0.0058	0.0202	0.7146
	π...π(11)	0.0064	0.0185	0.7635

	C-H...π(12)	0.0131	0.0490	0.8249
	C-H...π(13)	0.0075	0.0262	0.7194
	lone pair...π(14)	0.0075	0.0247	0.8310
	C-H...π(15)	0.0123	0.0474	0.8126
	C-H...π(16)	0.0126	0.0481	0.8163
	C-H...π(17)	0.0075	0.0258	0.7195
	lone pair...π(18)	0.0076	0.0250	0.8323
	C-H...π(19)	0.0129	0.0486	0.8246
NDI-CBPQT$^{2(\bullet+)}$(T)	π...π(1)	0.0064	0.0206	0.8474
	π...π(2)	0.0071	0.0194	0.7940
	π...π(3)	0.0078	0.0216	0.8041
	π...π(4)	0.0059	0.0169	0.7936
	π...π(5)	0.0078	0.0217	0.8040
	π...π(6)	0.0071	0.0195	0.7939
	π...π(7)	0.0064	0.0207	0.8479
	π...π(8)	0.0064	0.0206	0.8472
	π...π(9)	0.0071	0.0194	0.7940
	π...π(10)	0.0079	0.0217	0.8043
	π...π(11)	0.0059	0.0170	0.7936
	π...π(12)	0.0078	0.0217	0.8040
	π...π(13)	0.0071	0.0194	0.7939
	π...π(14)	0.0064	0.0206	0.8482
	C-H...π(15)	0.0096	0.0335	0.7331
	C-H...π(16)	0.0163	0.0622	0.8525
	lone pair...π(17)	0.0079	0.0266	0.8388
	lone pair...π(18)	0.0079	0.0264	0.8381
	C-H...π(19)	0.0163	0.0621	0.8536
	C-H...π(20)	0.0094	0.0330	0.7316
NDI-CBPQT$^{2(\bullet+)}$(S)	π...π(1)	0.0069	0.0200	0.7781
	π...π(2)	0.0060	0.0201	0.7257
	π...π(3)	0.0063	0.0188	0.7945

	π...π(4)	0.0064	0.0190	0.7946
	π...π(5)	0.0063	0.0211	0.7304
	π...π(6)	0.0068	0.0195	0.7782
	π...π(7)	0.0070	0.0200	0.7779
	π...π(8)	0.0058	0.0195	0.7253
	π...π(9)	0.0062	0.0186	0.7941
	π...π(10)	0.0063	0.0187	0.7941
	π...π(11)	0.0061	0.0204	0.7296
	π...π(12)	0.0068	0.0196	0.7779
	C-H...π(13)	0.0125	0.0479	0.8141
	C-H...π(14)	0.0075	0.0259	0.7213
	lone pair...π(15)	0.0074	0.0244	0.8303
	C-H...π(16)	0.0126	0.0480	0.8147
	C-H...π(17)	0.0125	0.0477	0.8144
	C-H...π(18)	0.0077	0.0266	0.7216
	lone pair...π(19)	0.0074	0.0244	0.8310
	C-H...π(20)	0.0125	0.0477	0.8138
NDI-CBPQT$^{\bullet+}$	π...π(1)	0.0063	0.0189	0.8452
	π...π(2)	0.0081	0.0234	0.7583
	π...π(3)	0.0073	0.0227	0.7931
	π...π(4)	0.0106	0.0264	0.8341
	π...π(5)	0.0081	0.0236	0.8718
	π...π(6)	0.0116	0.0310	0.8028
	π...π(7)	0.0073	0.0232	0.8486
	π...π(8)	0.0081	0.0227	0.7861
	π...π(9)	0.0072	0.0207	0.8047
	π...π(10)	0.0058	0.0177	0.7799
	π...π(11)	0.0050	0.0153	0.7379
	π...π(12)	0.0057	0.0165	0.7811
	C-H...π(13)	0.0103	0.0415	0.7620
	C-H...π(14)	0.0078	0.0272	0.7235
	lone pair...π(15)	0.0074	0.0247	0.8337

	C-H...π(16)	0.0138	0.0495	0.8479
	C-H...π(17)	0.0130	0.0469	0.8364
	C-H...π(18)	0.0064	0.0222	0.7285
	lone pair...π(19)	0.0080	0.0266	0.8357
NDI-CBPQT^0	π...π(1)	0.0075	0.0224	0.8735
	π...π(2)	0.0104	0.0289	0.7989
	π...π(3)	0.0092	0.0236	0.8305
	π...π(4)	0.0065	0.0186	0.8620
	π...π(5)	0.0071	0.0212	0.7995
	π...π(6)	0.0064	0.0193	0.7616
	π...π(7)	0.0057	0.0178	0.7894
	π...π(8)	0.0056	0.0176	0.7892
	π...π(9)	0.0064	0.0189	0.7591
	π...π(10)	0.0070	0.0210	0.7991
	π...π(11)	0.0092	0.0235	0.8294
	π...π(12)	0.0075	0.0222	0.8731
	π...π(13)	0.0103	0.0286	0.7989
	C-H...π(14)	0.0133	0.0476	0.8394
	lone pair...π(15)	0.0084	0.0280	0.8458
	C-H...π(16)	0.0067	0.0233	0.7292
	lone pair...π(17)	0.0083	0.0275	0.8449
	C-H...π(18)	0.0067	0.0233	0.7297
	C-H...π(19)	0.0133	0.0477	0.8393

Table S3. AIM analysis results of the intermolecular interactions in the TTF-CBPQT^{n+} complexes (a.u.)

	Types	ρ	$\nabla^2\rho$	$\|V\|/G$
TTF-CBPQT^{4+}	π...π(1)	0.0100	0.0281	0.7547
	π...π(2)	0.0041	0.0104	0.7342
	π...π(3)	0.0037	0.0093	0.7259
	π...π(4)	0.0100	0.0284	0.7566
	π...π(5)	0.0100	0.0284	0.7563
	π...π(6)	0.0038	0.0096	0.7269
	π...π(7)	0.0042	0.0106	0.7345
	π...π(8)	0.0100	0.0282	0.7548
	lone pair...π(9)	0.0035	0.0108	0.6847
	lone pair...π(10)	0.0055	0.0176	0.7425
	lone pair...π(11)	0.0035	0.0108	0.6848
	lone pair...π(12)	0.0055	0.0175	0.7427
TTF-CBPQT$^{\bullet 3+}$	π...π(1)	0.0036	0.0106	0.7209
	π...π(2)	0.0060	0.0184	0.7757
	π...π(3)	0.0061	0.0186	0.7743
	π...π(4)	0.0039	0.0113	0.7403
	π...π(5)	0.0057	0.0166	0.7706
	π...π(6)	0.0070	0.0238	0.7118
	π...π(7)	0.0079	0.0221	0.7738
	π...π(8)	0.0046	0.0116	0.7420
	lone pair...π(9)	0.0056	0.0177	0.7397
	lone pair...π(10)	0.0018	0.0055	0.6315
	lone pair...π(11)	0.0040	0.0126	0.7162
	lone pair...π(12)	0.0033	0.0101	0.6833
TTF-CBPQT$^{2(\bullet +)}$(T)	π...π(1)	0.0079	0.0225	0.7854
	π...π(2)	0.0055	0.0149	0.7538
	π...π(3)	0.0051	0.0144	0.8296
	π...π(4)	0.0052	0.0140	0.7551
	π...π(5)	0.0079	0.0223	0.7854

	π...π(6)	0.0079	0.0224	0.7855
	π...π(7)	0.0052	0.0140	0.7550
	π...π(8)	0.0051	0.0144	0.8297
	π...π(9)	0.0055	0.0150	0.7537
	π...π(10)	0.0079	0.0225	0.7854
	lone pair...π(11)	0.0078	0.0264	0.7784
	lone pair...π(12)	0.0078	0.0263	0.7774
	lone pair...π(13)	0.0078	0.0263	0.7773
	lone pair...π(14)	0.0078	0.0264	0.7784
TTF-CBPQT$^{2(\bullet+)}$(S)	π...π(1)	0.0044	0.0138	0.7190
	π...π(2)	0.0056	0.0170	0.7688
	π...π(3)	0.0056	0.0172	0.7696
	π...π(4)	0.0045	0.0141	0.7197
	π...π(5)	0.0047	0.0131	0.7574
	π...π(6)	0.0056	0.0174	0.7733
	π...π(7)	0.0050	0.0148	0.7858
	π...π(8)	0.0050	0.0146	0.7882
	π...π(9)	0.0055	0.0164	0.7804
	π...π(10)	0.0047	0.0131	0.7584
	lone pair...π(11)	0.0017	0.0053	0.6304
	lone pair...π(12)	0.0053	0.0164	0.7357
	lone pair...π(13)	0.0019	0.0059	0.6439
	lone pair...π(14)	0.0051	0.0158	0.7347
TTF-CBPQT$^{\bullet+}$	π...π(1)	0.0052	0.0141	0.7703
	π...π(2)	0.0058	0.0182	0.7756
	π...π(3)	0.0055	0.0160	0.7984
	π...π(4)	0.0046	0.0128	0.7597
	π...π(5)	0.0057	0.0166	0.7847
	π...π(6)	0.0046	0.0141	0.7205
	π...π(7)	0.0056	0.0169	0.7718
	π...π(8)	0.0042	0.0132	0.7189

	π...π(9)	0.0058	0.0173	0.7810
	lone pair...π(10)	0.0048	0.0149	0.7304
	lone pair...π(11)	0.0022	0.0065	0.6617
	lone pair...π(12)	0.0060	0.0189	0.7476
	lone pair...π(13)	0.0018	0.0054	0.6434
TTF-CBPQT^{0}	π...π(1)	0.0060	0.0193	0.7763
	π...π(2)	0.0052	0.0168	0.7295
	π...π(3)	0.0062	0.0196	0.7757
	π...π(4)	0.0047	0.0141	0.8020
	π...π(5)	0.0047	0.0139	0.8133
	π...π(6)	0.0048	0.0156	0.7184
	π...π(7)	0.0059	0.0181	0.7811
	π...π(8)	0.0023	0.0060	0.7339
	π...π(9)	0.0052	0.0153	0.8012
	π...π(10)	0.0045	0.0142	0.7621
	π...π(11)	0.0062	0.0182	0.8069
	π...π(12)	0.0045	0.0145	0.7628
	π...π(13)	0.0047	0.0129	0.7858
	π...π(14)	0.0024	0.0062	0.7392
	lone pair...π(15)	0.0049	0.0155	0.7358
	lone pair...π(16)	0.0021	0.0062	0.6614
	lone pair...π(17)	0.0058	0.0185	0.7496
	lone pair...π(18)	0.0019	0.0057	0.6547

Table S4. The D_0, ΔG^{TOT} and $\Delta G^{ZPE+GEO}$ values of the $BIPY^{2+}$-$CBPQT^{n+}$ complexes (kcal/mol)

	$BIPY^{2+}$-$CBPQT^{4+}$	$BIPY^{2+}$-$CBPQT^{\bullet 3+}$	$BIPY^{2+}$-$CBPQT^{2(\bullet +)}$(T)	$BIPY^{2+}$-$CBPQT^{2(\bullet +)}$(S)	$BIPY^{2+}$-$CBPQT^{\bullet +}$	$BIPY^{2+}$-$CBPQT^{0}$
ΔG^{TOT}	-0.53	-22.72	-35.09	-35.30	-77.90	-82.06
$\Delta G^{ZPE+GEO}$	4.03	5.76	9.46	9.59	33.98	31.81
D_0		16.96	25.63	25.71	43.92	50.25

Table S5. The D_0, ΔG^{TOT} and $\Delta G^{ZPE+GEO}$ values of the NDI-$CBPQT^{n+}$ complexes (kcal/mol)

	NDI-$CBPQT^{4+}$	NDI-$CBPQT^{\bullet 3+}$	NDI-$CBPQT^{2(\bullet +)}$(T)	NDI-$CBPQT^{2(\bullet +)}$(S)	NDI-$CBPQT^{\bullet +}$	NDI-$CBPQT^{0}$
ΔG^{TOT}	-34.20	-35.46	-31.56	-35.86	-44.80	-45.31
$\Delta G^{ZPE+GEO}$	15.06	10.38	9.02	6.83	14.60	16.48
D_0	19.14	25.08	22.54	29.03	30.20	28.83

Table S6. The D_0, ΔG^{TOT} and $\Delta G^{ZPE+GEO}$ values of the TTF-CBPQT^{n+} complexes (kcal/mol)

	TTF-CBPQT^{4+}	TTF-CBPQT$^{\bullet 3+}$	TTF-CBPQT$^{2(\bullet +)}$(T)	TTF-CBPQT$^{2(\bullet +)}$(S)	TTF-CBPQT$^{\bullet +}$	TTF-CBPQT^{0}
ΔG^{TOT}	-36.78	-33.10	-27.34	-28.06	-26.83	-23.31
$\Delta G^{ZPE+GEO}$	11.84	7.99	3.82	3.46	4.59	4.44
D_0	24.94	25.11	23.52	24.60	22.24	18.87

Table S7. AIM analysis results of the intermolecular interactions in the $BIPY^{\bullet+}$-$CBPQT^{n+}$ complexes (a.u.)

	Types	ρ	$\nabla^2\rho$	$\|V\|/G$
$BIPY^{\bullet+}$-$CBPQT^{4+}$	π...π(1)	0.0068	0.0187	0.7831
	π...π(2)	0.0027	0.0081	0.7402
	π...π(3)	0.0024	0.0075	0.7339
	π...π(4)	0.0070	0.0194	0.7836
	π...π(5)	0.0069	0.0193	0.7833
	π...π(6)	0.0029	0.0087	0.7481
	π...π(7)	0.0027	0.0081	0.7407
	π...π(8)	0.0071	0.0197	0.7856
	C-H...π(9)	0.0041	0.0142	0.7257
	C-H...π(10)	0.0034	0.0123	0.7199
	C-H...π(11)	0.0058	0.0178	0.7234
	C-H...π(12)	0.0053	0.0163	0.7154
	C-H...π(13)	0.0035	0.0120	0.7116
	C-H...π(14)	0.0019	0.0069	0.6509
$BIPY^{\bullet+}$-$CBPQT^{\bullet 3+}$	π...π(1)	0.0073	0.0192	0.8050
	π...π(2)	0.0063	0.0175	0.7928
	π...π(3)	0.0068	0.0171	0.8136
	π...π(4)	0.0064	0.0181	0.7896
	π...π(5)	0.0072	0.0190	0.8040
	π...π(6)	0.0051	0.0136	0.7810
	π...π(7)	0.0061	0.0174	0.7780
	π...π(8)	0.0049	0.0137	0.7808
	π...π(9)	0.0068	0.0199	0.7752
	π...π(10)	0.0043	0.0118	0.7725
	C-H...π(11)	0.0034	0.0105	0.6811
	C-H...π(12)	0.0051	0.0167	0.7277
	C-H...π(13)	0.0030	0.0103	0.6808
	C-H...π(14)	0.0049	0.0155	0.7192
	C-H...π(15)	0.0022	0.0071	0.6359

$BIPY^{\bullet+}$-$CBPQT^{2(\bullet+)}$(T)	π...π(1)	0.0072	0.0188	0.8054
	π...π(2)	0.0056	0.0157	0.7891
	π...π(3)	0.0067	0.0167	0.8137
	π...π(4)	0.0056	0.0156	0.7894
	π...π(5)	0.0074	0.0192	0.8046
	π...π(6)	0.0074	0.0192	0.8045
	π...π(7)	0.0056	0.0156	0.7894
	π...π(8)	0.0067	0.0167	0.8137
	π...π(9)	0.0056	0.0156	0.7891
	π...π(10)	0.0072	0.0188	0.8054
	C-H...π(11)	0.0024	0.0074	0.6452
	C-H...π(12)	0.0042	0.0144	0.7283
	C-H...π(13)	0.0054	0.0180	0.7002
	C-H...π(14)	0.0054	0.0180	0.7004
	C-H...π(15)	0.0042	0.0144	0.7285
	C-H...π(16)	0.0024	0.0074	0.6457
$BIPY^{\bullet+}$-$CBPQT^{2(\bullet+)}$(S)	π...π(1)	0.0076	0.0198	0.8059
	π...π(2)	0.0056	0.0154	0.7912
	π...π(3)	0.0071	0.0174	0.8130
	π...π(4)	0.0062	0.0175	0.7882
	π...π(5)	0.0075	0.0197	0.8048
	π...π(6)	0.0057	0.0155	0.7946
	π...π(7)	0.0049	0.0138	0.7872
	π...π(8)	0.0052	0.0155	0.7943
	π...π(9)	0.0037	0.0109	0.7705
	π...π(10)	0.0059	0.0149	0.8108
	C-H...π(11)	0.0028	0.0085	0.6608
	C-H...π(12)	0.0048	0.0162	0.7277
	C-H...π(13)	0.0045	0.0152	0.6874
	C-H...π(14)	0.0038	0.0134	0.7239
	C-H...π(15)	0.0070	0.0235	0.7244

$BIPY^{\bullet+}$-$CBPQT^{\bullet+}$	π...π(1)	0.0078	0.0202	0.8055
	π...π(2)	0.0060	0.0168	0.7919
	π...π(3)	0.0072	0.0175	0.8141
	π...π(4)	0.0060	0.0168	0.7919
	π...π(5)	0.0078	0.0199	0.8050
	π...π(6)	0.0075	0.0197	0.8115
	π...π(7)	0.0050	0.0140	0.7925
	π...π(8)	0.0071	0.0175	0.8297
	π...π(9)	0.0051	0.0142	0.7927
	π...π(10)	0.0074	0.0195	0.8118
	C-H...π(11)	0.0028	0.0085	0.6666
	C-H...π(12)	0.0044	0.0154	0.7315
	C-H...π(13)	0.0056	0.0187	0.7047
	C-H...π(14)	0.0056	0.0189	0.7054
	C-H...π(15)	0.0044	0.0154	0.7314
	C-H...π(16)	0.0028	0.0085	0.6657
$BIPY^{\bullet+}$-$CBPQT^{0}$	π...π(1)	0.0057	0.0143	0.8251
	π...π(2)	0.0063	0.0184	0.8012
	π...π(3)	0.0058	0.0188	0.7382
	π...π(4)	0.0069	0.0205	0.7914
	π...π(5)	0.0069	0.0205	0.7923
	π...π(6)	0.0059	0.0189	0.7375
	π...π(7)	0.0063	0.0185	0.8011
	π...π(8)	0.0057	0.0142	0.8243
	C-H...π(9)	0.0054	0.0054	0.7027
	C-H...π(10)	0.0034	0.0101	0.6879
	C-H...π(11)	0.0043	0.0150	0.7325
	C-H...π(12)	0.0043	0.0150	0.7323
	C-H...π(13)	0.0034	0.0101	0.6876
	C-H...π(14)	0.0054	0.0178	0.7022

Table S8. AIM analysis results of the intermolecular interactions in the $NDI^{\bullet-}$-$CBPQT^{n+}$ complexes (a.u.)

	Types	ρ	$\nabla^2\rho$	$\lvert V\rvert/G$
$NDI^{\bullet-}$-$CBPQT^{4+}$	π...π(1)	0.0059	0.0230	0.7467
	π...π(2)	0.0082	0.0227	0.7854
	π...π(3)	0.0098	0.0297	0.8022
	π...π(4)	0.0053	0.0152	0.7963
	π...π(5)	0.0080	0.0224	0.7988
	π...π(6)	0.0059	0.0170	0.7839
	π...π(7)	0.0062	0.0189	0.8568
	π...π(8)	0.0062	0.0189	0.8575
	π...π(9)	0.0058	0.0168	0.7839
	π...π(10)	0.0082	0.0228	0.7990
	π...π(11)	0.0054	0.0152	0.7972
	π...π(12)	0.0097	0.0293	0.8036
	π...π(13)	0.0084	0.0230	0.7839
	π...π(14)	0.0059	0.0227	0.7515
	C-H...π(15)	0.0171	0.0628	0.8609
	lone pair...π(16)	0.0085	0.0284	0.8410
	C-H...π(17)	0.0091	0.0317	0.7342
	C-H...π(18)	0.0086	0.0302	0.7309
	lone pair...π(19)	0.0088	0.0296	0.8430
	C-H...π(20)	0.0171	0.0624	0.8652
$NDI^{\bullet-}$-$CBPQT^{\bullet 3+}$	π...π(1)	0.0080	0.0222	0.8858
	π...π(2)	0.0094	0.0253	0.8094
	π...π(3)	0.0085	0.0224	0.8255
	π...π(4)	0.0076	0.0236	0.7971
	π...π(5)	0.0086	0.0258	0.7973
	π...π(6)	0.0114	0.0323	0.7954
	π...π(7)	0.0067	0.0216	0.8326
	π...π(8)	0.0074	0.0275	0.8072
	π...π(9)	0.0081	0.0297	0.7921

	π...π(10)	0.0090	0.0314	0.8223
	π...π(11)	0.0076	0.0197	0.7883
	π...π(12)	0.0090	0.0269	0.8049
	π...π(13)	0.0076	0.0241	0.7801
	π...π(14)	0.0077	0.0218	0.7793
	C-H...π(15)	0.0099	0.0352	0.7469
	lone pair...π(16)	0.0090	0.0306	0.8599
	C-H...π(17)	0.0188	0.0659	0.8960
	lone pair...π(18)	0.0063	0.0220	0.8172
	C-H...π(19)	0.0182	0.0647	0.8737
	C-H...π(20)	0.0101	0.0354	0.7403
$NDI^{\bullet-}$-$CBPQT^{2(\bullet+)}$(T)	π...π(1)	0.0067	0.0196	0.7924
	π...π(2)	0.0062	0.0190	0.7484
	π...π(3)	0.0061	0.0191	0.7832
	π...π(4)	0.0077	0.0216	0.8166
	π...π(5)	0.0070	0.0218	0.8382
	π...π(6)	0.0089	0.0239	0.7938
	π...π(7)	0.0088	0.0237	0.7943
	π...π(8)	0.0071	0.0222	0.8400
	π...π(9)	0.0078	0.0217	0.8176
	π...π(10)	0.0062	0.0192	0.7820
	π...π(11)	0.0062	0.0191	0.7449
	π...π(12)	0.0068	0.0198	0.7921
	C-H...π(13)	0.0108	0.0428	0.7689
	lone pair...π(14)	0.0077	0.0256	0.8321
	C-H...π(15)	0.0073	0.0254	0.7272
	C-H...π(16)	0.0134	0.0489	0.8396
	C-H...π(17)	0.0136	0.0494	0.8420
	lone pair...π(18)	0.0077	0.0255	0.8316
	C-H...π(19)	0.0072	0.0250	0.7276
	C-H...π(20)	0.0108	0.0430	0.7682

$NDI^{\bullet-}$-$CBPQT^{2(\bullet+)}$(S)	π...π(1)	0.0077	0.0212	0.7974
	π...π(2)	0.0061	0.0205	0.7417
	π...π(3)	0.0065	0.0190	0.8069
	π...π(4)	0.0067	0.0193	0.8081
	π...π(5)	0.0060	0.0206	0.7370
	π...π(6)	0.0079	0.0215	0.7976
	π...π(7)	0.0081	0.0221	0.7980
	π...π(8)	0.0063	0.0214	0.7406
	π...π(9)	0.0069	0.0199	0.8098
	π...π(10)	0.0068	0.0195	0.8074
	π...π(11)	0.0061	0.0207	0.7382
	π...π(12)	0.0080	0.0218	0.7978
	C-H...π(13)	0.0124	0.0477	0.8125
	C-H...π(14)	0.0074	0.0256	0.7283
	lone pair...π(15)	0.0079	0.0263	0.8338
	C-H...π(16)	0.0127	0.0483	0.8186
	C-H...π(17)	0.0126	0.0478	0.8171
	C-H...π(18)	0.0073	0.0255	0.7278
	lone pair...π(19)	0.0075	0.0250	0.8291
	C-H...π(20)	0.0123	0.0471	0.8095
$NDI^{\bullet-}$-$CBPQT^{\bullet+}$	π...π(1)	0.0060	0.0179	0.8689
	π...π(2)	0.0062	0.0172	0.8162
	π...π(3)	0.0074	0.0192	0.8283
	π...π(4)	0.0057	0.0156	0.8276
	π...π(5)	0.0067	0.0205	0.7955
	π...π(6)	0.0092	0.0267	0.8137
	π...π(7)	0.0081	0.0246	0.8019
	π...π(8)	0.0046	0.0172	0.7603
	π...π(9)	0.0089	0.0270	0.7865
	π...π(10)	0.0096	0.0274	0.8063
	π...π(11)	0.0065	0.0205	0.7844

	π...π(12)	0.0061	0.0167	0.8163
	π...π(13)	0.0074	0.0196	0.8130
	π...π(14)	0.0060	0.0171	0.8004
	π...π(15)	0.0058	0.0174	0.8599
	lone pair...π(16)	0.0077	0.0260	0.8366
	C-H...π(17)	0.0092	0.0319	0.7485
	C-H...π(18)	0.0171	0.0627	0.8604
	C-H...π(19)	0.0101	0.0353	0.7630
	C-H...π(20)	0.0176	0.0634	0.8744
	lone pair...π(21)	0.0076	0.0259	0.8389
$NDI^{\bullet -}$-$CBPQT^{0}$	π...π(1)	0.0084	0.0224	0.8096
	π...π(2)	0.0064	0.0196	0.8574
	π...π(3)	0.0074	0.0196	0.8350
	π...π(4)	0.0061	0.0181	0.7975
	π...π(5)	0.0055	0.0172	0.7514
	π...π(6)	0.0058	0.0170	0.7964
	π...π(7)	0.0058	0.0169	0.7968
	π...π(8)	0.0055	0.0168	0.7551
	π...π(9)	0.0060	0.0181	0.7970
	π...π(10)	0.0074	0.0196	0.8336
	π...π(11)	0.0064	0.0194	0.8555
	π...π(12)	0.0084	0.0225	0.8092
	C-H...π(13)	0.0140	0.0505	0.8432
	C-H...π(14)	0.0067	0.0233	0.7387
	lone pair...π(15)	0.0092	0.0308	0.8522
	lone pair...π(16)	0.0093	0.0308	0.8528
	C-H...π(17)	0.0140	0.0507	0.8433

Table S9. AIM analysis results of the intermolecular interactions in the BTA$^{•}$-CBPQT^{n+} complexes (a.u.)

	Types	ρ	$\nabla^2\rho$	$|V|/G$
BTA$^{•}$-CBPQT^{4+}	π...π(1)	0.0047	0.0156	0.7007
	π...π(2)	0.0042	0.0119	0.7417
	π...π(3)	0.0071	0.0200	0.7959
	π...π(4)	0.0046	0.0130	0.8010
	π...π(5)	0.0085	0.0248	0.8614
	C-H...π(6)	0.0058	0.0189	0.7034
	π...π(7)	0.0070	0.0198	0.7995
	π...π(8)	0.0047	0.0133	0.8004
	π...π(9)	0.0086	0.0251	0.8595
	C-H...π(10)	0.0062	0.0209	0.7128
	lone pair...π(11)	0.0067	0.0206	0.7504
	lone pair...π(12)	0.0033	0.0103	0.7066
	C-H...π(13)	0.0116	0.0422	0.8134
	C-H...π(14)	0.0084	0.0313	0.7339
BTA$^{•}$-CBPQT$^{•3+}$	π...π(1)	0.0061	0.0178	0.7763
	π...π(2)	0.0072	0.0203	0.8125
	π...π(3)	0.0061	0.0182	0.8043
	π...π(4)	0.0048	0.0164	0.7602
	π...π(5)	0.0049	0.0152	0.8472
	π...π(6)	0.0063	0.0174	0.8682
	C-H...π(7)	0.0086	0.0281	0.7643
	π...π(8)	0.0045	0.0152	0.6691
	π...π(9)	0.0034	0.0096	0.7369
	π...π(10)	0.0070	0.0197	0.7941
	π...π(11)	0.0042	0.0120	0.7978
	π...π(12)	0.0046	0.0148	0.8226
	π...π(13)	0.0097	0.0289	0.8601
	C-H...π(14)	0.0072	0.0236	0.7215
	C-H...π(15)	0.0118	0.0423	0.8189

	lone pair...π(16)	0.0062	0.0190	0.7483
	lone pair...π(17)	0.0035	0.0107	0.7098
	C-H...π(18)	0.0082	0.0294	0.7455
$BTA^{\bullet}$-$CBPQT^{2(\bullet+)}$(T)	π...π(1)	0.0059	0.0167	0.7781
	π...π(2)	0.0070	0.0189	0.8187
	π...π(3)	0.0059	0.0173	0.8091
	π...π(4)	0.0055	0.0168	0.8595
	π...π(5)	0.0073	0.0203	0.8738
	C-H...π(6)	0.0092	0.0294	0.7696
	π...π(7)	0.0058	0.0166	0.7775
	π...π(8)	0.0069	0.0189	0.8186
	π...π(9)	0.0059	0.0174	0.8097
	π...π(10)	0.0053	0.0162	0.8555
	π...π(11)	0.0074	0.0204	0.8736
	C-H...π(12)	0.0100	0.0322	0.7904
	lone pair...π(13)	0.0062	0.0189	0.7487
	C-H...π(14)	0.0105	0.0391	0.7986
	C-H...π(15)	0.0105	0.0391	0.8013
	lone pair...π(16)	0.0034	0.0105	0.7112
	C-H...π(17)	0.0078	0.0284	0.7557
	C-H...π(18)	0.0078	0.0281	0.7527
$BTA^{\bullet}$-$CBPQT^{2(\bullet+)}$(S)	π...π(1)	0.0066	0.0184	0.8196
	π...π(2)	0.0063	0.0181	0.8132
	π...π(3)	0.0058	0.0173	0.8695
	π...π(4)	0.0067	0.0191	0.8653
	C-H...π(5)	0.0096	0.0309	0.7790
	π...π(6)	0.0060	0.0173	0.7788
	π...π(7)	0.0070	0.0190	0.8191
	π...π(8)	0.0061	0.0181	0.8099
	π...π(9)	0.0054	0.0166	0.8603
	π...π(10)	0.0075	0.0210	0.8703

	C-H...π(11)	0.0098	0.0319	0.7892
	lone pair...π(12)	0.0034	0.0105	0.7128
	C-H...π(13)	0.0110	0.0408	0.8070
	lone pair...π(14)	0.0065	0.0201	0.7517
	C-H...π(15)	0.0070	0.0248	0.7541
	C-H...π(16)	0.0078	0.0279	0.7519
	C-H...π(17)	0.0079	0.0280	0.7402
$BTA^{\bullet}$-$CBPQT^{\bullet +}$	π...π(1)	0.0069	0.0195	0.7948
	π...π(2)	0.0071	0.0201	0.8146
	π...π(3)	0.0065	0.0195	0.8096
	π...π(4)	0.0052	0.0168	0.7865
	π...π(5)	0.0052	0.0164	0.8452
	π...π(6)	0.0061	0.0165	0.8730
	C-H...π(7)	0.0110	0.0339	0.8263
	π...π(8)	0.0055	0.0155	0.7810
	π...π(9)	0.0067	0.0178	0.8240
	π...π(10)	0.0057	0.0165	0.8138
	π...π(11)	0.0061	0.0181	0.8734
	π...π(12)	0.0081	0.0227	0.8753
	C-H...π(13)	0.0099	0.0314	0.7729
	C-H...π(14)	0.0114	0.0416	0.8098
	lone pair...π(15)	0.0064	0.0194	0.7545
	lone pair...π(16)	0.0038	0.0115	0.7226
	C-H...π(17)	0.0083	0.0292	0.7350
	C-H...π(18)	0.0077	0.0277	0.7538
$BTA^{\bullet}$-$CBPQT^{0}$	π...π(1)	0.0078	0.0230	0.8050
	π...π(2)	0.0072	0.0199	0.7952
	π...π(3)	0.0073	0.0210	0.8215
	π...π(4)	0.0058	0.0168	0.8916
	π...π(5)	0.0048	0.0133	0.8591
	C-H...π(6)	0.0088	0.0279	0.7793

π...π(7)	0.0065	0.0166	0.8398
π...π(8)	0.0051	0.0141	0.7956
π...π(9)	0.0053	0.0143	0.8287
π...π(10)	0.0036	0.0109	0.7335
π...π(11)	0.0056	0.0184	0.7781
π...π(12)	0.0047	0.0144	0.8353
C-H...π(13)	0.0109	0.0327	0.8088
C-H...π(14)	0.0116	0.0412	0.8189
lone pair...π(15)	0.0065	0.0197	0.7585
lone pair...π(16)	0.0043	0.0127	0.7333
C-H...π(17)	0.0076	0.0274	0.7391
C-H...π(18)	0.0074	0.0269	0.7624

Table S10. The D_0, ΔG^{TOT} and $\Delta G^{ZPE+GEO}$ values of the $BIPY^{\bullet+}$-$CBPQT^{n+}$ complexes (kcal/mol)

	$BIPY^{\bullet+}$-$CBPQT^{4+}$	$BIPY^{\bullet+}$-$CBPQT^{\bullet 3+}$	$BIPY^{\bullet+}$-$CBPQT^{2(\bullet+)}$(T)	$BIPY^{\bullet+}$-$CBPQT^{2(\bullet+)}$(S)	$BIPY^{\bullet+}$-$CBPQT^{\bullet+}$	$BIPY^{\bullet+}$-$CBPQT^{0}$
ΔG^{int}	-23.23	-29.72	-34.39	-33.59	-39.15	-43.05
$\Delta G^{ZPE+GEO}$	1.05	1.67	1.69	3.30	6.10	11.03
D_0	22.18	28.05	32.69	30.29	33.05	32.02

Table S11. The D_0, ΔG^{TOT} and $\Delta G^{ZPE+GEO}$ values of the $NDI^{•−}$-$CBPQT^{n+}$ complexes (kcal/mol)

	$NDI^{•−}$-$CBPQT^{4+}$	$NDI^{•−}$-$CBPQT^{•3+}$	$NDI^{•−}$-$CBPQT^{2(•+)}$(T)	$NDI^{•−}$-$CBPQT^{2(•+)}$(S)	$NDI^{•−}$-$CBPQT^{•+}$	$NDI^{•−}$-$CBPQT^{0}$
ΔG^{TOT}	-57.06	-50.17	-42.90	-41.68	-32.45	-27.92
$\Delta G^{ZPE+GEO}$	30.45	33.18	13.70	12.47	17.60	16.42
D_0	26.61	16.99	29.20	29.21	14.85	11.50

Table S12. The D_0, ΔG^{TOT} and $\Delta G^{ZPE+GEO}$ values of the BTA$^{\bullet}$-CBPQT^{n+} complexes (kcal/mol)

	BTA$^{\bullet}$-CBPQT^{4+}	BTA$^{\bullet}$-CBPQT$^{\bullet 3+}$	BTA$^{\bullet}$-CBPQT$^{2(\bullet +)}$(T)	BTA$^{\bullet}$-CBPQT$^{2(\bullet +)}$(S)	BTA$^{\bullet}$-CBPQT$^{\bullet +}$	BTA$^{\bullet}$-CBPQT^{0}
ΔE^{TOT}	-35.42	-35.57	-35.50	-35.19	-37.57	-38.87
$\Delta E^{ZPE+geo}$	11.05	6.44	3.48	3.31	5.88	9.16
D_0	24.37	29.13	32.02	31.88	31.69	29.71

II. Atomic Cartesian coordinates of all optimized geometries (Å)

$CBPQT^{4+}$

Atom	X	Y	Z
N	2.841668	3.493165	1.355508
N	-2.841669	-3.493165	-1.355506
N	-3.646592	2.905312	-1.122633
N	3.646593	-2.905312	1.122631
C	4.249002	3.317633	1.824716
C	-4.249003	-3.317633	-1.824713
C	1.847940	2.978524	2.106789
C	-1.847942	-2.978523	-2.106788
C	0.548225	2.983506	1.655677
C	-0.548226	-2.983505	-1.655676
C	0.257168	3.519101	0.397599
C	-0.257168	-3.519101	-0.397599
C	1.295969	4.089700	-0.338534
C	-1.295968	-4.089700	0.338535
C	2.581534	4.062331	0.164390
C	-2.581534	-4.062331	-0.164388
C	-1.115425	3.402957	-0.151305
C	1.115425	-3.402957	0.151305
C	-2.237226	3.568465	0.662549
C	2.237226	-3.568464	-0.662550
C	-3.492611	3.311413	0.150931
C	3.492611	-3.311413	-0.150933
C	-2.584776	2.796580	-1.945544
C	2.584777	-2.796580	1.945542
C	-1.310338	3.046654	-1.488757
C	1.310339	-3.046654	1.488757
C	-4.977617	2.430035	-1.604902
C	4.977618	-2.430034	1.604900

C	-4.951882	0.920820	-1.679500
C	4.951882	-0.920820	1.679499
C	-4.927806	0.168466	-0.501018
C	4.927806	-0.168465	0.501018
C	-4.752250	-1.205329	-0.553362
C	4.752250	1.205331	0.553363
C	-4.598914	-1.848701	-1.785101
C	4.598913	1.848701	1.785103
C	-4.679648	-1.107579	-2.961291
C	4.679648	1.107578	2.961292
C	-4.855013	0.274011	-2.908544
C	4.855013	-0.274012	2.908544
H	4.310193	3.729535	2.832893
H	-4.310194	-3.729536	-2.832890
H	4.882413	3.915791	1.169647
H	-4.882414	-3.915791	-1.169643
H	2.130787	2.537911	3.053612
H	-2.130789	-2.537910	-3.053610
H	-0.215539	2.525709	2.271860
H	0.215537	-2.525708	-2.271860
H	1.126428	4.552942	-1.302600
H	-1.126427	-4.552943	1.302600
H	3.422932	4.475334	-0.375913
H	-3.422931	-4.475335	0.375915
H	-2.153442	3.886423	1.694499
H	2.153441	-3.886422	-1.694500
H	-4.390240	3.403404	0.747551
H	4.390239	-3.403403	-0.747553
H	-2.783848	2.473926	-2.959080
H	2.783850	-2.473927	2.959079
H	-0.480433	2.911197	-2.171348
H	0.480434	-2.911198	2.171348

H	-5.158497	2.890096	-2.576718
H	5.158498	-2.890096	2.576715
H	-5.724955	2.801941	-0.903609
H	5.724956	-2.801940	0.903606
H	-5.028024	0.656565	0.464362
H	5.028024	-0.656563	-0.464363
H	-4.715145	-1.774171	0.371264
H	4.715145	1.774173	-0.371262
H	-4.586741	-1.598440	-3.925521
H	4.586742	1.598438	3.925522
H	-4.897667	0.842023	-3.833077
H	4.897668	-0.842025	3.833076

$CBPQT^{\bullet 3+}$

Atom	X	Y	Z
N	2.955799	3.565228	1.193946
N	-2.795842	-3.491624	-1.500667
N	-3.734296	2.904831	-0.973612
N	3.578613	-2.911372	1.259895
C	4.344056	3.341094	1.633954
C	-4.191996	-3.310715	-2.002385
C	1.934629	3.519382	2.099351
C	-1.791120	-2.900835	-2.177416
C	0.630784	3.500553	1.710428
C	-0.508945	-2.902762	-1.678957
C	0.265965	3.497788	0.327031
C	-0.247860	-3.515245	-0.449685
C	1.367456	3.593205	-0.580569
C	-1.294913	-4.164354	0.205379
C	2.653332	3.615017	-0.136411
C	-2.561830	-4.135216	-0.342737

C	-1.082354	3.367432	-0.112480
C	1.099438	-3.396620	0.157953
C	-2.188599	3.309449	0.792310
C	2.254951	-3.476620	-0.621718
C	-3.455169	3.076393	0.351278
C	3.482593	-3.227451	-0.044885
C	-2.716347	3.003984	-1.879136
C	2.484639	-2.880077	2.044851
C	-1.433710	3.240826	-1.493911
C	1.234385	-3.125140	1.521841
C	-5.047806	2.399417	-1.409932
C	4.881368	-2.438318	1.816881
C	-4.986249	0.897757	-1.587161
C	4.888707	-0.928083	1.813719
C	-4.981067	0.062755	-0.465616
C	5.129151	-0.236916	0.624226
C	-4.774279	-1.300843	-0.606711
C	4.999794	1.144617	0.579277
C	-4.570666	-1.854790	-1.873971
C	4.628804	1.857480	1.721721
C	-4.625667	-1.033085	-2.997595
C	4.437355	1.170604	2.919698
C	-4.833325	0.337159	-2.852881
C	4.565016	-0.214601	2.966923
H	4.471761	3.830900	2.601184
H	-4.214094	-3.655320	-3.037130
H	5.003293	3.835243	0.917693
H	-4.831195	-3.963887	-1.408193
H	2.223791	3.493069	3.142492
H	-2.052424	-2.402321	-3.101529
H	-0.114052	3.463234	2.494323
H	0.262815	-2.382370	-2.232329

H	1.218021	3.651374	-1.650357
H	-1.148367	-4.688382	1.141887
H	3.494893	3.672781	-0.815144
H	-3.410188	-4.604227	0.138036
H	-2.057773	3.446828	1.857304
H	2.218168	-3.722831	-1.675816
H	-4.298065	3.017858	1.028454
H	4.404204	-3.254806	-0.611017
H	-2.990361	2.871413	-2.918424
H	2.637278	-2.626874	3.085517
H	-0.690116	3.301724	-2.277417
H	0.375608	-3.052559	2.177532
H	-5.309667	2.901264	-2.343613
H	4.978175	-2.855188	2.819723
H	-5.780623	2.688934	-0.654592
H	5.668393	-2.861144	1.192557
H	-5.123066	0.482236	0.526267
H	5.408775	-0.776244	-0.276667
H	-4.753808	-1.933171	0.276610
H	5.180767	1.667195	-0.355643
H	-4.491258	-1.454230	-3.989968
H	4.176693	1.713046	3.824078
H	-4.856158	0.970180	-3.735310
H	4.403787	-0.734024	3.907265

CBPQT$^{2(\bullet+)}$(Triplet)

Atom	X	Y	Z
N	2.929272	3.582334	1.328557
N	-2.929272	-3.582334	-1.328557
N	-3.694354	2.907328	-1.034077
N	3.694354	-2.907328	1.034077

C	4.310236	3.356880	1.792209
C	-4.310236	-3.356880	-1.792209
C	1.887570	3.504623	2.207257
C	-1.887570	-3.504623	-2.207257
C	0.594158	3.474915	1.785833
C	-0.594158	-3.474914	-1.785833
C	0.262701	3.501798	0.394341
C	-0.262701	-3.501797	-0.394341
C	1.384266	3.634876	-0.483691
C	-1.384266	-3.634876	0.483691
C	2.658476	3.657490	-0.007345
C	-2.658476	-3.657490	0.007345
C	-1.072253	3.364489	-0.081523
C	1.072253	-3.364488	0.081523
C	-2.182379	3.139405	0.792117
C	2.182379	-3.139405	-0.792117
C	-3.431634	2.908710	0.305521
C	3.431634	-2.908710	-0.305521
C	-2.674281	3.164456	-1.904135
C	2.674281	-3.164456	1.904135
C	-1.405040	3.398004	-1.472520
C	1.405040	-3.398004	1.472520
C	-4.990328	2.410474	-1.531672
C	4.990328	-2.410474	1.531672
C	-4.955186	0.903326	-1.654952
C	4.955186	-0.903326	1.654952
C	-5.334799	0.097985	-0.580829
C	5.334799	-0.097984	0.580829
C	-5.171788	-1.281919	-0.643397
C	5.171788	1.281919	0.643397
C	-4.627043	-1.877907	-1.780865
C	4.627043	1.877907	1.780865

C	-4.290088	-1.076295	-2.873112
C	4.290088	1.076295	2.873113
C	-4.452726	0.302577	-2.810535
C	4.452726	-0.302577	2.810535
H	4.396872	3.781157	2.794377
H	-4.396872	-3.781157	-2.794377
H	4.977130	3.916017	1.132912
H	-4.977130	-3.916017	-1.132912
H	2.150964	3.466272	3.256851
H	-2.150964	-3.466272	-3.256851
H	-0.168912	3.424174	2.551060
H	0.168913	-3.424174	-2.551060
H	1.259958	3.703087	-1.556126
H	-1.259958	-3.703087	1.556126
H	3.516418	3.730192	-0.663809
H	-3.516418	-3.730192	0.663809
H	-2.064452	3.123014	1.867323
H	2.064452	-3.123014	-1.867323
H	-4.273863	2.709447	0.955989
H	4.273863	-2.709446	-0.955988
H	-2.933654	3.170397	-2.955445
H	2.933654	-3.170397	2.955445
H	-0.660286	3.603071	-2.230031
H	0.660286	-3.603071	2.230031
H	-5.181268	2.889993	-2.493528
H	5.181268	-2.889993	2.493528
H	-5.762472	2.739159	-0.833003
H	5.762472	-2.739159	0.833004
H	-5.754421	0.547368	0.314925
H	5.754421	-0.547367	-0.314925
H	-5.464887	-1.894823	0.204637
H	5.464888	1.894823	-0.204637

H	-3.889843	-1.527193	-3.777023
H	3.889843	1.527193	3.777023
H	-4.178296	0.912576	-3.667036
H	4.178296	-0.912576	3.667036

$CBPQT^{2(\bullet+)}$(Singlet)

Atom	X	Y	Z
N	2.929290	3.582233	1.328559
N	-2.929290	-3.582233	-1.328558
N	-3.694346	2.907225	-1.034086
N	3.694346	-2.907225	1.034085
C	4.310273	3.356864	1.792228
C	-4.310273	-3.356864	-1.792227
C	1.887586	3.504498	2.207253
C	-1.887586	-3.504498	-2.207253
C	0.594176	3.474746	1.785824
C	-0.594176	-3.474746	-1.785824
C	0.262724	3.501618	0.394333
C	-0.262724	-3.501618	-0.394333
C	1.384288	3.634728	-0.483694
C	-1.384288	-3.634728	0.483694
C	2.658495	3.657385	-0.007342
C	-2.658495	-3.657385	0.007343
C	-1.072231	3.364309	-0.081534
C	1.072231	-3.364309	0.081534
C	-2.182362	3.139259	0.792105
C	2.182362	-3.139259	-0.792106
C	-3.431625	2.908607	0.305511
C	3.431625	-2.908607	-0.305511
C	-2.674267	3.164327	-1.904143
C	2.674267	-3.164327	1.904143

C	-1.405017	3.397833	-1.472528
C	1.405017	-3.397833	1.472528
C	-4.990359	2.410450	-1.531688
C	4.990360	-2.410450	1.531687
C	-4.955240	0.903317	-1.654975
C	4.955240	-0.903317	1.654975
C	-5.334815	0.097977	-0.580830
C	5.334815	-0.097977	0.580830
C	-5.171805	-1.281919	-0.643399
C	5.171805	1.281920	0.643399
C	-4.627099	-1.877911	-1.780891
C	4.627098	1.877911	1.780891
C	-4.290148	-1.076298	-2.873144
C	4.290148	1.076298	2.873144
C	-4.452785	0.302569	-2.810565
C	4.452785	-0.302569	2.810565
H	4.396874	3.781165	2.794390
H	-4.396874	-3.781165	-2.794389
H	4.977136	3.916027	1.132921
H	-4.977137	-3.916027	-1.132920
H	2.150976	3.466160	3.256848
H	-2.150976	-3.466160	-3.256848
H	-0.168895	3.423994	2.551050
H	0.168895	-3.423994	-2.551050
H	1.259982	3.702949	-1.556129
H	-1.259981	-3.702949	1.556129
H	3.516437	3.730120	-0.663803
H	-3.516437	-3.730120	0.663804
H	-2.064439	3.122882	1.867312
H	2.064439	-3.122882	-1.867313
H	-4.273861	2.709379	0.955980
H	4.273861	-2.709378	-0.955980

H	-2.933641	3.170279	-2.955452
H	2.933641	-3.170279	2.955452
H	-0.660259	3.602886	-2.230040
H	0.660260	-3.602886	2.230040
H	-5.181274	2.889999	-2.493535
H	5.181274	-2.889999	2.493534
H	-5.762478	2.739165	-0.833004
H	5.762478	-2.739165	0.833003
H	-5.754433	0.547369	0.314922
H	5.754433	-0.547368	-0.314922
H	-5.464896	-1.894833	0.204632
H	5.464896	1.894834	-0.204631
H	-3.889937	-1.527207	-3.777065
H	3.889937	1.527206	3.777065
H	-4.178389	0.912574	-3.667073
H	4.178389	-0.912574	3.667073

CBPQT$^{\bullet+}$

Atom	X	Y	Z
N	2.949262	3.697697	1.370740
N	-2.926506	-3.577496	-1.328823
N	-3.747511	3.012734	-1.041124
N	3.692813	-2.906451	1.031159
N	4.308015	3.392519	1.809903
N	-4.302744	-3.331717	-1.797034
N	1.885764	3.465678	2.233279
N	-1.882152	-3.537978	-2.206229
N	0.614217	3.335335	1.810782
N	-0.589117	-3.522109	-1.782320
N	0.247482	3.388801	0.393600
N	-0.261875	-3.519168	-0.389700

N	1.409109	3.603982	-0.472381
N	-1.387444	-3.614153	0.488291
N	2.658911	3.725599	0.013050
N	-2.660320	-3.627659	0.008930
N	-1.036709	3.254446	-0.069448
N	1.072715	-3.384155	0.086764
N	-2.197633	3.034932	0.796348
N	2.194440	-3.241467	-0.789423
N	-3.446811	2.908564	0.310877
N	3.443573	-2.999243	-0.307228
N	-2.675908	3.180227	-1.908566
N	2.661812	-3.090432	1.906654
N	-1.404500	3.313283	-1.486092
N	1.393262	-3.334117	1.480143
N	-4.997331	2.445960	-1.541049
N	4.985168	-2.385732	1.513869
N	-4.934868	0.936482	-1.656851
N	4.930504	-0.879489	1.631753
N	-5.329069	0.125582	-0.591604
N	5.233336	-0.074403	0.532368
N	-5.165043	-1.254669	-0.652288
N	5.070546	1.304573	0.603921
N	-4.603055	-1.850037	-1.781677
N	4.601858	1.906521	1.773367
N	-4.238443	-1.044517	-2.862424
N	4.330033	1.101652	2.881197
N	-4.403281	0.334196	-2.799482
N	4.492674	-0.277935	2.812273
N	4.430346	3.784316	2.824132
N	-4.389581	-3.752372	-2.800771
N	5.000114	3.938803	1.162233
N	-4.978760	-3.885538	-1.142216

N	2.146930	3.423072	3.285424
N	-2.142880	-3.515827	-3.257039
N	-0.144683	3.200061	2.572718
N	0.176513	-3.496364	-2.546218
N	1.288911	3.685183	-1.546386
N	-1.266895	-3.663914	1.562151
N	3.513025	3.883481	-0.637016
N	-3.521234	-3.672263	0.664058
N	-2.079781	2.973988	1.871960
N	2.086844	-3.306479	-1.863877
N	-4.299598	2.741798	0.960429
N	4.296436	-2.865611	-0.960671
N	-2.937128	3.227232	-2.960552
N	2.912193	-3.024065	2.958005
N	-0.649901	3.480795	-2.245881
N	0.638117	-3.471563	2.242616
N	-5.204893	2.900713	-2.514020
N	5.193713	-2.858127	2.475883
N	-5.799729	2.745790	-0.860268
N	5.753595	-2.706585	0.807796
N	-5.765944	0.574495	0.296532
N	5.597936	-0.524943	-0.387085
N	-5.475079	-1.869741	0.188576
N	5.309213	1.917189	-0.261582
N	-3.819523	-1.494295	-3.758800
N	3.984725	1.554513	3.806914
N	-4.111748	0.947646	-3.648083
N	4.273573	-0.887227	3.685513

CBPQT^0

Atom	X	Y	Z

N	2.950891	3.695660	1.353646
N	-2.950891	-3.695659	-1.353646
N	-3.750404	3.019677	-1.045441
N	3.750404	-3.019677	1.045441
C	4.302785	3.365967	1.797592
C	-4.302785	-3.365967	-1.797592
C	1.883453	3.507536	2.221450
C	-1.883452	-3.507536	-2.221450
C	0.609508	3.390465	1.802298
C	-0.609508	-3.390465	-1.802298
C	0.244436	3.411098	0.383892
C	-0.244436	-3.411098	-0.383892
C	1.409449	3.582462	-0.487193
C	-1.409449	-3.582461	0.487193
C	2.661137	3.692748	-0.004298
C	-2.661137	-3.692747	0.004298
C	-1.040896	3.281142	-0.076483
C	1.040896	-3.281142	0.076483
C	-2.204817	3.099405	0.793936
C	2.204817	-3.099405	-0.793936
C	-3.453140	2.960569	0.309715
C	3.453140	-2.960569	-0.309715
C	-2.677060	3.161536	-1.915127
C	2.677060	-3.161536	1.915127
C	-1.406185	3.304187	-1.494758
C	1.406185	-3.304187	1.494758
C	-4.992501	2.424499	-1.532501
C	4.992501	-2.424499	1.532501
C	-4.908450	0.915704	-1.637009
C	4.908450	-0.915704	1.637009
C	-5.253673	0.107410	-0.552388
C	5.253673	-0.107410	0.552389

C	-5.089014	-1.272659	-0.615652
C	5.089014	1.272659	0.615653
C	-4.575792	-1.876372	-1.764817
C	4.575792	1.876372	1.764817
C	-4.249427	-1.069714	-2.857092
C	4.249427	1.069714	2.857092
C	-4.413869	0.309858	-2.793802
C	4.413869	-0.309858	2.793802
H	4.426819	3.758728	2.811336
H	-4.426819	-3.758728	-2.811336
H	5.006377	3.900059	1.151742
H	-5.006377	-3.900059	-1.151742
H	2.143981	3.488859	3.274542
H	-2.143981	-3.488859	-3.274542
H	-0.152044	3.290478	2.566996
H	0.152044	-3.290478	-2.566996
H	1.290759	3.635309	-1.563170
H	-1.290759	-3.635309	1.563170
H	3.518723	3.813692	-0.657792
H	-3.518723	-3.813692	0.657792
H	-2.089797	3.077712	1.871425
H	2.089797	-3.077712	-1.871425
H	-4.308354	2.820466	0.962440
H	4.308354	-2.820466	-0.962440
H	-2.936940	3.177828	-2.968421
H	2.936940	-3.177828	2.968421
H	-0.649890	3.449894	-2.257383
H	0.649890	-3.449894	2.257383
H	-5.213965	2.867201	-2.508250
H	5.213965	-2.867201	2.508250
H	-5.794956	2.718209	-0.849020
H	5.794956	-2.718209	0.849021

H	-5.658709	0.558937	0.349858
H	5.658709	-0.558937	-0.349858
H	-5.365837	-1.885864	0.238507
H	5.365837	1.885864	-0.238507
H	-3.864592	-1.523214	-3.767138
H	3.864592	1.523214	3.767139
H	-4.156586	0.920720	-3.655744
H	4.156586	-0.920720	3.655744

$BIPY^{2+}$

Atom	X	Y	Z
N	2.439425	1.062753	2.271946
C	2.317489	1.681600	1.080941
H	3.006054	2.492898	0.884539
C	1.362628	1.281704	0.172103
H	1.307433	1.790128	-0.782582
C	0.512424	0.219395	0.489027
C	0.660181	-0.404566	1.728317
H	0.018675	-1.221085	2.036593
C	1.630574	0.039077	2.602695
H	1.779844	-0.408162	3.576147
C	3.454814	1.546737	3.228660
H	4.383502	1.733575	2.691545
H	3.617835	0.785569	3.988401
N	-2.443023	-1.070353	-2.264035
C	-1.726334	-1.984079	-1.581196
H	-1.945477	-3.024231	-1.782502
C	-0.761535	-1.592915	-0.678074
H	-0.196284	-2.356750	-0.158692
C	-0.520923	-0.232992	-0.472799
C	-1.274866	0.692926	-1.195898

H	-1.145779	1.759951	-1.063731
C	-2.230501	0.247036	-2.084128
H	-2.843806	0.922871	-2.664642
C	-3.442104	-1.523723	-3.252342
H	-3.907030	-2.438175	-2.888892
H	-4.198391	-0.750251	-3.370284
H	3.089779	2.465010	3.689261
H	-2.936521	-1.705574	-4.201228

NDI

Atom	X	Y	Z
C	-1.499826	1.131781	0.069785
C	-0.802298	-0.096226	0.062997
C	-0.809137	2.323194	0.049514
C	-2.984278	1.132285	0.099147
C	0.607668	-0.091783	0.035097
C	-1.493198	-1.327928	0.083556
C	1.297246	1.140536	0.014988
C	1.306531	-1.319150	0.027821
C	0.598862	2.327627	0.022029
C	2.781700	1.150251	-0.014007
C	-2.979352	-1.345956	0.113798
C	-0.793419	-2.514383	0.076213
C	2.792762	-1.327991	-0.002050
C	0.614449	-2.509945	0.048000
C	4.890506	-0.045939	-0.049085
C	-5.085236	-0.077129	0.149579
H	-1.336241	-3.452540	0.092399
H	1.163373	-3.444676	0.042101
H	5.228357	0.479009	-0.944256
H	-1.358774	3.257600	0.054935

H	1.142406	3.265460	0.006197
H	-5.441449	-1.103470	0.175143
H	-5.462492	0.428848	-0.740905
H	-5.425311	0.460675	1.036201
H	5.253906	-1.070036	-0.056846
H	5.263044	0.477490	0.833211
N	3.428812	-0.084751	-0.020853
N	-3.623350	-0.106680	0.120409
O	3.429947	-2.366135	-0.009603
O	-3.609986	-2.387960	0.132008
O	3.428806	2.182474	-0.031116
O	-3.638027	2.160450	0.104590

TTF

Atom	X	Y	Z
C	-1.652175	4.754528	-0.071950
C	0.373421	6.350302	-0.420228
C	-0.693041	7.148786	-0.418015
H	1.397207	6.689637	-0.517399
H	-0.656063	8.226820	-0.513397
C	-2.439003	3.703119	0.216269
C	-3.348392	1.378556	0.951389
C	-4.414175	2.177938	0.953194
H	-3.360218	0.334333	1.237877
H	-5.412811	1.874008	1.241461
S	-2.279059	6.404539	-0.276098
S	0.106743	4.618587	-0.281715
S	-4.197780	3.840644	0.425939
S	-1.813614	2.052713	0.422083

$BIPY^{\bullet +}$

Atom	X	Y	Z
N	2.485336	1.074816	2.281458
C	2.056748	1.899178	1.281981
H	2.521725	2.876173	1.240430
C	1.104970	1.511047	0.389700
H	0.830852	2.225873	-0.374787
C	0.498868	0.217131	0.459575
C	0.976968	-0.606981	1.526684
H	0.598795	-1.608492	1.683088
C	1.932164	-0.167580	2.391152
H	2.300327	-0.781761	3.203329
C	3.459083	1.545698	3.265977
H	4.146152	2.244015	2.787738
H	4.026006	0.695634	3.645593
N	-2.484763	-1.074219	-2.281511
C	-2.055918	-1.898715	-1.282253
H	-2.520693	-2.875813	-1.240881
C	-1.104125	-1.510592	-0.389986
H	-0.829740	-2.225554	0.374279
C	-0.498261	-0.216554	-0.459658
C	-0.976608	0.607688	-1.526556
H	-0.598690	1.609327	-1.682750
C	-1.931824	0.168297	-2.391007
H	-2.300205	0.782593	-3.202997
C	-3.458538	-1.545075	-3.266015
H	-4.145506	-2.243514	-2.787809
H	-4.025569	-0.695017	-3.645484
H	2.948393	2.043370	4.094261
H	-2.947862	-2.042596	-4.094398

$NDI^{\bullet -}$

Atom	X	Y	Z
C	-1.505820	1.133534	0.069909
C	-0.812120	-0.095910	0.063284
C	-0.794568	2.348589	0.049223
C	-2.961414	1.138781	0.098023
C	0.617544	-0.091340	0.034770
C	-1.499851	-1.329316	0.084121
C	1.303183	1.142474	0.014894
C	1.313324	-1.320388	0.027179
C	0.583950	2.353002	0.022461
C	2.758754	1.156942	-0.013158
C	-2.955565	-1.352137	0.114050
C	-0.778282	-2.539343	0.076071
C	2.769119	-1.333990	-0.002139
C	0.599713	-2.534945	0.047909
C	4.861374	-0.047139	-0.048330
C	-5.056137	-0.077950	0.148856
H	-1.330314	-3.472776	0.092358
H	1.157860	-3.464860	0.041503
H	5.205887	0.478812	-0.941503
H	-1.353681	3.278033	0.054904
H	1.136944	3.285981	0.006772
H	-5.408852	-1.105777	0.175812
H	-5.440063	0.428031	-0.739939
H	-5.402208	0.462965	1.032381
H	5.220161	-1.073241	-0.055428
H	5.240585	0.477863	0.831338
N	3.403820	-0.083743	-0.020175
N	-3.598390	-0.105898	0.119499
O	3.435045	-2.372116	-0.011116
O	-3.614618	-2.394472	0.134030

O	3.433871	2.188892	-0.030579
O	-3.643252	2.166442	0.104019

BTA$^{\bullet}$

Atom	X	Y	Z
S	-3.715215	0.360603	-0.000253
S	-2.785175	-1.549294	-0.005687
N	0.005400	1.287054	-0.004461
N	-2.326301	1.285759	0.000809
C	-0.007811	-1.541174	-0.004029
C	0.052832	2.745587	-0.006412
C	-1.208449	0.619357	-0.002298
C	-1.196960	-0.823778	-0.004196
H	-0.015360	-2.624906	-0.005152
H	-0.965703	3.124620	-0.029539
H	0.601219	3.092903	-0.885109
S	2.773831	-1.570507	0.000631
S	3.711488	0.338547	0.007933
N	2.323879	1.270821	0.005624
C	1.187646	-0.840036	-0.001554
C	1.207257	0.603248	0.000265
H	0.563200	3.098991	0.892760

BIPY^{2+}-CBPQT^{4+}

Atom	X	Y	Z
N	2.846184	3.567802	1.387794
N	-2.614710	-3.636715	-1.716942
N	-3.521115	3.006820	-1.323581
N	3.953651	-3.042584	0.469308
C	4.229254	3.253256	1.855597
C	-4.030646	-3.346240	-2.094357

C	1.802515	3.144152	2.127105
C	-1.633389	-3.295967	-2.574706
C	0.515563	3.234982	1.647463
C	-0.310358	-3.389101	-2.209086
C	0.284142	3.764911	0.373152
C	0.034127	-3.848407	-0.932007
C	1.374576	4.279755	-0.330862
C	-1.002897	-4.281324	-0.101970
C	2.645660	4.163311	0.197128
C	-2.315644	-4.154970	-0.512372
C	-1.066473	3.653845	-0.234832
C	1.438056	-3.738791	-0.449070
C	-2.222897	3.661523	0.548749
C	2.503494	-3.548706	-1.334905
C	-3.435798	3.329102	-0.019184
C	3.744311	-3.187500	-0.851959
C	-2.438898	3.086856	-2.122397
C	2.981023	-3.339820	1.351243
C	-1.204220	3.413128	-1.605983
C	1.720547	-3.691407	0.920869
C	-4.783965	2.429580	-1.872425
C	5.218070	-2.426521	0.966286
C	-4.662617	0.922512	-1.945984
C	5.008414	-0.946626	1.209890
C	-4.780157	0.148761	-0.786751
C	5.163317	-0.029013	0.166838
C	-4.590335	-1.227345	-0.834765
C	4.928520	1.324829	0.379413
C	-4.292568	-1.856509	-2.048030
C	4.530555	1.785532	1.638162
C	-4.221907	-1.090117	-3.211173
C	4.401092	0.873085	2.685526

C	-4.398862	0.291479	-3.159965
C	4.638812	-0.483256	2.472562
H	4.284282	3.528372	2.909377
H	-4.186154	-3.756695	-3.092729
H	4.909729	3.896525	1.298068
H	-4.665671	-3.893719	-1.398614
H	2.027944	2.693998	3.084480
H	-1.937339	-2.910027	-3.538910
H	-0.283939	2.840374	2.262044
H	0.428993	-3.054287	-2.924144
H	1.263545	4.755339	-1.297151
H	-0.826103	-4.706883	0.876935
H	3.522215	4.514814	-0.330272
H	-3.144487	-4.446898	0.118781
H	-2.202123	3.901601	1.604401
H	2.392328	-3.644523	-2.406679
H	-4.349712	3.287116	0.558423
H	4.579261	-2.977891	-1.507340
H	-2.581537	2.843953	-3.167358
H	3.224912	-3.243961	2.401291
H	-0.356288	3.421758	-2.279562
H	0.965009	-3.858979	1.676980
H	-4.947235	2.875530	-2.853600
H	5.498196	-2.955702	1.877590
H	-5.594731	2.745951	-1.215852
H	5.984695	-2.612876	0.214335
H	-5.032244	0.617179	0.161018
H	5.483881	-0.364971	-0.815169
H	-4.686381	-1.808606	0.077597
H	5.071269	2.024733	-0.438827
H	-4.033428	-1.564571	-4.169664
H	4.132263	1.213390	3.681267

H	-4.335932	0.869113	-4.077086
H	4.547139	-1.173636	3.305561
N	-1.649014	-0.702152	3.870530
C	-2.451150	-0.333717	2.853839
H	-3.501873	-0.209931	3.080863
C	-1.939013	-0.123662	1.593000
H	-2.623758	0.191069	0.818204
C	-0.572718	-0.292013	1.352341
C	0.231078	-0.672994	2.429009
H	1.297701	-0.821087	2.333574
C	-0.330101	-0.872488	3.673440
H	0.259133	-1.170172	4.530220
C	-2.239010	-0.945835	5.202518
H	-2.978088	-0.172265	5.404055
H	-1.450903	-0.905957	5.951092
N	1.049116	0.345548	-2.534715
C	1.856220	0.392822	-1.458821
H	2.902582	0.592533	-1.647334
C	1.358272	0.194555	-0.190118
H	2.059972	0.254098	0.633060
C	-0.003810	-0.065857	-0.002616
C	-0.812757	-0.111349	-1.141637
H	-1.872742	-0.331240	-1.089911
C	-0.264781	0.101423	-2.390247
H	-0.859864	0.077185	-3.293659
C	1.637453	0.550126	-3.874075
H	2.348577	-0.253343	-4.065251
H	0.843755	0.531029	-4.617010
H	-2.708485	-1.929828	5.199028
H	2.138891	1.517155	-3.888701

$BIPY^{2+}$-$CBPQT^{\bullet 3+}$

Atom	X	Y	Z
N	2.937793	3.607844	1.283319
N	-2.667700	-3.626487	-1.745124
N	-3.720215	2.863216	-0.876783
N	3.576704	-2.984367	1.246012
C	4.326069	3.344493	1.710464
C	-4.032155	-3.354889	-2.281510
C	1.930418	3.548594	2.196031
C	-1.598352	-3.231082	-2.461842
C	0.621015	3.527103	1.813784
C	-0.333726	-3.274021	-1.920246
C	0.257883	3.540722	0.437564
C	-0.157299	-3.722270	-0.607599
C	1.340452	3.682819	-0.475901
C	-1.272900	-4.202126	0.081116
C	2.633297	3.701407	-0.039683
C	-2.517386	-4.139481	-0.510115
C	-1.100646	3.385040	-0.000186
C	1.163075	-3.582615	0.052831
C	-2.170531	3.137110	0.905912
C	2.354541	-3.647017	-0.672047
C	-3.431560	2.877156	0.453113
C	3.547075	-3.338203	-0.051864
C	-2.742102	3.163240	-1.774031
C	2.451752	-2.983369	1.986069
C	-1.465576	3.430943	-1.375312
C	1.234449	-3.281589	1.416573
C	-5.002785	2.312820	-1.357306
C	4.832924	-2.453184	1.849139
C	-4.848605	0.830553	-1.622511
C	4.788698	-0.942283	1.839508

C	-4.919721	-0.082071	-0.565546
C	4.999792	-0.244350	0.647322
C	-4.681872	-1.432680	-0.784393
C	4.883804	1.139683	0.615195
C	-4.368163	-1.896470	-2.065273
C	4.559066	1.850249	1.774583
C	-4.322849	-0.992654	-3.125397
C	4.379242	1.154978	2.969950
C	-4.563597	0.362369	-2.904591
C	4.489954	-0.233838	3.002429
H	4.473161	3.817600	2.682637
H	-4.029210	-3.624387	-3.337942
H	4.992766	3.827960	0.994743
H	-4.720861	-4.021014	-1.761489
H	2.226243	3.508659	3.236652
H	-1.789830	-2.850561	-3.456464
H	-0.119839	3.490808	2.601208
H	0.492477	-2.912724	-2.519567
H	1.182350	3.764598	-1.542526
H	-1.199150	-4.617660	1.078153
H	3.469200	3.778326	-0.722861
H	-3.413336	-4.475220	-0.005145
H	-2.023588	3.129540	1.977374
H	2.378972	-3.924846	-1.718120
H	-4.250839	2.660027	1.126104
H	4.491330	-3.350469	-0.579356
H	-3.031192	3.165960	-2.817467
H	2.553493	-2.706833	3.027288
H	-0.748932	3.668545	-2.149968
H	0.350505	-3.227529	2.039559
H	-5.283622	2.856959	-2.260432
H	4.906362	-2.856814	2.859262

H	-5.757280	2.511463	-0.594504
H	5.664053	-2.847293	1.264203
H	-5.167648	0.257477	0.436214
H	5.259803	-0.775566	-0.263997
H	-4.746874	-2.123426	0.051343
H	5.053919	1.665376	-0.320024
H	-4.104862	-1.337821	-4.131802
H	4.151139	1.691918	3.886264
H	-4.528835	1.052666	-3.742367
H	4.344700	-0.757953	3.942515
N	-0.784039	-0.028007	3.434772
C	0.485013	0.210225	3.042962
H	1.218921	0.340951	3.828081
C	0.813479	0.283736	1.710622
H	1.851281	0.479473	1.473175
C	-0.173575	0.121158	0.724400
C	-1.474968	-0.151964	1.170441
H	-2.295345	-0.337518	0.490158
C	-1.752039	-0.216580	2.517331
H	-2.747731	-0.417527	2.889300
C	-1.087833	-0.082537	4.876577
H	-0.540745	-0.913735	5.321447
H	-2.157005	-0.230564	5.008884
N	0.710822	0.558682	-3.417198
C	-0.565280	0.407733	-3.015444
H	-1.326983	0.423481	-3.783650
C	-0.874593	0.244085	-1.684304
H	-1.922049	0.128040	-1.437487
C	0.137073	0.244610	-0.712731
C	1.457523	0.379985	-1.173099
H	2.310346	0.346735	-0.508295
C	1.713993	0.539197	-2.514076

H	2.717730	0.654287	-2.902837
C	1.031336	0.752964	-4.843019
H	0.114896	0.681893	-5.424201
H	1.476958	1.739438	-4.971187
H	-0.786484	0.859533	5.334445
H	1.729608	-0.022738	-5.156389

$BIPY^{2+}$-$CBPQT^{2(\bullet+)}$(T)

Atom	X	Y	Z
N	2.920036	3.559052	1.206661
N	-2.944663	-3.584786	-1.331584
N	-3.792654	2.824134	-0.851263
N	3.773083	-2.850279	0.709092
C	4.330266	3.375978	1.598146
C	-4.355436	-3.402328	-1.721306
C	1.931258	3.391150	2.129198
C	-1.957720	-3.420809	-2.256824
C	0.621109	3.305907	1.766627
C	-0.646747	-3.335645	-1.897478
C	0.227792	3.374295	0.396460
C	-0.250360	-3.400686	-0.528050
C	1.292696	3.588203	-0.528237
C	-1.313498	-3.609928	0.399739
C	2.587187	3.663344	-0.110384
C	-2.609078	-3.684743	-0.014966
C	-1.130747	3.227225	-0.020400
C	1.109332	-3.253877	-0.115053
C	-2.197202	3.014944	0.902966
C	2.172838	-3.038205	-1.041066
C	-3.474111	2.812087	0.473218
C	3.450689	-2.834735	-0.614444

C	-2.811862	3.062233	-1.766895
C	2.795625	-3.094070	1.626927
C	-1.518359	3.267794	-1.393214
C	1.501285	-3.299649	1.256454
C	-5.113633	2.341560	-1.296672
C	5.094114	-2.365823	1.152107
C	-5.066041	0.838978	-1.454450
C	5.044073	-0.863781	1.314545
C	-5.412358	0.001235	-0.393660
C	5.387933	-0.021890	0.256277
C	-5.230705	-1.375299	-0.496202
C	5.205445	1.354128	0.364185
C	-4.700623	-1.932003	-1.660570
C	4.676618	1.906174	1.531346
C	-4.394302	-1.097073	-2.737372
C	4.372131	1.067004	2.605379
C	-4.575486	0.276713	-2.634943
C	4.554307	-0.306201	2.497579
H	4.451014	3.777241	2.605864
H	-4.478285	-3.808333	-2.726886
H	4.946707	3.970913	0.922180
H	-4.971182	-3.993424	-1.041383
H	2.245945	3.327542	3.163057
H	-2.274588	-3.360621	-3.290217
H	-0.101027	3.183569	2.562645
H	0.073879	-3.217205	-2.695450
H	1.114518	3.692757	-1.590007
H	-1.133434	-3.710213	1.461615
H	3.407296	3.805302	-0.802726
H	-3.427780	-3.822770	0.679859
H	-2.031829	2.998736	1.971870
H	2.004249	-3.018658	-2.109431

H	-4.289987	2.627963	1.160733
H	4.264346	-2.647233	-1.303680
H	-3.118154	3.077984	-2.805000
H	3.105854	-3.115358	2.663776
H	-0.804344	3.458400	-2.183024
H	0.789831	-3.495157	2.047342
H	-5.349585	2.840537	-2.238043
H	5.334073	-2.867089	2.091265
H	-5.851538	2.651201	-0.554758
H	5.830641	-2.671491	0.407193
H	-5.822340	0.420675	0.520883
H	5.796481	-0.437574	-0.660613
H	-5.501441	-2.014850	0.339412
H	5.474159	1.996735	-0.469746
H	-4.009402	-1.516879	-3.662369
H	3.987646	1.483152	3.532232
H	-4.331219	0.911567	-3.482086
H	4.311577	-0.944341	3.342667
N	-1.569183	-0.444466	3.055357
C	-0.233293	-0.263912	2.987941
H	0.301549	-0.261158	3.928691
C	0.399634	-0.090685	1.780797
H	1.472031	0.050201	1.806638
C	-0.338897	-0.100408	0.585146
C	-1.722934	-0.289096	0.696810
H	-2.383211	-0.308388	-0.160734
C	-2.308365	-0.456691	1.931486
H	-3.374028	-0.603695	2.046080
C	-2.201861	-0.609520	4.376407
H	-1.724082	-1.441163	4.893581
H	-3.260100	-0.819805	4.239162
N	1.540790	0.417670	-3.199540

C	0.208656	0.230105	-3.132405
H	-0.333117	0.218807	-4.068481
C	-0.423994	0.059904	-1.921699
H	-1.495632	-0.085715	-1.948341
C	0.311978	0.078025	-0.727472
C	1.697380	0.273827	-0.838973
H	2.356445	0.300707	0.019140
C	2.280178	0.439747	-2.072208
H	3.344847	0.592680	-2.194221
C	2.216681	0.591966	-4.498145
H	1.471001	0.586713	-5.289758
H	2.743896	1.545814	-4.494563
H	-2.076351	0.313829	4.942144
H	2.917923	-0.230089	-4.642931

$BIPY^{2+}$-$CBPQT^{2(\bullet+)}$(S)

Atom	X	Y	Z
N	2.957162	3.588185	1.378170
N	-2.889617	-3.507919	-1.339601
N	-3.670491	2.858181	-0.940687
N	3.736971	-2.779959	0.983059
C	4.350635	3.418182	1.833756
C	-4.283047	-3.339007	-1.795768
C	1.930787	3.450887	2.262048
C	-1.862980	-3.367942	-2.222768
C	0.636101	3.361238	1.845476
C	-0.568516	-3.278181	-1.805466
C	0.302799	3.390528	0.459472
C	-0.235764	-3.310077	-0.419424
C	1.406267	3.571464	-0.425070
C	-1.339559	-3.492486	0.464405

C	2.682122	3.654379	0.046417
C	-2.615155	-3.575781	-0.007827
C	-1.039931	3.240836	-0.011243
C	1.106818	-3.161148	0.052083
C	-2.139408	3.029935	0.871554
C	2.207232	-2.952830	-0.830191
C	-3.401046	2.837891	0.393843
C	3.468673	-2.761428	-0.351704
C	-2.655431	3.085042	-1.819488
C	2.721105	-3.005151	1.861361
C	-1.374628	3.281197	-1.396619
C	1.440466	-3.200707	1.437746
C	-4.986882	2.407797	-1.433581
C	5.053082	-2.329286	1.476492
C	-4.969069	0.904741	-1.588466
C	5.035175	-0.826071	1.630039
C	-5.316513	0.083115	-0.515435
C	5.383209	-0.005429	0.556427
C	-5.149716	-1.294844	-0.603491
C	5.216549	1.372615	0.643210
C	-4.633271	-1.869384	-1.765366
C	4.699685	1.948252	1.804348
C	-4.330466	-1.051917	-2.855814
C	4.396042	1.131773	2.895274
C	-4.497681	0.325604	-2.767974
C	4.563027	-0.245865	2.808704
H	4.427660	3.837851	2.838050
H	-4.359218	-3.758020	-2.800398
H	4.992244	4.003254	1.172683
H	-4.924399	-3.925150	-1.135401
H	2.200565	3.415331	3.309777
H	-2.132280	-3.330364	-3.270539

H	-0.119938	3.267592	2.613434
H	0.187700	-3.181931	-2.572930
H	1.273421	3.646259	-1.495713
H	-1.207090	-3.569302	1.534944
H	3.531772	3.772724	-0.613644
H	-3.464981	-3.696231	0.651629
H	-2.013752	3.009940	1.945540
H	2.082485	-2.934443	-1.904307
H	-4.242525	2.659069	1.050667
H	4.310870	-2.584360	-1.008082
H	-2.922449	3.099764	-2.868514
H	2.987313	-3.018706	2.910600
H	-0.630393	3.467015	-2.159250
H	0.695382	-3.384493	2.200043
H	-5.178533	2.913092	-2.381448
H	5.244068	-2.833806	2.424902
H	-5.741614	2.735257	-0.716410
H	5.808263	-2.657377	0.760080
H	-5.714228	0.516658	0.397662
H	5.781339	-0.439825	-0.356084
H	-5.417360	-1.921283	0.242826
H	5.484739	1.998327	-0.203470
H	-3.952501	-1.486863	-3.776856
H	4.017633	1.567532	3.815754
H	-4.248951	0.949486	-3.622180
H	4.313738	-0.868950	3.663330
N	-0.958511	-0.273116	3.379609
C	0.343535	-0.089824	3.073928
H	1.035750	-0.043986	3.904901
C	0.755171	0.031145	1.769501
H	1.814075	0.175924	1.604335
C	-0.176106	-0.031383	0.718495

C	-1.519756	-0.228666	1.076350
H	-2.316394	-0.292615	0.347604
C	-1.880195	-0.344990	2.399096
H	-2.908218	-0.495422	2.700220
C	-1.348407	-0.386947	4.795900
H	-0.842649	-1.247689	5.233626
H	-2.425893	-0.520998	4.860315
N	1.019127	0.379971	-3.346036
C	-0.279992	0.187423	-3.043256
H	-0.976282	0.148685	-3.870565
C	-0.690726	0.050614	-1.737914
H	-1.749881	-0.094248	-1.574293
C	0.239022	0.105492	-0.687270
C	1.584697	0.302663	-1.043865
H	2.381509	0.358693	-0.314666
C	1.942942	0.434891	-2.363571
H	2.969918	0.586613	-2.668856
C	1.448033	0.488402	-4.751267
H	0.593491	0.773065	-5.361949
H	2.220718	1.252174	-4.826969
H	-1.059205	0.527161	5.314828
H	1.837856	-0.476815	-5.077572

$BIPY^{2+}$-$CBPQT^{\bullet +}$

Atom	X	Y	Z
N	-3.508138	3.176399	-0.245535
N	3.513238	-3.241280	0.107111
N	3.536608	3.136305	0.397626
N	-3.544576	-3.210541	-0.243813
C	-4.947147	2.895998	-0.372316
C	4.955180	-2.951069	0.192677

C	-2.709526	3.181079	-1.355102
C	2.762761	-3.304327	1.245407
C	-1.353514	3.181225	-1.264018
C	1.403361	-3.326911	1.206958
C	-0.691114	3.141272	0.008717
C	0.694687	-3.276658	-0.032979
C	-1.569970	3.152312	1.137978
C	1.522292	-3.223850	-1.196609
C	-2.921543	3.156838	0.986491
C	2.878830	-3.197000	-1.099928
C	0.719563	3.133436	0.140803
C	-0.726420	-3.272351	-0.103164
C	1.599205	3.208728	-0.985341
C	-1.555407	-3.288813	1.060692
C	2.950983	3.195741	-0.833250
C	-2.911799	-3.246571	0.964356
C	2.737023	3.077981	1.505303
C	-2.792710	-3.211112	-1.383090
C	1.381354	3.090932	1.413766
C	-1.433771	-3.244244	-1.344600
C	4.973279	2.837816	0.509665
C	-4.983403	-2.901904	-0.315828
C	5.170118	1.340747	0.446511
C	-5.167317	-1.402470	-0.347967
C	5.357042	0.701183	-0.779300
C	-5.359994	-0.684884	0.832116
C	5.346765	-0.688507	-0.856726
C	-5.357930	0.707081	0.816871
C	5.153953	-1.456584	0.291464
C	-5.157252	1.399489	-0.377578
C	5.025784	-0.817035	1.525744
C	-5.018569	0.679912	-1.566010

C	5.031630	0.569842	1.602370
C	-5.026482	-0.709026	-1.551521
H	-5.301079	3.359773	-1.295053
H	5.349622	-3.472260	1.066989
H	-5.459012	3.375302	0.464633
H	5.434713	-3.368121	-0.694733
H	-3.220787	3.199709	-2.309506
H	3.311442	-3.338211	2.177989
H	-0.800135	3.219598	-2.192206
H	0.888134	-3.387898	2.156089
H	-1.189945	3.162056	2.150874
H	1.100721	-3.183785	-2.191918
H	-3.594214	3.155280	1.835166
H	3.517062	-3.133485	-1.972208
H	1.219741	3.285182	-1.995669
H	-1.134783	-3.317683	2.056909
H	3.624404	3.242436	-1.680061
H	-3.551310	-3.233048	1.838005
H	3.247496	3.035915	2.459375
H	-3.340421	-3.186430	-2.316523
H	0.827358	3.074932	2.342272
H	-0.917680	-3.251802	-2.295138
H	5.332461	3.256104	1.452023
H	-5.383482	-3.379520	-1.212302
H	5.488644	3.350133	-0.305258
H	-5.467240	-3.352547	0.552591
H	5.500116	1.286333	-1.683768
H	-5.501597	-1.210825	1.772378
H	5.478847	-1.173816	-1.819906
H	-5.502260	1.252167	1.745735
H	4.910000	-1.400438	2.434802
H	-4.894047	1.203205	-2.509799

H	4.916832	1.052316	2.568753
H	-4.911449	-1.251939	-2.485327
N	1.097386	0.213776	-3.363391
C	-0.232434	0.202536	-3.074785
H	-0.905582	0.207149	-3.922424
C	-0.685349	0.173709	-1.788500
H	-1.756473	0.131713	-1.645251
C	0.223481	0.173160	-0.688839
C	1.601126	0.168958	-1.040089
H	2.384698	0.138907	-0.293413
C	1.996723	0.198376	-2.346356
H	3.041681	0.200437	-2.629491
C	1.560856	0.321962	-4.748408
H	0.800856	-0.083817	-5.415185
H	2.481223	-0.250215	-4.865744
N	-1.094278	-0.004995	3.343930
C	0.235463	-0.002447	3.054859
H	0.908677	-0.060606	3.900456
C	0.688221	0.060635	1.769799
H	1.759709	0.036275	1.625008
C	-0.220468	0.132076	0.672438
C	-1.598151	0.116992	1.023380
H	-2.382384	0.153682	0.277508
C	-1.993286	0.053560	2.328724
H	-3.038036	0.035521	2.611600
C	-1.556848	-0.157830	4.725400
H	-0.802734	0.240317	5.403460
H	-2.484672	0.398772	4.857232
H	1.744629	1.370093	-4.995919
H	-1.727068	-1.214409	4.944353

$BIPY^{2+}$-$CBPQT^{0}$

Atom	X	Y	Z
N	2.935846	3.523318	1.539529
N	-2.836689	-3.559297	-1.475690
N	-3.582851	2.864397	-1.210991
N	3.684367	-2.896270	1.268879
C	4.284297	3.326269	2.075509
C	-4.184954	-3.360370	-2.011207
C	1.842050	3.303377	2.348586
C	-1.742840	-3.341589	-2.285503
C	0.595001	3.140559	1.853066
C	-0.495786	-3.177704	-1.790709
C	0.325806	3.148579	0.428499
C	-0.226041	-3.182500	-0.365959
C	1.516851	3.339409	-0.380335
C	-1.416892	-3.372871	0.443570
C	2.737380	3.501806	0.174468
C	-2.637565	-3.535628	-0.110537
C	-0.946242	3.017184	-0.108361
C	1.046126	-3.049516	0.169652
C	-2.140105	2.844608	0.700492
C	2.238808	-2.874297	-0.640502
C	-3.369835	2.763597	0.148767
C	3.469286	-2.793341	-0.090401
C	-2.476016	2.981441	-2.023924
C	2.578421	-3.013160	2.083252
C	-1.220724	3.073154	-1.531071
C	1.322505	-3.104668	1.592037
C	-4.852886	2.404284	-1.776491
C	4.955325	-2.437272	1.833179
C	-4.851900	0.898424	-1.904497
C	4.954915	-0.931623	1.963454

C	-5.143052	0.104207	-0.792409
C	5.245093	-0.135800	0.852250
C	-4.983421	-1.273886	-0.848378
C	5.084756	1.242109	0.910112
C	-4.530046	-1.889059	-2.017870
C	4.631568	1.855499	2.080631
C	-4.284339	-1.101646	-3.143059
C	4.387616	1.066613	3.205151
C	-4.443992	0.280589	-3.086728
C	4.548079	-0.315460	3.146926
H	4.315112	3.747814	3.083381
H	-4.217037	-3.782939	-3.018625
H	4.979129	3.897742	1.454499
H	-4.880556	-3.929952	-1.389299
H	2.043536	3.288068	3.413695
H	-1.944535	-3.328941	-3.350635
H	-0.204646	3.006622	2.571364
H	0.303735	-3.045745	-2.509488
H	1.457108	3.376029	-1.460647
H	-1.356742	-3.407335	1.523974
H	3.625975	3.651152	-0.427878
H	-3.526059	-3.683574	0.492324
H	-2.079005	2.788443	1.779867
H	2.176328	-2.816378	-1.719682
H	-4.261461	2.640591	0.752508
H	4.359934	-2.668802	-0.695273
H	-2.678306	3.016951	-3.088339
H	2.782104	-3.048874	3.147396
H	-0.419467	3.194544	-2.249842
H	0.522146	-3.226184	2.311814
H	-4.987400	2.886934	-2.747797
H	5.091121	-2.921359	2.803594

H	-5.657306	2.747010	-1.120249
H	5.758707	-2.779221	1.175261
H	-5.494006	0.565659	0.126513
H	5.595495	-0.595914	-0.067545
H	-5.209909	-1.874481	0.028421
H	5.310234	1.843931	0.033903
H	-3.956603	-1.568097	-4.068747
H	4.060419	1.531730	4.131707
H	-4.240387	0.881067	-3.969918
H	4.345703	-0.917201	4.029561
N	-0.289006	-0.215466	3.538928
C	-1.383657	-0.309713	2.737489
H	-2.332170	-0.447030	3.240770
C	-1.279390	-0.234396	1.378782
H	-2.194585	-0.315273	0.808992
C	-0.015608	-0.055438	0.755593
C	1.092239	0.039306	1.624589
H	2.102916	0.179500	1.262486
C	0.929774	-0.043354	2.982811
H	1.768253	0.025742	3.662525
C	-0.451599	-0.323239	4.992215
H	-1.165822	0.427161	5.333937
H	0.510359	-0.151910	5.472341
N	0.398477	0.177145	-3.464258
C	1.491305	0.279055	-2.665099
H	2.441567	0.417502	-3.163717
C	1.386752	0.207528	-1.304427
H	2.302061	0.292087	-0.735466
C	0.124925	0.027289	-0.680845
C	-0.984639	-0.071671	-1.549550
H	-1.994559	-0.213450	-1.185894
C	-0.821943	0.004898	-2.905935

H	-1.656950	-0.069014	-3.590231
C	0.512407	0.275063	-4.922800
H	1.565225	0.275283	-5.199843
H	0.019076	-0.583017	-5.381284
H	-0.812184	-1.322023	5.244825
H	0.044129	1.199881	-5.265068

NDI-CBPQT^{4+}

Atom	X	Y	Z
N	-3.479898	3.181909	0.026061
N	3.483696	-3.016140	-0.947708
N	3.579465	3.135131	-0.027574
N	-3.580992	-2.965916	-0.981182
C	-4.968466	2.979153	-0.030028
C	4.972274	-2.807871	-0.923754
C	-2.805637	3.485321	-1.098872
C	2.810782	-3.271234	0.191403
C	-1.431105	3.607655	-1.097540
C	1.432800	-3.383327	0.201167
C	-0.697297	3.398029	0.076401
C	0.699947	-3.232830	-0.982241
C	-1.432798	3.126107	1.236841
C	1.437193	-3.011625	-2.152956
C	-2.810366	3.023256	1.185299
C	2.811712	-2.900168	-2.108916
C	0.800713	3.389674	0.061213
C	-0.799520	-3.229699	-0.994078
C	1.509257	3.461383	-1.144707
C	-1.555123	-3.478315	0.157605
C	2.882242	3.328040	-1.163139
C	-2.930943	-3.338066	0.138946

C	2.935254	3.108047	1.155811
C	-2.888647	-2.774325	-2.120596
C	1.560536	3.237368	1.227194
C	-1.516253	-2.906758	-2.154107
C	5.063215	2.899300	-0.099005
C	-5.063846	-2.713049	-0.963845
C	5.347227	1.433169	-0.346710
C	-5.344754	-1.238612	-0.762095
C	5.244180	0.907109	-1.636719
C	-5.601772	-0.728481	0.513844
C	5.218025	-0.468737	-1.834736
C	-5.575676	0.645804	0.739762
C	5.296039	-1.340152	-0.746299
C	-5.293419	1.527169	-0.307630
C	5.566489	-0.815607	0.520644
C	-5.204980	1.023479	-1.607200
C	5.591919	0.562895	0.719609
C	-5.232723	-0.348254	-1.832799
H	-5.339292	3.644401	-0.810232
H	5.361536	-3.418273	-0.109197
H	-5.368672	3.318427	0.925225
H	5.356019	-3.206777	-1.863148
H	-3.387551	3.626667	-1.999552
H	3.397301	-3.370633	1.095346
H	-0.967985	3.865679	-2.039802
H	0.965697	-3.570310	1.158644
H	-0.967296	2.967359	2.200247
H	0.974935	-2.910915	-3.125161
H	-3.399167	2.798556	2.065677
H	3.395876	-2.715394	-3.000504
H	1.026383	3.605270	-2.101307
H	-1.107409	-3.772823	1.097217

H	3.443356	3.361874	-2.086873
H	-3.530953	-3.510121	1.022907
H	3.541698	2.968843	2.041094
H	-3.454028	-2.503951	-3.002102
H	1.115814	3.202277	2.212662
H	-1.038380	-2.726428	-3.106804
H	5.484083	3.250713	0.842755
H	-5.451743	-3.084934	-1.912803
H	5.437488	3.537530	-0.900039
H	-5.476840	-3.324087	-0.161894
H	5.120679	1.563201	-2.492064
H	-5.763790	-1.402110	1.349956
H	5.072121	-0.852560	-2.839025
H	-5.717944	1.022467	1.748313
H	5.698383	-1.478943	1.370309
H	-5.048904	1.691672	-2.447605
H	5.743596	0.953319	1.721579
H	-5.101561	-0.715711	-2.845235
C	-1.222417	0.091658	-0.475942
C	-0.000423	-0.032275	0.209321
C	-2.401029	-0.051104	0.211379
C	-1.234112	0.384545	-1.931594
C	0.000449	-0.288612	1.596767
C	1.220464	0.093899	-0.477520
C	-1.224111	-0.431633	2.280053
C	1.225199	-0.397632	2.286149
C	-2.408605	-0.316669	1.593930
C	-1.230451	-0.708215	3.742349
C	1.236399	0.350244	-1.940903
C	2.399512	-0.022315	0.213921
C	1.241674	-0.667396	3.751642
C	2.408169	-0.260629	1.601387

C	-0.030344	-1.078595	5.815603
C	-0.023448	0.815548	-3.996903
H	3.321224	0.075064	-0.333511
H	3.344695	-0.346679	2.139165
H	-0.570670	-0.279733	6.325999
H	-3.323042	0.050291	-0.334596
H	-3.344391	-0.428800	2.128533
H	0.975175	1.120875	-4.297963
H	-0.742632	1.609276	-4.194838
H	-0.317634	-0.083349	-4.543033
H	0.994408	-1.126378	6.173778
H	-0.536477	-2.028104	5.996575
N	0.003990	-0.807162	4.377634
N	0.000660	0.541564	-2.557642
O	2.286586	-0.762677	4.365608
O	2.274350	0.407544	-2.574370
O	-2.264823	-0.844202	4.366954
O	-2.266486	0.489027	-2.568223

NDI-CBPQT$^{•3+}$

Atom	X	Y	Z
N	-3.486065	3.179337	0.022227
N	3.476600	-3.018895	-0.949106
N	3.609802	3.133673	-0.013389
N	-3.587700	-2.956639	-0.971081
C	-4.954170	2.979392	-0.026834
C	4.966848	-2.819666	-0.928416
C	-2.782660	3.411531	-1.124641
C	2.804853	-3.266874	0.191909
C	-1.424287	3.517118	-1.131760
C	1.426598	-3.370570	0.205187

C	-0.650098	3.368632	0.062906
C	0.692303	-3.216329	-0.976629
C	-1.432636	3.168755	1.245163
C	1.427556	-2.998344	-2.148861
C	-2.792550	3.074013	1.195973
C	2.802684	-2.895948	-2.108029
C	0.777979	3.364158	0.055549
C	-0.806232	-3.209172	-0.985618
C	1.540805	3.486505	-1.149602
C	-1.560137	-3.462926	0.166172
C	2.897094	3.358804	-1.156324
C	-2.935955	-3.327052	0.148425
C	2.928789	3.064680	1.170447
C	-2.896235	-2.752876	-2.108190
C	1.571275	3.180850	1.233131
C	-1.523464	-2.879444	-2.142764
C	5.069445	2.884422	-0.077207
C	-5.072245	-2.711478	-0.953222
C	5.360047	1.417918	-0.331088
C	-5.358794	-1.239746	-0.751472
C	5.255262	0.895803	-1.622637
C	-5.601752	-0.727807	0.526215
C	5.224053	-0.478652	-1.828032
C	-5.575197	0.647052	0.748484
C	5.299002	-1.356104	-0.743968
C	-5.302008	1.529141	-0.300448
C	5.565321	-0.838167	0.526245
C	-5.227206	1.021314	-1.599407
C	5.597747	0.539668	0.729732
C	-5.256785	-0.350395	-1.823938
H	-5.342367	3.638853	-0.805781
H	5.353692	-3.437844	-0.118326

H	-5.362417	3.315252	0.927755
H	5.343850	-3.216808	-1.871374
H	-3.364620	3.508565	-2.031847
H	3.393367	-3.367124	1.094370
H	-0.960425	3.711577	-2.089705
H	0.959946	-3.551853	1.163885
H	-0.971607	3.063807	2.218840
H	0.962793	-2.891578	-3.119187
H	-3.385122	2.901293	2.085935
H	3.386171	-2.711587	-3.000029
H	1.068472	3.667726	-2.106090
H	-1.110362	-3.757241	1.104791
H	3.470067	3.428853	-2.071748
H	-3.535725	-3.502280	1.031886
H	3.529732	2.902217	2.056574
H	-3.462510	-2.473415	-2.986101
H	1.120038	3.109085	2.214335
H	-1.045658	-2.683189	-3.092421
H	5.501315	3.221366	0.866550
H	-5.457072	-3.086127	-1.902493
H	5.469802	3.516302	-0.872505
H	-5.480522	-3.325711	-0.151114
H	5.125961	1.559110	-2.471846
H	-5.750939	-1.401375	1.365164
H	5.074463	-0.858379	-2.833653
H	-5.703119	1.026280	1.758332
H	5.687818	-1.506605	1.373763
H	-5.074742	1.690178	-2.440308
H	5.743625	0.926909	1.734120
H	-5.132704	-0.720623	-2.836555
C	-1.224591	0.105247	-0.482557
C	-0.002511	-0.019258	0.202693

C	-2.403866	-0.036546	0.202617
C	-1.237816	0.381745	-1.941261
C	-0.002144	-0.275476	1.589223
C	1.219191	0.104379	-0.483238
C	-1.227815	-0.415270	2.271799
C	1.223550	-0.398260	2.275762
C	-2.411736	-0.297806	1.585292
C	-1.234689	-0.690039	3.732021
C	1.231685	0.362898	-1.944778
C	2.398409	-0.022440	0.203997
C	1.239568	-0.669810	3.738495
C	2.406432	-0.269086	1.589532
C	-0.034810	-1.066242	5.804382
C	-0.000732	0.867495	-3.989546
H	3.319292	0.082429	-0.342785
H	3.343409	-0.360870	2.125349
H	-0.570635	-0.263954	6.314409
H	-3.325056	0.067124	-0.343924
H	-3.348241	-0.404577	2.119290
H	0.893104	1.440196	-4.227824
H	-0.895568	1.437291	-4.230228
H	0.002344	-0.065303	-4.559623
H	0.989767	-1.119204	6.162418
H	-0.546359	-2.012415	5.988500
N	0.000463	-0.797497	4.366673
N	-0.002541	0.586152	-2.553785
O	2.282798	-0.777200	4.355736
O	2.259089	0.378592	-2.597804
O	-2.267666	-0.819135	4.362749
O	-2.267646	0.418032	-2.590289

NDI-CBPQT$^{2(\bullet+)}$(T)

Atom	X	Y	Z
N	-3.556990	3.092861	-0.224739
N	3.557941	-3.092175	0.232225
N	3.549770	3.097964	0.222930
N	-3.548776	-3.096546	-0.216096
C	-5.025205	2.877080	-0.325886
C	5.026101	-2.876099	0.333464
C	-2.785806	3.213725	-1.347083
C	2.786651	-3.212708	1.354551
C	-1.427187	3.325864	-1.288897
C	1.428050	-3.324883	1.296248
C	-0.718524	3.316208	-0.044611
C	0.719503	-3.315466	0.051868
C	-1.575456	3.257823	1.103395
C	1.576563	-3.257897	-1.096059
C	-2.930462	3.137874	0.989181
C	2.931577	-3.138090	-0.981742
C	0.711005	3.317148	0.042497
C	-0.710020	-3.315904	-0.035375
C	1.567900	3.257109	-1.105396
C	-1.567054	-3.255649	1.112450
C	2.923088	3.139407	-0.991015
C	-2.922220	-3.137843	0.997921
C	2.778527	3.220515	1.345069
C	-2.777378	-3.218748	-1.338138
C	1.419759	3.330700	1.286720
C	-1.418613	-3.329039	-1.279662
C	5.018437	2.885479	0.324403
C	-5.017487	-2.884328	-0.317709
C	5.381344	1.411810	0.376574
C	-5.380667	-1.410722	-0.369025

C	5.668275	0.701434	-0.790786
C	-5.662112	-0.700504	0.799759
C	5.670291	-0.693976	-0.788547
C	-5.664133	0.694829	0.797740
C	5.385367	-1.401400	0.381087
C	-5.384742	1.402453	-0.373098
C	5.301517	-0.688758	1.582597
C	-5.305951	0.690058	-1.575149
C	5.299553	0.702786	1.580356
C	-5.303895	-0.701576	-1.573120
H	-5.361016	3.398739	-1.224205
H	5.361904	-3.397807	1.231774
H	-5.482090	3.367303	0.535868
H	5.483195	-3.366104	-0.528292
H	-3.314581	3.206628	-2.292023
H	3.315320	-3.205511	2.299544
H	-0.908457	3.412982	-2.235105
H	0.909231	-3.411963	2.242414
H	-1.172898	3.275644	2.108353
H	1.174067	-3.276025	-2.101018
H	-3.569798	3.054128	1.859029
H	3.571066	-3.054937	-1.851534
H	1.165183	3.271757	-2.110332
H	-1.164533	-3.270570	2.117468
H	3.562498	3.054549	-1.860687
H	-3.561707	-3.053128	1.867557
H	3.307376	3.216772	2.289984
H	-3.306113	-3.214528	-2.283121
H	0.900972	3.419648	2.232734
H	-0.899717	-3.417555	-2.225654
H	5.353270	3.410827	1.220954
H	-5.352118	-3.409297	-1.214541

H	5.474130	3.373881	-0.539005
H	-5.473139	-3.373279	0.545419
H	5.793827	1.226838	-1.732210
H	-5.783492	-1.226063	1.741641
H	5.797474	-1.222079	-1.728255
H	-5.786808	1.222767	1.738132
H	5.161290	-1.217882	2.521315
H	-5.169435	1.219399	-2.514294
H	5.157931	1.234457	2.517425
H	-5.165863	-1.233175	-2.510764
C	-0.742009	-0.001673	-1.715028
C	0.208710	-0.000815	-0.679720
C	-2.077831	-0.000744	-1.407821
C	-0.306400	-0.003696	-3.129200
C	-0.206688	0.001222	0.667409
C	1.579454	-0.001333	-0.971630
C	-1.577613	0.001249	0.957626
C	0.742726	0.002943	1.703932
C	-2.488007	0.000226	-0.067521
C	-2.038384	0.002593	2.366284
C	2.048168	-0.005354	-2.379699
C	2.488803	0.001046	0.054890
C	0.314179	0.004707	3.122174
C	2.078303	0.003082	1.394982
C	-1.539446	0.005643	4.739248
C	1.478510	-0.023202	-4.771913
H	3.529676	0.000672	-0.199473
H	2.806690	0.004548	2.194552
H	-2.146975	0.894546	4.917883
H	-2.805608	-0.001101	-2.208203
H	-3.528297	0.000567	0.188772
H	2.562626	-0.090172	-4.802944

H	1.145331	0.893650	-5.261963
H	1.032480	-0.880562	-5.278035
H	-0.672073	0.006871	5.394113
H	-2.145974	-0.883533	4.919948
N	-1.061083	0.004305	3.358044
N	1.065658	-0.014737	-3.369613
O	1.116275	0.006310	4.039581
O	3.232422	-0.002101	-2.668163
O	-3.218326	0.002290	2.673165
O	-1.094736	0.002098	-4.058843

NDI-CBPQT$^{2(\bullet+)}$(S)

Atom	X	Y	Z
N	3.546932	3.121887	-0.439177
N	-3.545316	-3.108030	-0.521660
N	-3.550796	3.115674	-0.448307
N	3.552687	-3.109584	-0.522467
C	5.011907	2.891777	-0.454546
C	-5.010891	-2.880449	-0.534061
C	2.854758	3.212775	0.736224
C	-2.837369	-3.110303	-1.689022
C	1.495892	3.321698	0.770776
C	-1.479812	-3.220288	-1.712469
C	0.711873	3.328091	-0.426890
C	-0.710163	-3.315134	-0.509418
C	1.485143	3.283959	-1.630612
C	-1.497342	-3.350975	0.685705
C	2.842606	3.172126	-1.607783
C	-2.856284	-3.240999	0.651518
C	-0.715934	3.325185	-0.429388
C	0.717703	-3.317452	-0.509189

C	-1.504261	3.336283	0.765473
C	1.488318	-3.241116	-1.713034
C	-2.862991	3.225809	0.727931
C	2.845729	-3.129446	-1.690415
C	-2.841871	3.142838	-1.614788
C	2.863112	-3.231026	0.651741
C	-1.484372	3.255033	-1.634858
C	1.504311	-3.341599	0.686542
C	-5.016124	2.886628	-0.466168
C	5.017232	-2.876685	-0.535369
C	-5.340606	1.407180	-0.516788
C	5.338659	-1.395687	-0.549648
C	-5.588199	0.685551	0.653768
C	5.251744	-0.669814	-1.739596
C	-5.586753	-0.707773	0.636931
C	5.250447	0.720514	-1.719679
C	-5.337679	-1.400703	-0.550454
C	5.335866	1.412313	-0.509514
C	-5.259252	-0.677509	-1.742584
C	5.589524	0.688440	0.658335
C	-5.260777	0.712896	-1.725881
C	5.590997	-0.704904	0.638267
H	5.424082	3.357673	0.442232
H	-5.407921	-3.387866	-1.415715
H	5.411780	3.421365	-1.321720
H	-5.423586	-3.370297	0.349556
H	3.447986	3.184330	1.641605
H	-3.415482	-3.017299	-2.599030
H	1.036086	3.380147	1.748835
H	-1.011285	-3.211878	-2.687866
H	1.020008	3.329833	-2.606523
H	-1.040131	-3.448147	1.661954

H	3.424029	3.119357	-2.518969
H	-3.451953	-3.245765	1.555756
H	-1.048097	3.416114	1.743740
H	1.020754	-3.258707	-2.688708
H	-3.459532	3.212830	1.631485
H	3.425013	-3.052643	-2.601278
H	-3.419125	3.067080	-2.526924
H	3.458331	-3.225017	1.556269
H	-1.015014	3.266594	-2.609846
H	1.046597	-3.425870	1.663730
H	-5.413708	3.413430	-1.336075
H	5.432549	-3.366186	0.347261
H	-5.429528	3.355935	0.428221
H	5.415634	-3.381314	-1.418010
H	-5.717787	1.207387	1.597893
H	5.110401	-1.184222	-2.684918
H	-5.715198	-1.252400	1.568300
H	5.108384	1.261013	-2.650373
H	-5.122791	-1.193917	-2.687595
H	5.722393	1.208220	1.603134
H	-5.125646	1.251799	-2.658574
H	5.724941	-1.251276	1.567836
C	1.222133	-0.031960	2.304792
C	-0.003525	-0.020872	1.606793
C	2.407033	-0.023933	1.610129
C	1.226496	-0.053250	3.788087
C	-0.000804	-0.000159	0.197798
C	-1.231226	-0.031118	2.301188
C	1.223048	0.009331	-0.494764
C	-1.221239	0.009121	-0.501004
C	2.401575	-0.003529	0.203961
C	1.237631	0.033395	-1.976903

C	-1.249820	-0.053012	3.786133
C	-2.412774	-0.021903	1.600466
C	-1.238283	0.031179	-1.985077
C	-2.401972	-0.002087	0.194474
C	0.041052	0.083319	-4.083257
C	0.020186	-0.082838	5.891791
H	-3.351266	-0.030501	2.140979
H	-3.319655	0.004584	-0.367019
H	0.618004	0.947205	-4.417012
H	3.343160	-0.033524	2.154961
H	3.321000	0.002683	-0.354431
H	-1.005990	-0.092393	6.249311
H	0.538399	0.803271	6.262520
H	0.546387	-0.974352	6.237257
H	-0.981131	0.150617	-4.445350
H	0.513610	-0.826271	-4.459329
N	0.002945	0.055617	-2.622421
N	-0.010812	-0.062418	4.429977
O	-2.274170	0.031179	-2.625170
O	-2.292555	-0.062229	4.415900
O	2.265892	0.036332	-2.629620
O	2.256820	-0.062427	4.438496

NDI-CBPQT$^{\bullet+}$

Atom	X	Y	Z
N	-4.531979	2.720516	-0.095134
N	4.418842	-2.450236	-1.050093
N	2.574629	3.380184	0.122045
N	-2.616754	-3.301528	-0.975421
C	-5.838649	2.063862	-0.231622
C	5.772381	-1.848613	-0.980320

C	-3.813908	3.090738	-1.219726
C	3.801710	-2.940709	0.067223
C	-2.487590	3.342448	-1.189732
C	2.474612	-3.252656	0.081634
C	-1.693376	3.168678	0.015448
C	1.643764	-3.045054	-1.065414
C	-2.504198	2.868072	1.178675
C	2.359141	-2.651169	-2.239161
C	-3.834321	2.627930	1.090088
C	3.685072	-2.347732	-2.197944
C	-0.302739	3.248377	0.052093
C	0.220270	-3.149891	-1.033489
C	0.523307	3.461737	-1.118125
C	-0.521808	-3.378559	0.168821
C	1.877095	3.516120	-1.055352
C	-1.884273	-3.438708	0.169194
C	1.838733	3.158135	1.265300
C	-1.951888	-3.056801	-2.141938
C	0.486470	3.085935	1.262164
C	-0.592721	-2.976025	-2.198030
C	4.039479	3.534008	0.155586
C	-4.094808	-3.410332	-0.959965
C	4.787942	2.250639	-0.153607
C	-4.783146	-2.070758	-0.806941
C	4.917235	1.810653	-1.472131
C	-5.254122	-1.638875	0.435235
C	5.352041	0.519520	-1.747926
C	-5.699205	-0.329809	0.605713
C	5.669255	-0.359776	-0.709661
C	-5.677609	0.572384	-0.461658
C	5.704255	0.136136	0.596263
C	-5.331131	0.097190	-1.728030

C	5.268584	1.430833	0.869986
C	-4.891030	-1.208932	-1.899588
H	-6.362285	2.538629	-1.065837
H	6.321261	-2.366375	-0.191504
H	-6.411360	2.261209	0.678800
H	6.269115	-2.054432	-1.931066
H	-4.394253	3.188077	-2.129977
H	4.425030	-3.047400	0.946422
H	-2.025902	3.656467	-2.118455
H	2.069448	-3.630294	1.011742
H	-2.056804	2.778307	2.161063
H	1.861605	-2.517747	-3.190544
H	-4.424385	2.357245	1.958833
H	4.214533	-1.990619	-3.071356
H	0.076997	3.574888	-2.099055
H	-0.027776	-3.481864	1.126403
H	2.478554	3.668254	-1.943161
H	-2.448558	-3.599534	1.079091
H	2.414270	3.030362	2.174559
H	-2.565117	-2.941509	-3.026123
H	0.009644	2.892345	2.215091
H	-0.156651	-2.787564	-3.170189
H	4.305709	3.905416	1.148009
H	-4.384133	-3.897738	-1.893855
H	4.301320	4.314097	-0.564738
H	-4.357520	-4.084274	-0.142573
H	4.604898	2.450004	-2.292044
H	-5.223117	-2.309522	1.289925
H	5.367156	0.175240	-2.777680
H	-6.005996	0.006307	1.592945
H	6.002135	-0.513496	1.415215
H	-5.335261	0.771563	-2.578716

H	5.231070	1.774105	1.900466
H	-4.572651	-1.533975	-2.885392
C	-1.217046	0.113599	-0.411071
C	0.025852	0.003914	0.244996
C	-2.388646	-0.064969	0.297652
C	-1.261592	0.378004	-1.854426
C	0.059425	-0.268992	1.631722
C	1.228660	0.167829	-0.460247
C	-1.152832	-0.451646	2.336058
C	1.302725	-0.361905	2.285504
C	-2.359006	-0.346108	1.665733
C	-1.124560	-0.740413	3.773733
C	1.215293	0.433965	-1.913450
C	2.435326	0.034852	0.196890
C	1.357191	-0.642194	3.734233
C	2.476792	-0.222564	1.565296
C	0.135382	-1.099030	5.816669
C	-0.112914	0.795802	-3.943739
H	3.336926	0.111681	-0.384724
H	3.425314	-0.325883	2.077906
H	-0.419403	-0.324403	6.349755
H	-3.322542	0.045494	-0.226101
H	-3.284726	-0.480385	2.213695
H	0.901445	0.904800	-4.318213
H	-0.680741	1.711780	-4.120807
H	-0.614745	-0.031349	-4.450954
H	1.168089	-1.119123	6.154505
H	-0.339560	-2.064079	6.004427
N	0.133209	-0.814683	4.384366
N	-0.039375	0.530076	-2.512263
O	2.414000	-0.724652	4.345863
O	2.237343	0.536697	-2.578623

O	-2.132659	-0.913828	4.447573
O	-2.299966	0.461800	-2.496309

NDI-CBPQT^0

Atom	X	Y	Z
N	2.743949	3.110902	0.808494
N	-2.417330	-3.078093	-1.471319
N	-3.992928	3.144541	-1.595117
N	4.398372	-3.097228	0.701937
C	4.126595	3.101480	1.298728
C	-3.813711	-3.075855	-1.921304
C	1.665823	3.023710	1.668411
C	-1.373486	-2.984353	-2.370765
C	0.384230	3.091855	1.247207
C	-0.074655	-3.044014	-1.999736
C	0.022700	3.259029	-0.159743
C	0.341438	-3.215769	-0.611836
C	1.199576	3.295556	-1.021468
C	-0.803807	-3.257872	0.296609
C	2.460390	3.225233	-0.538359
C	-2.081712	-3.189320	-0.135305
C	-1.276553	3.336674	-0.619095
C	1.656795	-3.292379	-0.201126
C	-2.449513	3.253652	0.246152
C	2.806400	-3.211008	-1.100135
C	-3.702726	3.127531	-0.239880
C	4.069411	-3.083930	-0.648384
C	-2.916836	3.326866	-2.455801
C	3.349581	-3.277448	1.590342
C	-1.645880	3.458489	-2.027923
C	2.065487	-3.411518	1.195357

C	-5.236355	2.543510	-2.088838
C	5.653714	-2.491952	1.158744
C	-5.142876	1.029832	-2.175114
C	5.550059	-0.980622	1.269280
C	-5.577494	0.214846	-1.125867
C	5.617163	-0.192317	0.118259
C	-5.216870	-1.130679	-1.076502
C	5.232212	1.142241	0.146896
C	-4.414871	-1.689931	-2.074323
C	4.766395	1.724897	1.326827
C	-4.106582	-0.909740	-3.190064
C	4.833249	0.977990	2.505543
C	-4.463393	0.432203	-3.238743
C	5.220664	-0.360813	2.477901
H	4.122703	3.538354	2.301123
H	-3.850554	-3.610635	-2.875513
H	4.705881	3.773672	0.658162
H	-4.392277	-3.662508	-1.202435
H	1.915721	2.880527	2.713746
H	-1.660637	-2.856339	-3.407956
H	-0.383469	2.988032	2.004814
H	0.662821	-2.945802	-2.788343
H	1.092151	3.373459	-2.097407
H	-0.654973	-3.321090	1.367961
H	3.321760	3.254395	-1.195667
H	-2.918365	-3.204656	0.554163
H	-2.340941	3.255925	1.324692
H	2.668449	-3.225839	-2.175407
H	-4.561825	3.022909	0.414875
H	4.911718	-2.990987	-1.325609
H	-3.174107	3.384521	-3.508059
H	3.633323	-3.322616	2.636864

H	-0.888574	3.633368	-2.783827
H	1.329423	-3.577305	1.973702
H	-5.444777	2.978859	-3.070960
H	5.906246	-2.936150	2.126231
H	-6.044112	2.844057	-1.414653
H	6.435267	-2.777674	0.447992
H	-6.145614	0.647400	-0.305746
H	5.902905	-0.643024	-0.827475
H	-5.510366	-1.731891	-0.219362
H	5.232622	1.712804	-0.777099
H	-3.522177	-1.331827	-4.002137
H	4.519198	1.424917	3.445588
H	-4.143045	1.036800	-4.082038
H	5.202305	-0.942675	3.396479
C	0.410943	-0.325638	2.757344
C	-0.531547	-0.166687	1.718703
C	1.758010	-0.396378	2.461596
C	-0.051879	-0.440865	4.149929
C	-0.080677	-0.046833	0.388029
C	-1.916649	-0.123947	1.988535
C	1.297465	-0.116588	0.113274
C	-1.013499	0.139825	-0.649028
C	2.197387	-0.300201	1.137429
C	1.779743	0.007756	-1.275661
C	-2.404474	-0.257444	3.372376
C	-2.811996	0.068536	0.954615
C	-0.557674	0.268738	-2.048617
C	-2.357280	0.206333	-0.360643
C	1.321663	0.348139	-3.623498
C	-1.866590	-0.542599	5.755730
H	-3.872527	0.122722	1.171172
H	-3.044580	0.395897	-1.167034

H	2.057678	1.153828	-3.660374
H	2.473218	-0.537651	3.263735
H	3.240590	-0.395318	0.890380
H	-2.952948	-0.571329	5.767250
H	-1.509815	0.307388	6.341937
H	-1.460514	-1.460059	6.185339
H	0.479897	0.575615	-4.272190
H	1.801573	-0.579558	-3.945751
N	0.819474	0.207823	-2.263912
N	-1.432048	-0.412695	4.368024
O	-1.325037	0.428269	-2.985223
O	-3.594070	-0.234274	3.659037
O	2.958507	-0.057080	-1.589432
O	0.707913	-0.562340	5.102014

TTF-$CBPQT^{4+}$

Atom	X	Y	Z
N	2.858286	3.616121	1.400831
N	-2.875815	-3.593626	-1.372853
N	-3.626561	2.944991	-1.124258
N	3.615119	-2.921729	1.139150
C	4.247498	3.368688	1.880848
C	-4.265494	-3.346172	-1.851310
C	1.826969	3.379146	2.230711
C	-1.845598	-3.364302	-2.206201
C	0.528482	3.394806	1.770145
C	-0.546201	-3.380456	-1.748201
C	0.267282	3.644203	0.420570
C	-0.282383	-3.621942	-0.397601
C	1.357360	3.956272	-0.402007
C	-1.371890	-3.926041	0.428956

C	2.635523	3.927782	0.108564
C	-2.650967	-3.897764	-0.079142
C	-1.102461	3.499648	-0.127515
C	1.088769	-3.477356	0.147123
C	-2.229664	3.512953	0.703809
C	2.214017	-3.485358	-0.687161
C	-3.471935	3.226617	0.184650
C	3.457193	-3.198807	-0.170369
C	-2.572508	2.984522	-1.958785
C	2.563368	-2.966214	1.976275
C	-1.307828	3.265381	-1.489420
C	1.297969	-3.247774	1.509315
C	-4.929988	2.427539	-1.628467
C	4.918893	-2.403633	1.641633
C	-4.866552	0.919120	-1.710016
C	4.853632	-0.895490	1.727268
C	-4.921343	0.156546	-0.540690
C	4.905841	-0.129752	0.559882
C	-4.763604	-1.220727	-0.594149
C	4.746322	1.247168	0.617143
C	-4.547109	-1.860286	-1.817365
C	4.530870	1.883233	1.842364
C	-4.526318	-1.104482	-2.987891
C	4.512736	1.124333	3.010941
C	-4.685083	0.279590	-2.934287
C	4.673141	-0.259398	2.953472
H	4.323756	3.775958	2.889375
H	-4.343963	-3.756851	-2.858268
H	4.916965	3.934668	1.232304
H	-4.934567	-3.909118	-1.199693
H	2.069098	3.147754	3.259654
H	-2.089084	-3.138757	-3.236120

H	-0.251156	3.141669	2.476861
H	0.232296	-3.135103	-2.458769
H	1.238922	4.221311	-1.444222
H	-1.252715	-4.184912	1.472604
H	3.503537	4.145925	-0.498865
H	-3.518152	-4.110153	0.531446
H	-2.167653	3.739909	1.759637
H	2.150137	-3.708084	-1.743725
H	-4.362598	3.207186	0.798113
H	4.346090	-3.175632	-0.786263
H	-2.762793	2.755534	-2.999250
H	2.755812	-2.740333	3.017014
H	-0.494177	3.231765	-2.202263
H	0.486701	-3.218374	2.224922
H	-5.111607	2.885954	-2.600783
H	5.103292	-2.864227	2.612403
H	-5.700711	2.771929	-0.938412
H	5.688522	-2.745115	0.948911
H	-5.083130	0.634294	0.421235
H	5.066951	-0.604889	-0.403440
H	-4.807390	-1.792797	0.327983
H	4.787752	1.821689	-0.303573
H	-4.380335	-1.587124	-3.949544
H	4.367492	1.604323	3.974030
H	-4.658399	0.855477	-3.854723
H	4.648493	-0.837750	3.872407
C	-0.084235	-0.013145	0.678997
C	0.291475	0.012913	3.245395
C	-1.025881	-0.127396	3.091561
H	0.802439	0.069935	4.198406
H	-1.737607	-0.201257	3.904289
C	0.073389	0.028455	-0.663689

C	1.013137	0.163815	-3.075975
C	-0.304069	0.022369	-3.230052
H	1.723890	0.248205	-3.888533
H	-0.815997	-0.024676	-4.183083
S	-1.662953	-0.201660	1.458535
S	1.275980	0.112092	1.797684
S	-1.286650	-0.096840	-1.782512
S	1.652019	0.218567	-1.442920

TTF-CBPQT$^{\bullet 3+}$

Atom	X	Y	Z
N	2.852178	3.617337	1.490154
N	-2.882912	-3.497499	-1.333570
N	-3.818561	2.951464	-0.674217
N	3.532791	-2.899124	1.341827
C	4.230382	3.367470	1.944532
C	-4.277980	-3.278353	-1.814840
C	1.831888	3.732973	2.389095
C	-1.855761	-3.106226	-2.109985
C	0.528930	3.743715	1.995990
C	-0.562576	-3.132592	-1.637878
C	0.166760	3.605416	0.620092
C	-0.308769	-3.550233	-0.329934
C	1.269257	3.542721	-0.287088
C	-1.389385	-4.002936	0.433197
C	2.552866	3.534564	0.160507
C	-2.663362	-3.959889	-0.088708
C	-1.181040	3.473363	0.185651
C	1.046536	-3.419045	0.253132
C	-2.280384	3.374731	1.093506
C	2.189059	-3.376349	-0.550176

C	-3.539410	3.103446	0.652944
C	3.415620	-3.105384	0.017120
C	-2.809566	3.104274	-1.581528
C	2.457722	-2.984765	2.146733
C	-1.532816	3.367000	-1.196154
C	1.208888	-3.245139	1.630539
C	-5.121174	2.427808	-1.118753
C	4.825572	-2.412280	1.905271
C	-5.037256	0.932218	-1.330107
C	4.795687	-0.904101	1.966872
C	-5.217215	0.054992	-0.258225
C	5.144986	-0.153419	0.844306
C	-5.014340	-1.309078	-0.424487
C	5.004544	1.229299	0.855834
C	-4.627329	-1.817314	-1.666734
C	4.507597	1.880796	1.985177
C	-4.487722	-0.948516	-2.747679
C	4.195096	1.131881	3.120214
C	-4.693411	0.417308	-2.579112
C	4.339547	-0.251141	3.112716
H	4.344095	3.825501	2.928669
H	-4.318086	-3.610831	-2.852709
H	4.907627	3.878732	1.257162
H	-4.925319	-3.924226	-1.221530
H	2.119681	3.818068	3.429510
H	-2.099459	-2.744063	-3.099666
H	-0.218331	3.848755	2.771568
H	0.219129	-2.765049	-2.290113
H	1.122944	3.458389	-1.355523
H	-1.265160	-4.390269	1.435966
H	3.395051	3.455877	-0.515118
H	-3.529422	-4.281094	0.474447

H	-2.144329	3.474667	2.162037
H	2.150602	-3.544000	-1.618786
H	-4.374820	2.991588	1.332554
H	4.321619	-3.039856	-0.570198
H	-3.084884	3.000648	-2.623470
H	2.620324	-2.809254	3.201669
H	-0.797476	3.481401	-1.981489
H	0.370716	-3.245879	2.314970
H	-5.392963	2.945141	-2.041346
H	4.943991	-2.869082	2.888308
H	-5.859774	2.686538	-0.358252
H	5.617849	-2.785005	1.256139
H	-5.509004	0.437925	0.715492
H	5.527273	-0.642836	-0.046979
H	-5.151179	-1.976281	0.421730
H	5.281772	1.799919	-0.026038
H	-4.219392	-1.332218	-3.728144
H	3.830488	1.625230	4.016447
H	-4.580295	1.082101	-3.430716
H	4.091873	-0.817952	4.005860
C	0.074905	0.084441	-0.235888
C	-0.262890	-0.122321	2.324225
C	-1.485372	-0.245537	1.805318
H	-0.032558	-0.182326	3.380696
H	-2.387021	-0.415626	2.380587
C	0.596496	0.138353	-1.477098
C	2.163153	-0.223620	-3.502058
C	0.932339	-0.247702	-4.015029
H	3.069385	-0.405379	-4.066391
H	0.700636	-0.451091	-5.053004
S	-1.662823	-0.093652	0.066500
S	1.070742	0.186111	1.223982

S	-0.407071	0.135470	-2.940317
S	2.340214	0.180925	-1.799863

TTF-CBPQT$^{2(\bullet+)}$(T)

Atom	X	Y	Z
N	2.881684	3.600466	1.475485
N	-2.889864	-3.504554	-1.473475
N	-3.653871	2.964099	-1.150350
N	3.645408	-2.868378	1.153032
C	4.249473	3.381475	1.981700
C	-4.257635	-3.285563	-1.979738
C	1.805963	3.512846	2.311265
C	-1.814075	-3.416853	-2.309168
C	0.528254	3.503558	1.840960
C	-0.536394	-3.407748	-1.838777
C	0.247043	3.552604	0.439098
C	-0.255280	-3.457036	-0.436894
C	1.402597	3.693026	-0.391912
C	-1.410906	-3.597523	0.394011
C	2.658087	3.702005	0.132156
C	-2.666360	-3.606333	-0.130149
C	-1.067560	3.424085	-0.092305
C	1.059267	-3.328597	0.094648
C	-2.208329	3.176094	0.733596
C	2.200080	-3.080273	-0.731098
C	-3.437498	2.947642	0.197976
C	3.429159	-2.851710	-0.195315
C	-2.602951	3.242791	-1.976054
C	2.594471	-3.147436	1.978578
C	-1.352542	3.483337	-1.493010
C	1.344150	-3.388139	1.495370
C	-4.937870	2.483314	-1.694802

C	4.929308	-2.387519	1.697666
C	-4.937325	0.974402	-1.822908
C	4.928776	-0.878587	1.825577
C	-5.191852	0.173423	-0.707635
C	5.183569	-0.077798	0.710236
C	-5.028102	-1.204890	-0.775228
C	5.019915	1.300552	0.777568
C	-4.608894	-1.812748	-1.960270
C	4.600585	1.908619	1.962436
C	-4.427773	-1.021679	-3.094861
C	4.419192	1.117722	3.097131
C	-4.589438	0.360958	-3.026227
C	4.580706	-0.264934	3.028747
H	4.299371	3.791270	2.992350
H	-4.307438	-3.695224	-2.990448
H	4.927159	3.962330	1.352302
H	-4.935319	-3.866566	-1.350472
H	2.029185	3.438969	3.368460
H	-2.037233	-3.342868	-3.366369
H	-0.261972	3.430022	2.576481
H	0.253905	-3.334289	-2.574231
H	1.319085	3.775206	-1.467261
H	-1.327472	-3.680111	1.469332
H	3.539163	3.787821	-0.491237
H	-3.547476	-3.692281	0.493168
H	-2.128824	3.136296	1.811558
H	2.120675	-3.040287	-1.809057
H	-4.301260	2.738710	0.816391
H	4.292940	-2.642536	-0.813620
H	-2.820228	3.251557	-3.037089
H	2.811677	-3.156436	3.039624
H	-0.580298	3.695982	-2.220613

H	0.571913	-3.601270	2.222840
H	-5.089289	2.965638	-2.662209
H	5.080549	-2.869734	2.665154
H	-5.727640	2.826599	-1.022740
H	5.719199	-2.730878	1.025786
H	-5.503940	0.627176	0.228772
H	5.495835	-0.531703	-0.226037
H	-5.213068	-1.805969	0.110717
H	5.205073	1.901469	-0.108448
H	-4.134644	-1.480619	-4.035284
H	4.126046	1.576869	4.037452
H	-4.419916	0.963612	-3.914596
H	4.410899	-0.867447	3.917155
C	0.067664	0.018702	0.668942
C	1.009227	-0.002603	3.081827
C	-0.306145	-0.158710	3.232323
H	1.720281	0.034214	3.897347
H	-0.810335	-0.266049	4.184582
C	-0.076217	0.076279	-0.666443
C	0.296843	0.259594	-3.229516
C	-1.018559	0.103795	-3.078974
H	0.800698	0.369731	-4.181635
H	-1.729960	0.069872	-3.894316
S	-1.296622	-0.196063	1.783239
S	1.640509	0.151091	1.455947
S	-1.649133	-0.055760	-1.453367
S	1.288113	0.290375	-1.780818

TTF-CBPQT$^{2(\bullet+)}$(S)

Atom	X	Y	Z
N	2.822133	3.655162	1.504074
N	-3.009665	-3.563422	-1.159982

N	-3.786230	2.969960	-0.837680
N	3.606598	-2.874857	1.171588
C	4.188199	3.405188	1.995291
C	-4.402165	-3.321624	-1.573257
C	1.774887	3.758606	2.372038
C	-1.990196	-3.323118	-2.035580
C	0.484391	3.772986	1.939445
C	-0.690484	-3.309844	-1.636501
C	0.163881	3.644468	0.552252
C	-0.331507	-3.517746	-0.268780
C	1.294421	3.594759	-0.321299
C	-1.426676	-3.819647	0.599275
C	2.563676	3.586124	0.165257
C	-2.709194	-3.818684	0.146596
C	-1.169908	3.505564	0.078904
C	1.002714	-3.376630	0.202115
C	-2.302710	3.488766	0.950619
C	2.084538	-3.006219	-0.655025
C	-3.551039	3.213146	0.483798
C	3.325431	-2.756860	-0.158999
C	-2.741446	3.029737	-1.714583
C	2.614993	-3.271842	2.020992
C	-1.474685	3.302074	-1.302910
C	1.356069	-3.535623	1.578329
C	-5.086928	2.446320	-1.288769
C	4.882911	-2.361745	1.698032
C	-5.039557	0.938527	-1.389574
C	4.825206	-0.856928	1.836432
C	-5.196169	0.150841	-0.246488
C	5.271392	-0.037519	0.801038
C	-5.033685	-1.226827	-0.314577
C	5.104222	1.342230	0.872208

C	-4.712325	-1.842251	-1.526948
C	4.488750	1.923284	1.979707
C	-4.593819	-1.060451	-2.674342
C	4.075993	1.105910	3.034379
C	-4.756335	0.320656	-2.606055
C	4.243170	-0.271668	2.963365
H	4.258356	3.821928	3.001600
H	-4.527575	-3.724154	-2.580721
H	4.879680	3.958497	1.355932
H	-5.048660	-3.891394	-0.902813
H	2.030512	3.828085	3.422095
H	-2.277933	-3.132496	-3.061895
H	-0.285169	3.860233	2.694995
H	0.052301	-3.100128	-2.394699
H	1.181751	3.530686	-1.395211
H	-1.276514	-4.030668	1.649441
H	3.427101	3.517562	-0.484610
H	-3.550268	-4.018961	0.798380
H	-2.204321	3.670851	2.012493
H	1.946484	-2.872447	-1.719594
H	-4.414175	3.172883	1.136500
H	4.144311	-2.441189	-0.792986
H	-2.978990	2.841466	-2.754057
H	2.889285	-3.365300	3.064275
H	-0.707798	3.327684	-2.065381
H	0.631416	-3.847394	2.318382
H	-5.315092	2.903520	-2.253984
H	5.062059	-2.848758	2.658484
H	-5.840789	2.778708	-0.572824
H	5.675646	-2.669118	1.012469
H	-5.434835	0.617051	0.705554
H	5.748065	-0.473887	-0.072406

H	-5.148949	-1.824421	0.585673
H	5.454777	1.966118	0.054532
H	-4.368791	-1.526150	-3.629793
H	3.612554	1.545318	3.913462
H	-4.657475	0.915699	-3.509939
H	3.910217	-0.894613	3.789170
C	0.113292	0.119461	-0.400167
C	-0.017388	-0.396694	2.131205
C	-1.267808	-0.531905	1.687443
H	0.303755	-0.596293	3.145926
H	-2.106191	-0.857603	2.290513
C	0.549690	0.240649	-1.667452
C	2.037468	-0.043933	-3.763160
C	0.787883	-0.181893	-4.207884
H	2.925852	-0.183538	-4.366479
H	0.521318	-0.447549	-5.223378
S	-1.591774	-0.125093	0.010581
S	1.196822	0.175304	0.999661
S	-0.522449	0.153259	-3.080486
S	2.259607	0.461026	-2.091344

TTF-CBPQT$^{•+}$

Atom	X	Y	Z
N	3.004459	3.623769	1.205910
N	-2.904206	-3.777514	-1.560555
N	-3.585984	2.898365	-1.159113
N	3.791292	-3.012454	0.802543
C	4.394737	3.349597	1.608689
C	-4.236876	-3.423462	-2.039096
C	1.983210	3.379697	2.078367
C	-1.825149	-3.772914	-2.435679
C	0.683262	3.380564	1.679773

C	-0.546470	-3.692914	-2.021259
C	0.326155	3.606980	0.314383
C	-0.187151	-3.541111	-0.610376
C	1.422586	3.921734	-0.547593
C	-1.359232	-3.531112	0.264815
C	2.705041	3.903829	-0.095644
C	-2.614929	-3.623916	-0.209828
C	-1.004625	3.459296	-0.163798
C	1.098289	-3.397863	-0.155957
C	-2.084565	3.059822	0.682080
C	2.277293	-3.382770	-1.023590
C	-3.313681	2.779911	0.172889
C	3.519737	-3.173162	-0.547571
C	-2.600056	3.332983	-1.995914
C	2.707498	-3.023606	1.668962
C	-1.352454	3.627112	-1.540371
C	1.442314	-3.222348	1.254794
C	-4.848958	2.365949	-1.699611
C	5.053705	-2.429860	1.241973
C	-4.783091	0.861785	-1.839452
C	4.992158	-0.919938	1.354845
C	-5.250719	0.038537	-0.816383
C	5.171383	-0.110322	0.229994
C	-5.104089	-1.342990	-0.900358
C	5.018385	1.268349	0.318233
C	-4.489333	-1.929173	-2.006614
C	4.683870	1.867395	1.535494
C	-4.045778	-1.103603	-3.043224
C	4.534230	1.064806	2.664905
C	-4.190664	0.276553	-2.961272
C	4.687671	-0.315831	2.573913
H	4.529567	3.732096	2.622886

H	-4.340926	-3.813399	-3.055654
H	5.046698	3.922575	0.946066
H	-4.971025	-3.941919	-1.413703
H	2.270190	3.170580	3.101275
H	-2.083326	-3.874371	-3.484811
H	-0.060862	3.160457	2.434024
H	0.222198	-3.741563	-2.784177
H	1.273451	4.149307	-1.594439
H	-1.245084	-3.441590	1.338525
H	3.547678	4.106667	-0.744743
H	-3.477885	-3.606734	0.447665
H	-1.950632	2.918629	1.746435
H	2.178039	-3.538099	-2.091794
H	-4.128866	2.436100	0.796842
H	4.388141	-3.152176	-1.197974
H	-2.868867	3.428548	-3.040442
H	2.951290	-2.883012	2.716667
H	-0.630373	3.965082	-2.271466
H	0.673828	-3.241764	2.018186
H	-5.021982	2.851800	-2.661957
H	5.312705	-2.869319	2.210764
H	-5.652914	2.666395	-1.023704
H	5.825362	-2.732864	0.528688
H	-5.732087	0.474255	0.055322
H	5.424417	-0.562770	-0.725310
H	-5.473621	-1.969999	-0.092976
H	5.155122	1.881106	-0.569203
H	-3.578757	-1.543339	-3.920639
H	4.295393	1.515686	3.624580
H	-3.837071	0.902705	-3.776524
H	4.567738	-0.926352	3.465252
C	-0.561724	-0.186363	1.667449

C	-0.888487	0.454898	4.138052
C	-2.122538	0.324502	3.647088
H	-0.655450	0.826199	5.128527
H	-3.029326	0.574573	4.184168
C	-0.077974	-0.037049	0.420441
C	1.386372	0.675940	-1.585370
C	0.154290	0.560103	-2.083955
H	2.250695	1.015349	-2.142723
H	-0.124911	0.790128	-3.104672
S	-2.285428	-0.386735	2.040320
S	0.448706	-0.095134	3.125658
S	-1.102702	-0.045530	-1.019843
S	1.639512	0.210001	0.085929

TTF-CBPQT^0

Atom	X	Y	Z
N	3.018773	3.771022	1.238623
N	-2.885515	-3.746088	-1.650787
N	-3.577147	3.107514	-1.381299
N	3.734108	-3.053606	0.936297
C	4.354659	3.378531	1.673355
C	-4.193582	-3.333883	-2.150297
C	1.930532	3.498758	2.056397
C	-1.778154	-3.662201	-2.486614
C	0.663412	3.475491	1.604058
C	-0.515526	-3.603292	-2.025773
C	0.331401	3.677388	0.194029
C	-0.201371	-3.570732	-0.596700
C	1.509983	3.985796	-0.618065
C	-1.399887	-3.667768	0.238653
C	2.755394	3.991172	-0.105536
C	-2.639674	-3.720653	-0.282961

C	-0.937488	3.554066	-0.309433
C	1.069472	-3.442673	-0.100125
C	-2.099647	3.208218	0.508426
C	2.261563	-3.324897	-0.940743
C	-3.313957	2.979866	-0.025197
C	3.488148	-3.117277	-0.427481
C	-2.514619	3.463880	-2.198290
C	2.638947	-3.178453	1.779029
C	-1.278409	3.711009	-1.724217
C	1.388377	-3.389911	1.327031
C	-4.768952	2.481980	-1.942120
C	4.958512	-2.429787	1.424603
C	-4.670533	0.971504	-2.028648
C	4.869217	-0.918820	1.503939
C	-5.148951	0.170221	-0.991729
C	5.099803	-0.129078	0.373764
C	-5.012951	-1.214584	-1.041261
C	4.957785	1.253020	0.432541
C	-4.397053	-1.831509	-2.129801
C	4.581939	1.880882	1.622648
C	-3.927887	-1.029920	-3.174438
C	4.365656	1.093548	2.753405
C	-4.062580	0.353499	-3.124110
C	4.507784	-0.290533	2.694680
H	4.499218	3.745723	2.694880
H	-4.296480	-3.720357	-3.169134
H	5.075807	3.898697	1.035821
H	-4.956535	-3.825291	-1.538012
H	2.169333	3.329252	3.101174
H	-2.001676	-3.679430	-3.548459
H	-0.114469	3.283316	2.333375
H	0.276644	-3.578984	-2.764861

H	1.408649	4.211171	-1.673784
H	-1.318330	-3.687537	1.319765
H	3.627483	4.204317	-0.715436
H	-3.524595	-3.772286	0.343287
H	-2.008092	3.108225	1.583249
H	2.183694	-3.395058	-2.019251
H	-4.166653	2.704920	0.586985
H	4.365866	-3.018558	-1.057940
H	-2.756965	3.563945	-3.251295
H	2.864096	-3.117032	2.838842
H	-0.528916	4.024621	-2.442081
H	0.609036	-3.503683	2.072258
H	-4.932304	2.913332	-2.934251
H	5.177736	-2.847908	2.412144
H	-5.624560	2.765399	-1.319691
H	5.771655	-2.729634	0.756203
H	-5.639871	0.629756	-0.137171
H	5.398070	-0.599170	-0.560265
H	-5.396852	-1.820661	-0.224152
H	5.143284	1.850055	-0.457088
H	-3.459182	-1.491923	-4.039836
H	4.089691	1.564402	3.693889
H	-3.695537	0.959636	-3.948895
H	4.338374	-0.884599	3.589517
C	-0.186285	0.161305	0.541223
C	1.405499	-0.147270	-1.471392
C	0.179291	-0.247490	-1.985465
H	2.325765	-0.290685	-2.023168
H	-0.044240	-0.480966	-3.018740
C	-0.705361	0.157529	1.783869
C	-0.916567	-0.691637	4.208806
C	-2.143894	-0.796330	3.697406

H	-0.619764	-1.065752	5.181082
H	-2.982183	-1.265857	4.197559
S	-1.177875	0.036322	-0.913323
S	1.547765	0.254384	0.227702
S	-2.437688	-0.040549	2.128691
S	0.284695	0.189783	3.260420

$BIPY^{\bullet+}$-$CBPQT^{4+}$

Atom	X	Y	Z
N	2.897239	3.590011	1.525496
N	-2.752635	-3.550323	-1.466365
N	-3.558960	2.939392	-1.057359
N	3.728734	-2.900675	1.064591
C	4.289400	3.355910	2.001320
C	-4.145395	-3.321539	-1.942932
C	1.872181	3.262589	2.334665
C	-1.728074	-3.258808	-2.289898
C	0.577294	3.266503	1.868994
C	-0.431241	-3.260214	-1.829969
C	0.315352	3.605545	0.537442
C	-0.164673	-3.559661	-0.489561
C	1.393843	4.011393	-0.255574
C	-1.244630	-3.926382	0.321327
C	2.671781	3.988120	0.259230
C	-2.524468	-3.906985	-0.188183
C	-1.049403	3.465402	-0.023577
C	1.205796	-3.423296	0.058403
C	-2.180115	3.449167	0.799740
C	2.329283	-3.416007	-0.775406
C	-3.418436	3.178766	0.260221
C	3.574005	-3.147280	-0.249964
C	-2.499433	3.014071	-1.885264

C	2.677442	-2.968912	1.903304
C	-1.240406	3.278314	-1.396441
C	1.412801	-3.231707	1.428673
C	-4.855935	2.436712	-1.590741
C	5.030496	-2.394526	1.582794
C	-4.781397	0.931760	-1.710358
C	4.946687	-0.891227	1.716431
C	-4.896388	0.136343	-0.566688
C	5.013242	-0.087021	0.574559
C	-4.728367	-1.238964	-0.650354
C	4.836186	1.285643	0.673932
C	-4.442481	-1.840593	-1.879091
C	4.589259	1.876497	1.916732
C	-4.364110	-1.052183	-3.025582
C	4.555759	1.079608	3.059018
C	-4.532700	0.328676	-2.941634
C	4.734114	-0.299329	2.959273
H	4.356938	3.733644	3.021904
H	-4.214662	-3.713841	-2.957894
H	4.951238	3.950250	1.371258
H	-4.806985	-3.906167	-1.303569
H	2.121712	2.968773	3.345947
H	-1.978512	-2.998284	-3.309945
H	-0.201744	2.955176	2.552164
H	0.345959	-2.979530	-2.528030
H	1.268131	4.346831	-1.276517
H	-1.119182	-4.229733	1.352231
H	3.535488	4.270929	-0.327700
H	-3.388158	-4.161869	0.411354
H	-2.124860	3.642371	1.862831
H	2.264744	-3.613793	-1.836930
H	-4.314150	3.134901	0.865171

H	4.463596	-3.110281	-0.864282
H	-2.684430	2.829590	-2.935330
H	2.873145	-2.776739	2.950115
H	-0.420414	3.289757	-2.102110
H	0.601374	-3.236871	2.144188
H	-5.026790	2.920101	-2.552844
H	5.218805	-2.884855	2.538147
H	-5.635853	2.759168	-0.900936
H	5.802197	-2.705352	0.878426
H	-5.115491	0.587019	0.396817
H	5.202530	-0.529609	-0.399059
H	-4.819848	-1.839987	0.249618
H	4.889986	1.893927	-0.224307
H	-4.169690	-1.508084	-3.991582
H	4.388002	1.526678	4.033991
H	-4.466915	0.929379	-3.843841
H	4.702103	-0.907500	3.858282
N	-0.639411	-0.320901	3.415758
C	0.636874	-0.124134	2.984339
H	1.398304	-0.077131	3.753324
C	0.934000	0.004400	1.659851
H	1.976353	0.152121	1.403730
C	-0.085458	-0.059777	0.662731
C	-1.405291	-0.263994	1.163133
H	-2.260649	-0.331376	0.503599
C	-1.645123	-0.385977	2.500320
H	-2.641433	-0.540329	2.893840
C	-0.914124	-0.411374	4.852713
H	-0.184576	-1.075558	5.317113
H	-1.912960	-0.819161	5.000102
N	0.748803	0.325734	-3.485228
C	-0.527561	0.134464	-3.053103

H	-1.289314	0.089190	-3.821810
C	-0.824863	0.009618	-1.728173
H	-1.868139	-0.132001	-1.472384
C	0.195570	0.070647	-0.731716
C	1.515549	0.271127	-1.232737
H	2.370752	0.340206	-0.573147
C	1.755047	0.389598	-2.570387
H	2.751443	0.540699	-2.964916
C	1.024640	0.410444	-4.922399
H	0.288762	1.064008	-5.391690
H	2.019248	0.828134	-5.070774
H	-0.851941	0.581028	5.303831
H	0.973675	-0.585216	-5.367688

$BIPY^{\bullet+}$-$CBPQT^{\bullet 3+}$

Atom	X	Y	Z
N	3.594884	3.648042	-1.639885
N	-2.291150	-3.417643	-4.111940
N	-3.179794	2.928690	-3.507130
N	4.289665	-2.730490	-1.795831
C	5.011432	3.472659	-1.274521
C	-3.706957	-3.262682	-4.550926
C	2.626453	3.513122	-0.687680
C	-1.297203	-3.149423	-4.982020
C	1.307671	3.434648	-1.013304
C	0.009971	-3.075101	-4.559657
C	0.876162	3.472030	-2.375492
C	0.323570	-3.285409	-3.210472
C	1.921643	3.646754	-3.332126
C	-0.726911	-3.611672	-2.344417
C	3.226821	3.716758	-2.951272
C	-2.019014	-3.661821	-2.816193

C	-0.490337	3.327731	-2.751119
C	1.709980	-3.142126	-2.715179
C	-1.532423	3.111072	-1.799013
C	2.804406	-3.172720	-3.587416
C	-2.819737	2.906412	-2.191606
C	4.076693	-2.960026	-3.105212
C	-2.222538	3.169681	-4.450130
C	3.262727	-2.739096	-0.923228
C	-0.920311	3.372196	-4.113605
C	1.972488	-2.943026	-1.353786
C	-4.510456	2.450551	-3.919774
C	5.635939	-2.292639	-1.327409
C	-4.463958	0.953129	-4.128930
C	5.647842	-0.784043	-1.268597
C	-4.765711	0.078152	-3.084443
C	5.901355	-0.039415	-2.422400
C	-4.575205	-1.291994	-3.236260
C	5.750477	1.342577	-2.409954
C	-4.078897	-1.803399	-4.436273
C	5.344354	1.997752	-1.245514
C	-3.826053	-0.934432	-5.498733
C	5.147576	1.257269	-0.079945
C	-4.018559	0.433076	-5.345561
C	5.297751	-0.125269	-0.090376
H	5.167027	3.941388	-0.300893
H	-3.772606	-3.631273	-5.574796
H	5.615556	4.009622	-2.007634
H	-4.318407	-3.903119	-3.914973
H	2.968773	3.472391	0.338621
H	-1.580928	-2.973764	-6.011158
H	0.604773	3.340651	-0.196774
H	0.759767	-2.819870	-5.296159

H	1.718230	3.713601	-4.392531
H	-0.567894	-3.834558	-1.298017
H	4.029776	3.827437	-3.669029
H	-2.859290	-3.886687	-2.173470
H	-1.335034	3.080066	-0.735730
H	2.696281	-3.365012	-4.646235
H	-3.614087	2.714617	-1.481348
H	4.944723	-2.956764	-3.750665
H	-2.559837	3.192899	-5.478505
H	3.499628	-2.553586	0.116011
H	-0.228593	3.565346	-4.922217
H	1.186973	-2.906354	-0.611397
H	-4.785720	2.976301	-4.836023
H	5.807491	-2.751432	-0.353351
H	-5.224582	2.730274	-3.143561
H	6.368431	-2.689598	-2.030115
H	-5.144885	0.463958	-2.142364
H	6.209694	-0.536214	-3.338107
H	-4.808822	-1.958094	-2.410258
H	5.943491	1.909804	-3.316293
H	-3.472268	-1.319930	-6.450620
H	4.866513	1.755629	0.843094
H	-3.813316	1.095661	-6.181282
H	5.133360	-0.686168	0.825125
N	-0.973136	-0.281762	0.367349
C	0.362538	-0.028773	0.273343
H	0.902293	0.027716	1.210255
C	0.976644	0.142098	-0.931234
H	2.039009	0.347932	-0.915366
C	0.240018	0.074034	-2.152878
C	-1.157177	-0.161169	-2.000280
H	-1.825847	-0.205737	-2.850593

C	-1.717823	-0.334874	-0.769412
H	-2.776947	-0.517884	-0.640872
C	-1.597904	-0.422904	1.684569
H	-0.965073	-1.044310	2.318979
H	-2.568504	-0.903418	1.569071
N	2.074712	0.507779	-5.958026
C	0.722161	0.389649	-5.853517
H	0.168839	0.414930	-6.783742
C	0.105231	0.252522	-4.645845
H	-0.975264	0.186021	-4.652337
C	0.855662	0.230421	-3.431435
C	2.264178	0.375259	-3.591302
H	2.936641	0.399069	-2.743337
C	2.829204	0.497469	-4.826081
H	3.898052	0.600988	-4.961817
C	2.704831	0.595009	-7.277436
H	2.080701	1.202048	-7.933558
H	3.680591	1.068785	-7.176205
H	-1.728187	0.560642	2.141407
H	2.823857	-0.404671	-7.701225

$BIPY^{\bullet+}$-$CBPQT^{2(\bullet+)}$(T)

Atom	X	Y	Z
N	1.985538	-4.325995	-0.375799
N	2.705668	2.026047	-3.386783
C	1.608620	-5.506878	0.420190
H	2.500233	-5.853966	0.946010
H	1.297764	-6.291541	-0.271747
C	1.287438	-4.012097	-1.505169
H	0.605125	-4.769604	-1.870085
C	1.440976	-2.813696	-2.131203
H	0.856181	-2.650089	-3.026614

C	2.324073	-1.814596	-1.618813
C	3.046900	-2.195564	-0.445404
H	3.759338	-1.531413	0.024465
C	2.858242	-3.409779	0.137224
H	3.390394	-3.706640	1.031930
C	2.468675	-0.535352	-2.224269
C	1.707384	-0.135904	-3.366762
H	0.998955	-0.802565	-3.839188
C	1.836358	1.107576	-3.901778
H	1.258669	1.426637	-4.759943
C	3.464882	1.682285	-2.306583
H	4.137171	2.444953	-1.933735
C	3.378298	0.450986	-1.733834
H	4.022369	0.259386	-0.885931
C	2.620739	3.433789	-3.812130
H	3.598907	3.891964	-3.656261
H	2.409367	3.447383	-4.883175
C	1.540743	4.131677	-3.017395
C	0.203590	4.014133	-3.402738
H	-0.053879	3.526483	-4.338742
C	-0.808285	4.509520	-2.590569
H	-1.843149	4.401532	-2.902706
C	-0.501806	5.131394	-1.378280
C	0.835591	5.297470	-1.019263
H	1.091402	5.806900	-0.094052
C	1.850082	4.800966	-1.833532
H	2.887367	4.927736	-1.535389
N	-1.986197	4.325432	0.375305
N	-2.706170	-2.026373	3.386865
C	-1.609506	5.506377	-0.420708
H	-2.501163	5.853245	-0.946596
H	-1.298872	6.291119	0.271241

C	-1.288061	4.011695	1.504697
H	-0.605849	4.769310	1.869572
C	-1.441424	2.813303	2.130792
H	-0.856505	2.649786	3.026137
C	-2.324470	1.814100	1.618519
C	-3.047274	2.194877	0.445033
H	-3.759600	1.530596	-0.024818
C	-2.858703	3.409046	-0.137725
H	-3.390743	3.705723	-1.032561
C	-2.469090	0.534930	2.224131
C	-1.708115	0.135695	3.366913
H	-0.999972	0.802507	3.839554
C	-1.837159	-1.107724	3.902063
H	-1.259807	-1.426578	4.760535
C	-3.465069	-1.682812	2.306378
H	-4.137125	-2.445610	1.933377
C	-3.378475	-0.451555	1.733548
H	-4.022318	-0.260116	0.885433
C	-2.621119	-3.434111	3.812198
H	-3.599275	-3.892354	3.656453
H	-2.409624	-3.447688	4.883220
C	-1.541174	-4.131949	3.017336
C	-0.203862	-4.013181	3.401806
H	0.053858	-3.524584	4.337245
C	0.807846	-4.508611	2.589497
H	1.842814	-4.399695	2.900961
C	0.501083	-5.131714	1.377889
C	-0.836397	-5.299010	1.019793
H	-1.092411	-5.809319	0.095119
C	-1.850758	-4.802454	1.834240
H	-2.888129	-4.930127	1.536787
N	2.397488	1.716029	1.947934

C	1.826231	2.315080	0.866370
H	2.149521	3.326889	0.657278
C	0.902463	1.676830	0.096242
H	0.505850	2.227093	-0.748376
C	0.484316	0.344982	0.391701
C	1.111472	-0.243725	1.533833
H	0.883518	-1.252566	1.851960
C	2.029257	0.443254	2.268846
H	2.508662	0.013170	3.139004
C	3.326545	2.466162	2.792567
H	3.967119	3.084801	2.163195
H	3.947542	1.768078	3.353172
N	-2.396604	-1.717203	-1.946688
C	-1.825227	-2.316158	-0.865143
H	-2.148099	-3.328081	-0.655998
C	-0.901646	-1.677726	-0.094957
H	-0.504908	-2.227987	0.749603
C	-0.483717	-0.345813	-0.390367
C	-1.111019	0.242828	-1.532456
H	-0.883270	1.251735	-1.850513
C	-2.028657	-0.444313	-2.267506
H	-2.508158	-0.014275	-3.137635
C	-3.325454	-2.467519	-2.791379
H	-3.965937	-3.086293	-2.162045
H	-3.946563	-1.769562	-3.352019
H	2.770984	3.100842	3.487333
H	-2.769721	-3.102084	-3.486114

BIPY$^{\bullet+}$-CBPQT$^{2(\bullet+)}$(S)

Atom	X	Y	Z
N	3.074116	3.560292	1.093925
N	-3.037941	-3.605318	-1.169430

N	-3.622212	2.864447	-1.053514
N	3.689690	-2.852084	0.840822
C	4.470550	3.365319	1.520698
C	-4.435015	-3.380097	-1.576297
C	2.061142	3.478551	2.006047
C	-2.040183	-3.605850	-2.101909
C	0.757152	3.402984	1.625034
C	-0.728225	-3.542231	-1.747507
C	0.387344	3.377959	0.243648
C	-0.333499	-3.448737	-0.376958
C	1.478774	3.491572	-0.671234
C	-1.410203	-3.493944	0.562332
C	2.765022	3.568501	-0.235205
C	-2.704798	-3.558961	0.153268
C	-0.959835	3.233785	-0.190018
C	1.020759	-3.286844	0.026298
C	-2.060961	3.168441	0.717328
C	2.108838	-3.232143	-0.898951
C	-3.333734	2.975524	0.274916
C	3.384967	-3.008581	-0.480238
C	-2.603353	2.947795	-1.958950
C	2.684075	-2.922208	1.761479
C	-1.313353	3.132598	-1.572367
C	1.391858	-3.133211	1.397972
C	-4.952508	2.406838	-1.490667
C	5.029748	-2.406577	1.263429
C	-4.970101	0.896894	-1.564785
C	5.054367	-0.900061	1.389286
C	-5.397773	0.134010	-0.478436
C	5.454434	-0.103550	0.316840
C	-5.272815	-1.252991	-0.499134
C	5.325046	1.281158	0.381406

C	-4.718042	-1.895234	-1.605858
C	4.790459	1.888081	1.517651
C	-4.331839	-1.134325	-2.711096
C	4.445637	1.094867	2.613820
C	-4.458235	0.248432	-2.690977
C	4.580728	-0.286386	2.551200
H	4.580606	3.803499	2.514422
H	-4.574729	-3.839117	-2.556912
H	5.113028	3.920117	0.834417
H	-5.081122	-3.899235	-0.865930
H	2.356107	3.479506	3.047826
H	-2.354562	-3.657732	-3.136697
H	0.017756	3.348156	2.412493
H	-0.000907	-3.548326	-2.548392
H	1.323307	3.512166	-1.741389
H	-1.236857	-3.458939	1.629240
H	3.601077	3.636373	-0.919878
H	-3.530141	-3.565569	0.854166
H	-1.921135	3.257476	1.786361
H	1.955291	-3.350656	-1.963299
H	-4.173710	2.902050	0.954101
H	4.214270	-2.941404	-1.173168
H	-2.882168	2.858923	-3.001092
H	2.974345	-2.801105	2.796946
H	-0.569738	3.187258	-2.355700
H	0.660255	-3.169391	2.193548
H	-5.157729	2.861097	-2.462125
H	5.255431	-2.893286	2.214596
H	-5.688305	2.785711	-0.778943
H	5.748963	-2.762813	0.524068
H	-5.826594	0.620526	0.393449
H	5.858709	-0.563162	-0.581037

H	-5.603084	-1.833974	0.357739
H	5.629585	1.888189	-0.466974
H	-3.926028	-1.620221	-3.593765
H	4.061200	1.553537	3.520406
H	-4.152899	0.823199	-3.560623
H	4.308038	-0.886124	3.414823
N	-2.018647	0.010245	3.135615
C	-2.609278	-0.129658	1.914505
H	-3.685087	-0.253029	1.917684
C	-1.885883	-0.116376	0.762231
H	-2.436895	-0.230018	-0.163124
C	-0.466404	0.041432	0.778182
C	0.109624	0.169880	2.079575
H	1.176732	0.281990	2.219947
C	-0.663925	0.153609	3.200366
H	-0.241626	0.251919	4.192260
C	-2.824789	-0.073266	4.353218
H	-3.777726	0.431163	4.189930
H	-2.297642	0.421119	5.168879
N	1.855254	0.049451	-2.773998
C	2.448649	0.218382	-1.559646
H	3.523416	0.343447	-1.570942
C	1.732531	0.222045	-0.402279
H	2.288209	0.353781	0.518607
C	0.313721	0.061356	-0.411163
C	-0.266429	-0.094266	-1.707678
H	-1.331467	-0.230247	-1.842763
C	0.501343	-0.095358	-2.831997
H	0.075351	-0.218416	-3.819519
C	2.660331	0.104179	-3.993888
H	3.608122	-0.407630	-3.823426
H	2.126466	-0.397611	-4.800538

H	-3.003405	-1.119359	4.613900
H	2.849201	1.144166	-4.271335

BIPY$^{\bullet+}$-CBPQT$^{\bullet+}$

Atom	X	Y	Z
N	2.983155	3.538630	1.085699
N	-3.016037	-3.687335	-1.189045
N	-3.748295	2.845262	-0.943048
N	3.758597	-2.993242	0.919914
C	4.383835	3.330082	1.488230
C	-4.395862	-3.423953	-1.588959
C	1.986404	3.425986	2.013100
C	-1.995377	-3.455494	-2.099759
C	0.678120	3.347300	1.654477
C	-0.712464	-3.277549	-1.731027
C	0.282525	3.360520	0.278898
C	-0.295797	-3.287322	-0.331885
C	1.356423	3.511220	-0.651922
C	-1.415626	-3.466819	0.586833
C	2.650092	3.578268	-0.236978
C	-2.680271	-3.635373	0.156189
C	-1.070456	3.220926	-0.128814
C	1.010238	-3.154048	0.073360
C	-2.141458	3.047667	0.801395
C	2.133857	-3.012870	-0.847448
C	-3.420164	2.852234	0.381439
C	3.407779	-2.923712	-0.420881
C	-2.761151	3.036549	-1.867690
C	2.719759	-3.061505	1.837282
C	-1.465668	3.227999	-1.504596
C	1.426342	-3.151044	1.472726
C	-5.068086	2.352124	-1.370480

C	5.045767	-2.449797	1.346095
C	-5.033905	0.845187	-1.474651
C	5.008707	-0.938880	1.428771
C	-5.426751	0.046826	-0.400821
C	5.403105	-0.152409	0.346020
C	-5.262832	-1.335124	-0.453697
C	5.248135	1.231057	0.380490
C	-4.703641	-1.942044	-1.578372
C	4.693582	1.851337	1.499521
C	-4.343535	-1.142231	-2.666274
C	4.331104	1.069261	2.598622
C	-4.506468	0.236181	-2.615609
C	4.487295	-0.310410	2.562527
H	4.516184	3.779176	2.474898
H	-4.542819	-3.844202	-2.588218
H	5.019648	3.870717	0.784498
H	-5.056609	-3.963189	-0.904454
H	2.300164	3.406786	3.049136
H	-2.296859	-3.454902	-3.141563
H	-0.046863	3.269950	2.453226
H	0.013092	-3.134057	-2.522554
H	1.181587	3.553404	-1.718722
H	-1.256684	-3.467645	1.658675
H	3.474004	3.662155	-0.934583
H	-3.508972	-3.759654	0.844907
H	-1.970039	3.039068	1.869523
H	1.969877	-2.972597	-1.917678
H	-4.235072	2.687882	1.075393
H	4.237421	-2.810416	-1.110401
H	-3.072209	3.029632	-2.904818
H	3.022729	-3.066407	2.878720
H	-0.749200	3.381024	-2.300220

H	0.694236	-3.231789	2.267060
H	-5.301971	2.819172	-2.329322
H	5.289654	-2.886658	2.318758
H	-5.806063	2.691530	-0.640913
H	5.806868	-2.781839	0.634382
H	-5.859253	0.503500	0.485720
H	5.831461	-0.621964	-0.535699
H	-5.569362	-1.944603	0.392646
H	5.557641	1.828508	-0.473396
H	-3.932846	-1.598585	-3.562523
H	3.924644	1.537354	3.490982
H	-4.220419	0.839865	-3.472837
H	4.200878	-0.902685	3.427232
N	-1.896934	-0.236841	2.949874
C	-2.508409	-0.311764	1.733781
H	-3.579224	-0.470379	1.748433
C	-1.811169	-0.203221	0.570735
H	-2.377403	-0.287932	-0.348652
C	-0.396743	-0.011963	0.569235
C	0.204721	0.041832	1.866025
H	1.270956	0.176605	1.991815
C	-0.542977	-0.067657	2.997482
H	-0.103063	-0.024143	3.985830
C	-2.673976	-0.427304	4.172433
H	-3.644728	0.057804	4.062306
H	-2.145686	0.026748	5.010674
N	1.848987	0.423980	-3.004674
C	2.471268	0.400129	-1.791823
H	3.549547	0.495720	-1.807966
C	1.777952	0.251425	-0.630853
H	2.357880	0.215373	0.283411
C	0.356441	0.120962	-0.627266

C	-0.252272	0.135656	-1.921983
H	-1.321174	0.023496	-2.047715
C	0.490442	0.293011	-3.050901
H	0.043515	0.317865	-4.036774
C	2.618432	0.674546	-4.220884
H	3.596268	0.199655	-4.132756
H	2.094181	0.245919	-5.075055
H	-2.818481	-1.493217	4.367190
H	2.747415	1.749338	-4.373994

$BIPY^{\bullet+}$-$CBPQT^0$

Atom	X	Y	Z
N	3.207379	3.952582	1.474633
N	-3.154313	-4.058597	-1.546723
N	-3.277405	2.770041	-1.262857
N	3.327492	-2.876131	1.197579
C	4.496134	3.476888	1.975796
C	-4.443220	-3.582575	-2.047117
C	2.088244	3.767716	2.285976
C	-2.034950	-3.870782	-2.357079
C	0.850338	3.580252	1.797021
C	-0.797366	-3.683907	-1.867072
C	0.579439	3.493189	0.362404
C	-0.527258	-3.600228	-0.432116
C	1.773978	3.725364	-0.448544
C	-1.721855	-3.836285	0.377657
C	2.988163	3.911983	0.099106
C	-2.935669	-4.022344	-0.171002
C	-0.657018	3.220170	-0.164738
C	0.708439	-3.326582	0.096407
C	-1.843593	2.968034	0.648747
C	1.895491	-3.072132	-0.715649

C	-3.056643	2.755202	0.103873
C	3.107932	-2.859487	-0.169373
C	-2.169439	2.911831	-2.080690
C	2.218731	-3.019024	2.014205
C	-0.933545	3.143286	-1.597728
C	0.983466	-3.250761	1.529766
C	-4.529218	2.253587	-1.803357
C	4.578606	-2.359624	1.739675
C	-4.560121	0.741985	-1.889961
C	4.610252	-0.847933	1.824572
C	-4.838219	-0.023927	-0.754596
C	4.892612	-0.083645	0.689178
C	-4.827021	-1.411713	-0.815391
C	4.882231	1.304231	0.748183
C	-4.539212	-2.071682	-2.013748
C	4.591277	1.965904	1.944810
C	-4.268240	-1.307082	-3.148600
C	4.316126	1.202933	3.079772
C	-4.279539	0.085021	-3.087014
C	4.326333	-0.189238	3.019914
H	4.623194	3.851186	2.996153
H	-4.570131	-3.955143	-3.068132
H	5.281746	3.925910	1.360000
H	-5.228626	-4.033040	-1.432112
H	2.284970	3.815019	3.352377
H	-2.231250	-3.915127	-3.423691
H	0.044134	3.496456	2.516520
H	0.009095	-3.597546	-2.585988
H	1.706317	3.763602	-1.529619
H	-1.654374	-3.878631	1.458593
H	3.872319	4.077705	-0.508412
H	-3.819860	-4.191266	0.435602

H	-1.776714	2.941583	1.729714
H	1.829521	-3.044022	-1.796634
H	-3.935525	2.578867	0.714063
H	3.987262	-2.681906	-0.778544
H	-2.366278	2.837232	-3.144720
H	2.414432	-2.945017	3.078499
H	-0.135322	3.254412	-2.321708
H	0.184449	-3.362314	2.252771
H	-4.676180	2.695787	-2.792907
H	4.723546	-2.800809	2.729966
H	-5.341334	2.613987	-1.164369
H	5.391747	-2.721164	1.102622
H	-5.079723	0.466757	0.184523
H	5.137021	-0.575638	-0.248504
H	-5.056247	-1.987553	0.077293
H	5.114748	1.878801	-0.144475
H	-4.052670	-1.800911	-4.093062
H	4.098161	1.698111	4.022984
H	-4.071426	0.663068	-3.984191
H	4.114857	-0.766019	3.917118
N	-0.374876	-0.316263	3.464983
C	-1.375474	-0.705836	2.619685
H	-2.267314	-1.101318	3.089927
C	-1.255123	-0.596146	1.270058
H	-2.094884	-0.926177	0.672072
C	-0.058030	-0.097477	0.667983
C	0.956532	0.302562	1.589455
H	1.894720	0.729019	1.257585
C	0.777088	0.186866	2.931388
H	1.536262	0.491916	3.640407
C	-0.484379	-0.523556	4.905258
H	-1.537226	-0.567777	5.184657

H	-0.014602	0.310790	5.428369
N	0.422603	0.212174	-3.533630
C	1.426276	0.595425	-2.687600
H	2.319020	0.987637	-3.159239
C	1.306403	0.484264	-1.338560
H	2.148139	0.808946	-0.740388
C	0.107512	-0.011081	-0.736655
C	-0.908537	-0.406067	-1.658223
H	-1.848046	-0.829744	-1.326662
C	-0.730423	-0.286780	-3.000520
H	-1.492663	-0.586773	-3.708124
C	0.546256	0.435189	-4.970349
H	1.575874	0.249868	-5.279933
H	-0.110966	-0.254367	-5.500710
H	0.007064	-1.457107	5.193666
H	0.270774	1.463781	-5.220414

NDI$^{\bullet-}$-CBPQT^{4+}

Atom	X	Y	Z
N	2.697542	3.208650	1.461452
N	-2.772337	-3.264440	-1.408393
N	-3.797786	2.816795	-1.357350
N	3.728782	-2.868753	1.397621
C	4.085217	3.182945	2.049513
C	-4.159815	-3.237954	-1.996298
C	1.613420	3.122958	2.258132
C	-1.689298	-3.173908	-2.205853
C	0.333054	3.148517	1.737494
C	-0.408505	-3.198786	-1.686707
C	0.119430	3.259176	0.356309
C	-0.192679	-3.313229	-0.306218
C	1.270389	3.355082	-0.439403

C	-1.342858	-3.415153	0.490553
C	2.528387	3.324241	0.129437
C	-2.601412	-3.385787	-0.076787
C	-1.253550	3.204031	-0.243641
C	1.181138	-3.256594	0.290534
C	-2.413928	3.210141	0.541583
C	2.338853	-3.255139	-0.498448
C	-3.656372	3.009604	-0.031383
C	3.582773	-3.054111	0.071318
C	-2.709095	2.867152	-2.151109
C	2.642441	-2.926578	2.195126
C	-1.447924	3.068704	-1.624967
C	1.380303	-3.128500	1.672459
C	-5.134351	2.431686	-1.933811
C	5.067097	-2.484579	1.970608
C	-5.233174	0.924250	-2.070948
C	5.165269	-0.977606	2.113473
C	-5.567719	0.135789	-0.963492
C	5.510500	-0.183534	1.012938
C	-5.315838	-1.231552	-0.973098
C	5.256324	1.183541	1.026279
C	-4.723962	-1.834582	-2.088993
C	4.651503	1.780312	2.138972
C	-4.580179	-1.083336	-3.256104
C	4.496404	1.023348	3.300665
C	-4.835210	0.288089	-3.248013
C	4.753922	-0.347379	3.288931
H	4.006190	3.651003	3.030939
H	-4.081760	-3.708463	-2.976627
H	4.692315	3.829170	1.414976
H	-4.768126	-3.881432	-1.360116
H	1.793511	3.023645	3.320473

H	-1.870979	-3.070761	-3.267562
H	-0.479963	3.051362	2.444996
H	0.403403	-3.097949	-2.394830
H	1.226155	3.458869	-1.515025
H	-1.296986	-3.522580	1.565769
H	3.424995	3.393152	-0.472412
H	-3.497230	-3.459830	0.525572
H	-2.390011	3.350317	1.614144
H	2.311897	-3.389653	-1.571640
H	-4.558982	2.983412	0.564573
H	4.482879	-3.021617	-0.528119
H	-2.862416	2.695320	-3.208146
H	2.799147	-2.762275	3.252723
H	-0.625172	3.062088	-2.327150
H	0.559798	-3.128242	2.377399
H	-5.219932	2.939841	-2.894700
H	5.156530	-2.996384	2.929216
H	-5.889435	2.836314	-1.259555
H	5.820124	-2.886072	1.292137
H	-5.959497	0.596765	-0.061230
H	5.911718	-0.640059	0.112636
H	-5.518206	-1.812017	-0.077595
H	5.467123	1.768787	0.135850
H	-4.178914	-1.537892	-4.155617
H	4.084992	1.472941	4.198040
H	-4.622337	0.867741	-4.140094
H	4.533440	-0.931081	4.176512
C	0.971502	-0.045333	1.556830
C	-0.135148	-0.033333	0.699658
C	2.260062	-0.038858	1.017825
C	0.777923	-0.122299	2.999475
C	0.087531	-0.017675	-0.709107

C	-1.442885	-0.032600	1.216034
C	1.396001	-0.016107	-1.223079
C	-1.018772	-0.006834	-1.567826
C	2.480990	-0.022278	-0.342769
C	1.619673	-0.006584	-2.664274
C	-1.675468	-0.036446	2.655805
C	-2.527147	-0.026548	0.333970
C	-0.833689	0.073273	-3.011341
C	-2.307539	-0.012436	-1.025808
C	0.715218	0.049721	-4.916219
C	-0.704156	-0.109469	4.915503
H	-3.529644	-0.033513	0.742280
H	-3.122797	0.009892	-1.719875
H	1.191179	0.991659	-5.199422
H	3.073527	-0.064102	1.713637
H	3.484047	-0.014169	-0.750670
H	-1.767379	-0.038656	5.128802
H	-0.171331	0.726241	5.373159
H	-0.300481	-1.043016	5.313128
H	-0.246768	-0.043473	-5.413347
H	1.372668	-0.774025	-5.197062
N	0.482183	0.016015	-3.475681
N	-0.533431	-0.068831	3.466453
O	-1.772908	0.198000	-3.798957
O	-2.799745	-0.009459	3.151027
O	2.736452	-0.020028	-3.176666
O	1.705308	-0.241661	3.801600

$NDI^{\bullet-}$-$CBPQT^{\bullet 3+}$

Atom	X	Y	Z
N	-4.463161	-2.278343	1.190983
N	4.360235	2.390968	-0.963724

N	2.601842	-3.204891	0.775131
N	-2.622688	3.412165	-0.991712
C	-5.787660	-1.645726	1.048233
C	5.764100	1.844010	-0.862215
C	-3.634416	-1.934171	2.232768
C	3.671845	2.267186	-2.116058
C	-2.312701	-2.248903	2.260802
C	2.332224	2.597005	-2.193741
C	-1.654397	-2.928545	1.169360
C	1.650427	3.058635	-1.061751
C	-2.594266	-3.374590	0.156883
C	2.413104	3.255525	0.094257
C	-3.901910	-3.018158	0.168757
C	3.751889	2.908718	0.122144
C	-0.262905	-3.083279	1.074644
C	0.165134	3.236443	-1.060281
C	0.670997	-2.609484	2.072739
C	-0.639547	2.530844	-1.962923
C	2.016406	-2.676532	1.907848
C	-2.015188	2.628713	-1.904069
C	1.755799	-3.725736	-0.178679
C	-1.876157	4.116415	-0.117168
C	0.403549	-3.679289	-0.067336
C	-0.494905	4.046965	-0.129524
C	4.066160	-3.413063	0.715107
C	-4.127193	3.514959	-0.985495
C	4.850909	-2.173099	0.315367
C	-4.805299	2.263259	-0.471274
C	5.288076	-1.253718	1.270284
C	-5.263020	1.274412	-1.346665
C	5.718119	0.017282	0.888052
C	-5.698958	0.043137	-0.857084

C	5.722727	0.383477	-0.459402
C	-5.680399	-0.223823	0.514455
C	5.491189	-0.607703	-1.421468
C	-5.389776	0.829731	1.388748
C	5.064798	-1.873415	-1.034830
C	-4.953529	2.057244	0.903637
H	-6.267704	-1.654413	2.030601
H	6.224746	1.996854	-1.838841
H	-6.382097	-2.273681	0.380987
H	6.286146	2.462440	-0.131944
H	-4.103114	-1.396956	3.049342
H	4.211315	1.875298	-2.967957
H	-1.758579	-1.925214	3.133662
H	1.842155	2.468503	-3.150086
H	-2.262562	-3.972098	-0.684446
H	1.975180	3.619111	1.015460
H	-4.579772	-3.309822	-0.624045
H	4.342272	2.990600	1.024263
H	0.319473	-2.158723	2.993515
H	-0.218746	1.863937	-2.703920
H	2.703016	-2.306292	2.659972
H	-2.657261	2.077700	-2.579153
H	2.237666	-4.157869	-1.047748
H	-2.408176	4.740637	0.588229
H	-0.164261	-4.103743	-0.886828
H	0.041934	4.648576	0.592879
H	4.244575	-4.221207	0.002515
H	-4.366553	4.382868	-0.371099
H	4.388917	-3.768193	1.698074
H	-4.414695	3.738744	-2.014004
H	5.201206	-1.483269	2.327964
H	-5.224196	1.432606	-2.420563

H	5.942748	0.752225	1.653628
H	-5.961007	-0.741232	-1.559383
H	5.570491	-0.373895	-2.479641
H	-5.440570	0.672708	2.462671
H	4.812751	-2.605006	-1.797447
H	-4.672632	2.836960	1.606072
C	-0.717598	0.694242	1.472495
C	0.237647	0.216875	0.556009
C	-2.062034	0.605813	1.173877
C	-0.291163	1.250474	2.761720
C	-0.180379	-0.366970	-0.663916
C	1.605803	0.285864	0.844438
C	-1.555223	-0.483537	-0.921224
C	0.767912	-0.833668	-1.588674
C	-2.472997	0.018793	-0.022976
C	-2.017702	-1.145879	-2.153284
C	2.065508	0.837460	2.123554
C	2.523411	-0.109694	-0.121126
C	0.340570	-1.520109	-2.814593
C	2.119209	-0.649200	-1.328588
C	-1.519411	-2.371769	-4.182505
C	1.492265	1.801468	4.310500
H	3.563811	0.012686	0.102893
H	2.842461	-0.972191	-2.065349
H	-2.219652	-3.152231	-3.880742
H	-2.784429	0.977590	1.886723
H	-3.514990	-0.100154	-0.249962
H	2.562338	1.644008	4.414970
H	0.952969	1.291522	5.109887
H	1.267391	2.869633	4.358712
H	-0.658640	-2.807306	-4.683013
H	-2.031367	-1.679385	-4.854767

N	-1.041010	-1.665405	-2.997877
N	1.078847	1.257383	3.020094
O	1.131556	-1.952929	-3.642614
O	3.253198	0.966389	2.404788
O	-3.201360	-1.270339	-2.436802
O	-1.074853	1.683014	3.597722

$NDI^{\bullet-}$-$CBPQT^{2(\bullet+)}$(T)

Atom	X	Y	Z
N	4.180485	2.778714	-0.634716
N	-4.181922	-2.802018	-0.513370
N	-2.895303	3.311130	-0.528168
N	2.893588	-3.331659	-0.380961
C	5.574837	2.281390	-0.648218
C	-5.576175	-2.305568	-0.548706
C	3.521587	2.994329	0.543195
C	-3.463085	-2.922820	-1.670638
C	2.179939	3.228534	0.590630
C	-2.124124	-3.160006	-1.669082
C	1.374321	3.218182	-0.591749
C	-1.376152	-3.240683	-0.450768
C	2.122637	3.084493	-1.805276
C	-2.181762	-3.197623	0.730855
C	3.461811	2.848822	-1.796225
C	-3.523293	-2.965265	0.672886
C	-0.050961	3.278681	-0.566597
C	0.049069	-3.301304	-0.422355
C	-0.814037	3.313501	0.644674
C	0.846172	-3.336192	-1.609583
C	-2.176930	3.320992	0.634341
C	2.208018	-3.348001	-1.561192
C	-2.209330	3.263315	-1.707134

C	2.174584	-3.280756	0.780200
C	-0.847496	3.249645	-1.754223
C	0.811717	-3.272611	0.789399
C	-4.377573	3.336284	-0.518175
C	4.375831	-3.354231	-0.367786
C	-4.985132	1.949366	-0.556629
C	4.982345	-1.969804	-0.469586
C	-5.388694	1.307084	0.616272
C	5.044301	-1.319235	-1.702479
C	-5.694486	-0.051840	0.607731
C	5.348152	0.034966	-1.771111
C	-5.600714	-0.789746	-0.574951
C	5.599115	0.765687	-0.608291
C	-5.355051	-0.109938	-1.769205
C	5.696783	0.079733	0.604848
C	-5.051167	1.245985	-1.760065
C	5.389965	-1.277357	0.673024
H	6.086524	2.715396	0.213126
H	-6.049748	-2.731794	-1.435872
H	6.048481	2.668834	-1.553000
H	-6.087830	-2.701266	0.330925
H	4.126980	2.951563	1.440163
H	-4.020566	-2.809482	-2.591228
H	1.742953	3.374509	1.569960
H	-1.642340	-3.228718	-2.635540
H	1.641609	3.113522	-2.774108
H	-1.744875	-3.301663	1.715579
H	4.019660	2.697022	-2.710997
H	-4.128803	-2.882903	1.566868
H	-0.333954	3.302629	1.614539
H	0.395411	-3.351731	-2.593270
H	-2.755193	3.327184	1.549659

H	2.808107	-3.374932	-2.461364
H	-2.809021	3.241519	-2.607704
H	2.752400	-3.238716	1.694876
H	-0.396505	3.207602	-2.736933
H	0.331404	-3.209208	1.757098
H	-4.689337	3.928395	-1.381706
H	4.680006	-3.851231	0.555304
H	-4.682442	3.876633	0.379982
H	4.689834	-3.986065	-1.201771
H	-5.395217	1.849234	1.557975
H	4.782106	-1.847784	-2.613653
H	-5.932917	-0.552352	1.542425
H	5.308998	0.537320	-2.732584
H	-5.320736	-0.652836	-2.708640
H	5.939401	0.619285	1.516476
H	-4.793646	1.735843	-2.693944
H	5.399475	-1.778938	1.636767
C	1.227925	0.028700	2.363178
C	-0.000663	0.038881	1.670551
C	2.434340	0.006174	1.646255
C	1.237597	0.058830	3.816816
C	0.000892	0.003998	0.244056
C	-1.230401	0.082357	2.360980
C	1.224687	-0.030929	-0.445091
C	-1.220718	0.007692	-0.451232
C	2.424920	-0.018938	0.268732
C	1.242218	-0.066825	-1.899595
C	-1.252136	0.122278	3.814595
C	-2.434470	0.072876	1.638478
C	-1.243228	-0.030296	-1.906205
C	-2.422170	0.034775	0.261675
C	0.039252	-0.126850	-3.997680

C	0.025855	0.144996	5.913421
H	-3.369889	0.088947	2.185416
H	-3.333508	0.003144	-0.308335
H	0.527853	0.766343	-4.395440
H	3.368572	0.016966	2.195835
H	3.336933	-0.008822	-0.300959
H	-0.999763	0.211921	6.267418
H	0.600323	1.009758	6.252688
H	0.499536	-0.759081	6.303455
H	-0.985205	-0.182859	-4.356307
H	0.604585	-1.003058	-4.323045
N	0.002914	-0.075397	-2.542875
N	-0.005188	0.107997	4.456401
O	-2.278831	-0.033179	-2.573822
O	-2.293528	0.165106	4.475911
O	2.268666	-0.086352	-2.580873
O	2.266795	0.046190	4.498023

$NDI^{\bullet-}$-$CBPQT^{2(\bullet+)}$(S)

Atom	X	Y	Z
N	2.892563	3.350681	1.515394
N	-3.119190	-3.547557	-1.008711
N	-3.777324	2.630298	-0.805005
N	3.572519	-2.827947	1.251135
C	4.290801	3.256611	1.995082
C	-4.528899	-3.458442	-1.454837
C	1.849954	3.227554	2.389816
C	-2.088626	-3.477391	-1.903936
C	0.554826	3.198248	1.972197
C	-0.784881	-3.446551	-1.507849
C	0.208740	3.260384	0.584588
C	-0.419830	-3.452337	-0.124509

C	1.328945	3.431236	-0.288953
C	-1.529633	-3.566251	0.771601
C	2.607187	3.456452	0.183048
C	-2.814321	-3.594450	0.322413
C	-1.131537	3.110162	0.117079
C	0.925144	-3.302724	0.330417
C	-2.235721	2.886711	0.999235
C	2.034661	-3.119741	-0.553900
C	-3.490960	2.646361	0.530425
C	3.291575	-2.878996	-0.085664
C	-2.759088	2.867366	-1.684993
C	2.547494	-3.026813	2.132573
C	-1.484293	3.113487	-1.269673
C	1.273634	-3.269939	1.718254
C	-5.134407	2.268591	-1.277494
C	4.924311	-2.466817	1.737168
C	-5.301894	0.767451	-1.398219
C	5.063268	-0.971244	1.936937
C	-5.522223	-0.003841	-0.255912
C	5.595535	-0.154691	0.936156
C	-5.376399	-1.385022	-0.298723
C	5.443313	1.228630	0.999646
C	-5.009542	-2.021948	-1.484941
C	4.756125	1.816222	2.064498
C	-4.954409	-1.268870	-2.660327
C	4.372814	1.010946	3.138388
C	-5.099668	0.115820	-2.617255
C	4.525017	-0.368627	3.075133
H	4.326426	3.736282	2.975746
H	-4.587965	-3.920170	-2.442403
H	4.909180	3.844709	1.314240
H	-5.122862	-4.064399	-0.767005

H	2.112081	3.151686	3.437086
H	-2.369279	-3.434849	-2.948837
H	-0.200143	3.094778	2.740611
H	-0.039258	-3.375898	-2.289069
H	1.202707	3.507421	-1.360961
H	-1.389657	-3.601659	1.844032
H	3.458978	3.548666	-0.479011
H	-3.654305	-3.651577	1.002175
H	-2.107440	2.870393	2.073547
H	1.907010	-3.124348	-1.628491
H	-4.320500	2.454130	1.197994
H	4.126184	-2.707207	-0.753352
H	-3.021382	2.836629	-2.734999
H	2.804990	-2.977756	3.182501
H	-0.742171	3.272500	-2.041249
H	0.530432	-3.410695	2.492297
H	-5.286659	2.764776	-2.237824
H	5.084363	-3.009481	2.671522
H	-5.845627	2.693175	-0.565184
H	5.644247	-2.841038	1.006532
H	-5.729497	0.476316	0.695268
H	6.065861	-0.600155	0.063725
H	-5.471355	-1.955088	0.620127
H	5.797037	1.843089	0.176014
H	-4.723306	-1.753671	-3.604958
H	3.881848	1.449857	4.001219
H	-4.980049	0.693451	-3.529802
H	4.149842	-0.979447	3.890311
C	1.057973	0.029532	0.573525
C	0.124819	-0.051038	-0.473915
C	2.411814	0.215883	0.286083
C	0.614537	-0.076713	1.954277

C	0.575484	0.053256	-1.824520
C	-1.244131	-0.232407	-0.212015
C	1.950210	0.238042	-2.082177
C	-0.362091	-0.028518	-2.876205
C	2.857388	0.318386	-1.013500
C	2.419653	0.346307	-3.453079
C	-1.726202	-0.341279	1.156092
C	-2.147771	-0.306129	-1.275486
C	0.078026	0.074964	-4.257885
C	-1.724022	-0.208867	-2.581955
C	1.945268	0.374580	-5.828330
C	-1.181382	-0.363725	3.549816
H	-3.184908	-0.445321	-1.030826
H	-2.431707	-0.268332	-3.400613
H	2.447593	1.334933	-5.964949
H	3.087487	0.276648	1.119902
H	3.908805	0.465358	-1.231130
H	-2.259669	-0.499424	3.558858
H	-0.910470	0.544155	4.094188
H	-0.690158	-1.214093	4.028900
H	1.093043	0.298830	-6.498737
H	2.661652	-0.422809	-6.039205
N	1.453578	0.259025	-4.460859
N	-0.754719	-0.258651	2.161383
O	-0.691870	0.008966	-5.221070
O	-2.911600	-0.498536	1.454009
O	3.603721	0.508194	-3.763873
O	1.362740	-0.017360	2.931541

$NDI^{\bullet-}$-$CBPQT^{\bullet+}$

Atom	X	Y	Z
N	4.283072	2.781458	0.529479

N	-4.245001	-2.689263	-0.569462
N	-2.853806	3.164975	1.065271
N	2.837512	-3.132515	-1.082171
C	5.634636	2.277265	0.270009
C	-5.637528	-2.232313	-0.327544
C	3.630066	2.507741	1.725347
C	-3.598719	-2.364806	-1.730658
C	2.310849	2.718719	1.908279
C	-2.265637	-2.585267	-1.904472
C	1.425404	3.187334	0.835830
C	-1.440901	-3.119165	-0.861481
C	2.195435	3.606885	-0.340289
C	-2.178646	-3.530371	0.294533
C	3.518692	3.375650	-0.466872
C	-3.517555	-3.297468	0.414969
C	0.051408	3.196037	0.911640
C	-0.014447	-3.162097	-0.943251
C	-0.711595	2.751108	2.081678
C	0.711619	-2.708226	-2.092009
C	-2.059762	2.742979	2.121337
C	2.073244	-2.703104	-2.130290
C	-2.187313	3.574665	-0.079026
C	2.189967	-3.565015	0.040525
C	-0.839786	3.609457	-0.177265
C	0.827859	-3.598825	0.129589
C	-4.316816	3.180850	1.169264
C	4.320880	-3.153488	-1.184449
C	-4.990305	1.852583	0.847900
C	4.977158	-1.820657	-0.879173
C	-5.094972	0.850291	1.818630
C	5.052170	-0.821434	-1.854926
C	-5.430731	-0.452600	1.465212

C	5.388687	0.482849	-1.509298
C	-5.674492	-0.783681	0.127747
C	5.657468	0.821067	-0.178846
C	-5.762819	0.253424	-0.802986
C	5.774646	-0.215806	0.749560
C	-5.424608	1.558224	-0.445261
C	5.432332	-1.523286	0.405654
H	6.218480	2.396751	1.188663
H	-6.188460	-2.369576	-1.260830
H	6.084541	2.921251	-0.491906
H	-6.066953	-2.903020	0.419703
H	4.263300	2.117932	2.515500
H	-4.200171	-1.884994	-2.492049
H	1.912225	2.494946	2.891924
H	-1.844057	-2.272383	-2.850990
H	1.703412	4.098830	-1.172662
H	-1.689627	-3.998445	1.139661
H	4.066458	3.659957	-1.359843
H	-4.063835	-3.568431	1.310079
H	-0.198595	2.373435	2.959077
H	0.202158	-2.331644	-2.969386
H	-2.599706	2.390657	2.993233
H	2.614451	-2.351174	-2.999145
H	-2.825888	3.861736	-0.907810
H	2.822457	-3.872522	0.863938
H	-0.428945	3.926424	-1.129730
H	0.411006	-3.942245	1.068086
H	-4.684905	3.957871	0.492970
H	4.670824	-3.924478	-0.495233
H	-4.572122	3.492051	2.187816
H	4.557609	-3.485036	-2.198588
H	-4.834955	1.068369	2.851313

H	4.776204	-1.042452	-2.882937
H	-5.421687	-1.230873	2.224242
H	5.359286	1.260292	-2.268695
H	-5.991230	0.032251	-1.841273
H	6.024769	0.009947	1.782235
H	-5.401362	2.326266	-1.212295
H	5.424837	-2.288433	1.175201
C	-1.558399	0.370805	-0.880773
C	-0.194139	0.259807	-0.586393
C	-2.506338	-0.022075	0.066574
C	-1.986155	0.856105	-2.180397
C	0.198222	-0.282567	0.673805
C	0.779429	0.662199	-1.517682
C	-0.780530	-0.693833	1.595142
C	1.564369	-0.410278	0.966493
C	-2.135384	-0.537949	1.283743
C	-0.389484	-1.288553	2.857599
C	0.392287	1.210629	-2.800798
C	2.133391	0.544130	-1.184767
C	1.996763	-0.982369	2.226887
C	2.508149	0.027082	0.029201
C	1.358455	-2.024335	4.361409
C	-1.431907	1.786319	-4.345685
H	2.873394	0.887171	-1.893579
H	3.540751	-0.028330	0.309006
H	1.028579	-1.405156	5.200022
H	-3.536885	0.066478	-0.210261
H	-2.876125	-0.850704	2.007967
H	-0.551118	2.078178	-4.912203
H	-2.085874	2.648785	-4.194700
H	-1.992774	1.020743	-4.887481
H	2.441171	-2.120332	4.373829

H	0.887903	-3.005601	4.453763
N	0.979244	-1.409720	3.097078
N	-0.984183	1.267826	-3.061071
O	3.179910	-1.120176	2.557037
O	1.201248	1.604762	-3.650538
O	-1.191133	-1.693420	3.709335
O	-3.164287	0.908630	-2.550118

NDI$^{\bullet-}$-CBPQT^{0}

Atom	X	Y	Z
N	-4.542047	-2.665451	-0.627053
N	4.543278	2.669318	-0.613896
N	2.566325	-3.499114	-0.514264
N	-2.564714	3.497924	-0.488100
C	-5.814200	-1.940072	-0.637356
C	5.814681	1.942808	-0.630124
C	-3.858462	-2.891694	0.561615
C	3.805328	2.823078	-1.781968
C	-2.547182	-3.198955	0.609207
C	2.494964	3.133426	-1.791749
C	-1.699340	-3.237684	-0.585511
C	1.701363	3.243877	-0.565510
C	-2.494532	-3.120442	-1.810133
C	2.550925	3.199314	0.627842
C	-3.805328	-2.811797	-1.796934
C	3.861840	2.890829	0.576826
C	-0.326980	-3.322543	-0.560878
C	0.328956	3.326851	-0.538856
C	0.478346	-3.326163	0.663351
C	-0.513144	3.380880	-1.735572
C	1.826027	-3.392735	0.652286
C	-1.859241	3.462506	-1.678963

C	1.859447	-3.457350	-1.704133
C	-1.822714	3.388602	0.677182
C	0.513507	-3.373094	-1.758756
C	-0.474946	3.324012	0.686311
C	4.028419	-3.580563	-0.495634
C	-4.026723	3.581209	-0.467017
C	4.740118	-2.238452	-0.556523
C	-4.740031	2.240354	-0.536418
C	5.236210	-1.623257	0.595678
C	-4.837932	1.558019	-1.749768
C	5.683174	-0.303378	0.561981
C	-5.281938	0.242257	-1.792631
C	5.642016	0.432187	-0.625863
C	-5.642724	-0.429389	-0.623180
C	5.282916	-0.232452	-1.799881
C	-5.685488	0.298959	0.569121
C	4.839279	-1.548670	-1.765610
C	-5.238185	1.618543	0.611418
H	-6.390756	-2.267521	0.233766
H	6.363420	2.261719	-1.522802
H	-6.363364	-2.253544	-1.531699
H	6.392281	2.263858	0.242701
H	-4.464246	-2.823384	1.459778
H	4.369751	2.701375	-2.700711
H	-2.121934	-3.388285	1.588937
H	2.029289	3.261460	-2.762832
H	-2.030039	-3.243185	-2.782533
H	2.127366	3.384795	1.609055
H	-4.371044	-2.685854	-2.714304
H	4.468937	2.817825	1.473731
H	0.002231	-3.214445	1.630755
H	-0.066436	3.347730	-2.723107

H	2.407478	-3.353994	1.566727
H	-2.464627	3.502171	-2.577689
H	2.463876	-3.494798	-2.603636
H	-2.402948	3.345538	1.592186
H	0.065439	-3.334340	-2.745466
H	0.002580	3.209462	1.652706
H	4.330463	-4.204107	-1.343835
H	-4.313185	4.111101	0.446661
H	4.316791	-4.116457	0.413966
H	-4.329200	4.211033	-1.310409
H	5.210206	-2.154444	1.544178
H	-4.492832	2.029955	-2.664736
H	5.997225	0.179592	1.484411
H	-5.270522	-0.290792	-2.738739
H	5.272850	0.306313	-2.742802
H	-6.000756	-0.189534	1.488227
H	4.496010	-2.015491	-2.683933
H	-5.213101	2.144020	1.563111
C	-1.226095	0.045242	2.448135
C	0.002493	-0.005467	1.753525
C	-2.434097	0.074510	1.726366
C	-1.234037	0.045250	3.895174
C	-0.001081	-0.000213	0.325868
C	1.234338	-0.060585	2.443852
C	-1.224857	0.040153	-0.362533
C	1.218644	-0.035677	-0.371843
C	-2.423871	0.058690	0.350549
C	-1.243355	0.058416	-1.816765
C	1.258316	-0.073533	3.890514
C	2.437941	-0.082725	1.712637
C	1.238113	-0.042255	-1.826474
C	2.420805	-0.057830	0.337479

C	-0.046440	0.021903	-3.913408
C	-0.018937	-0.028829	5.989283
H	3.378377	-0.094999	2.252119
H	3.331090	-0.028535	-0.234264
H	-0.640319	-0.819292	-4.280110
H	-3.371432	0.084569	2.271725
H	-3.336328	0.033737	-0.217683
H	1.005705	-0.114106	6.342549
H	-0.614240	-0.872143	6.347468
H	-0.470903	0.892347	6.366321
H	0.975837	-0.053505	-4.275376
H	-0.505675	0.947567	-4.271786
N	-0.007402	0.010323	-2.461259
N	0.010424	-0.019440	4.533364
O	2.266045	-0.092870	-2.503987
O	2.299780	-0.126360	4.562631
O	-2.263708	0.115303	-2.505018
O	-2.261142	0.094581	4.588697

$BTA^{\bullet}$-$CBPQT^{4+}$

Atom	X	Y	Z
N	3.048027	3.527939	1.270297
N	-2.770761	-3.510630	-1.531633
N	-3.441437	2.873551	-1.299257
N	3.742441	-2.812222	0.978061
C	4.446556	3.364324	1.783446
C	-4.174945	-3.332581	-2.018332
C	2.018395	3.183605	2.065616
C	-1.740884	-3.364857	-2.387516
C	0.724749	3.180406	1.592658
C	-0.437458	-3.353414	-1.934284
C	0.461803	3.534524	0.266175

C	-0.169187	-3.502835	-0.569425
C	1.544243	3.948744	-0.517685
C	-1.256180	-3.743355	0.278456
C	2.822665	3.931434	0.004640
C	-2.540427	-3.729469	-0.222595
C	-0.911148	3.396185	-0.292129
C	1.208287	-3.341419	-0.026779
C	-2.035079	3.385138	0.540594
C	2.346378	-3.455574	-0.832395
C	-3.281006	3.112791	0.016852
C	3.595431	-3.181853	-0.309468
C	-2.389742	2.949890	-2.137717
C	2.670423	-2.745557	1.789196
C	-1.120284	3.205042	-1.662067
C	1.403260	-3.002958	1.315545
C	-4.774893	2.436493	-1.820344
C	5.081037	-2.389331	1.505100
C	-4.815846	0.927459	-1.921479
C	5.117999	-0.884967	1.655623
C	-5.091117	0.149595	-0.792370
C	5.559801	-0.075643	0.607154
C	-4.948181	-1.233945	-0.840426
C	5.407977	1.306231	0.673792
C	-4.528559	-1.860951	-2.017836
C	4.812576	1.896736	1.790222
C	-4.355353	-1.093753	-3.170860
C	4.470113	1.095468	2.881305
C	-4.498759	0.292308	-3.122881
C	4.622881	-0.284466	2.814886
H	4.469568	3.803518	2.781435
H	-4.228146	-3.768602	-3.015913
H	5.096071	3.951643	1.135239

H	-4.817588	-3.913195	-1.356358
H	2.254362	2.887870	3.078519
H	-1.986204	-3.229650	-3.432777
H	-0.050355	2.855003	2.274372
H	0.344022	-3.188681	-2.665471
H	1.422815	4.285090	-1.539209
H	-1.135539	-3.931933	1.336873
H	3.685618	4.223022	-0.579224
H	-3.405822	-3.874385	0.409774
H	-1.969829	3.578751	1.603161
H	2.292402	-3.752713	-1.871658
H	-4.167480	3.069831	0.635259
H	4.494298	-3.239058	-0.908908
H	-2.584321	2.774844	-3.187924
H	2.845437	-2.462765	2.817816
H	-0.308493	3.213658	-2.378262
H	0.583839	-2.895502	2.014240
H	-4.915648	2.918045	-2.788101
H	5.223999	-2.905060	2.455348
H	-5.525995	2.823661	-1.132467
H	5.828837	-2.752228	0.800816
H	-5.392768	0.620383	0.138578
H	5.990670	-0.521461	-0.284131
H	-5.139894	-1.817723	0.055074
H	5.724603	1.916845	-0.166366
H	-4.089966	-1.567558	-4.111045
H	4.071229	1.538172	3.788785
H	-4.344804	0.872679	-4.027669
H	4.342462	-0.886881	3.673773
S	-0.426473	-0.122448	2.888574
S	-2.127905	-0.258994	1.618346
N	1.142560	0.139642	-0.599560

N	0.727916	0.041435	1.692950
C	-1.599750	-0.181025	-1.108588
C	2.567556	0.308117	-0.380666
C	0.276530	0.017367	0.472651
C	-1.127512	-0.140444	0.194701
H	-2.654368	-0.308089	-1.294640
H	2.751475	0.318720	0.690439
H	2.906131	1.240968	-0.838586
S	-1.122462	-0.063520	-3.851953
S	0.912779	0.172644	-4.412582
N	1.559546	0.225302	-2.879579
C	-0.705334	-0.054611	-2.161298
C	0.699605	0.108813	-1.911661
H	3.113862	-0.510227	-0.856112

BTA$^{\bullet}$-CBPQT$^{\bullet 3+}$

Atom	X	Y	Z
N	2.899921	3.420471	1.437741
N	-2.749627	-3.464387	-1.193708
N	-3.768603	2.897909	-0.873145
N	3.711023	-2.913180	1.449354
C	4.293529	3.284432	1.910814
C	-4.148536	-3.316862	-1.704009
C	1.862817	3.251134	2.309171
C	-1.715929	-3.148722	-1.997018
C	0.567043	3.236119	1.893319
C	-0.421121	-3.154911	-1.522113
C	0.221562	3.381633	0.514010
C	-0.170632	-3.487572	-0.187193
C	1.338757	3.572097	-0.359490
C	-1.259299	-3.862320	0.606603
C	2.617889	3.585503	0.112237

C	-2.533945	-3.838038	0.082288
C	-1.125351	3.310194	0.050834
C	1.195517	-3.378574	0.387240
C	-2.234660	3.134049	0.936094
C	2.346428	-3.527265	-0.393076
C	-3.493343	2.921961	0.465584
C	3.588636	-3.284475	0.159920
C	-2.754693	3.152612	-1.753285
C	2.624225	-2.828772	2.238630
C	-1.476691	3.367888	-1.334525
C	1.362613	-3.057227	1.736507
C	-5.074981	2.406276	-1.353092
C	5.034753	-2.464671	1.991423
C	-5.038831	0.898157	-1.500205
C	5.053252	-0.954559	2.056850
C	-5.259084	0.074445	-0.391408
C	5.496486	-0.202492	0.967669
C	-5.024236	-1.294670	-0.468899
C	5.317553	1.178012	0.948397
C	-4.566003	-1.865043	-1.660022
C	4.685957	1.825154	2.011564
C	-4.434250	-1.059301	-2.791842
C	4.332626	1.082457	3.139952
C	-4.670013	0.312266	-2.712191
C	4.516384	-0.294738	3.164667
H	4.361481	3.783911	2.879503
H	-4.160862	-3.717685	-2.717986
H	4.933396	3.824465	1.211140
H	-4.781243	-3.947393	-1.079296
H	2.126975	3.130022	3.351730
H	-1.954881	-2.865177	-3.014324
H	-0.186262	3.090286	2.656220

H	0.369400	-2.857404	-2.200003
H	1.208723	3.711764	-1.424603
H	-1.139701	-4.177738	1.635127
H	3.468107	3.726390	-0.543429
H	-3.402119	-4.108638	0.667725
H	-2.110445	3.141666	2.010703
H	2.302153	-3.822309	-1.433850
H	-4.332956	2.758989	1.129243
H	4.500641	-3.361526	-0.416962
H	-3.021878	3.160858	-2.802643
H	2.784500	-2.545507	3.269449
H	-0.735488	3.550978	-2.101954
H	0.523847	-2.931438	2.409456
H	-5.288042	2.896341	-2.304531
H	5.151173	-2.928429	2.971808
H	-5.833047	2.721276	-0.633412
H	5.802600	-2.861597	1.328068
H	-5.592956	0.503449	0.548692
H	5.951864	-0.693670	0.112788
H	-5.182142	-1.910436	0.411714
H	5.636714	1.744226	0.078146
H	-4.129821	-1.494272	-3.739398
H	3.894832	1.572656	4.004140
H	-4.541444	0.925550	-3.599267
H	4.225113	-0.851145	4.050793
S	-0.555106	-0.246748	3.242778
S	-2.254053	-0.307647	1.967920
N	1.030730	0.057026	-0.230908
N	0.609581	-0.102931	2.056639
C	-1.732550	-0.000711	-0.741124
C	2.463571	0.113448	-0.010280
C	0.157410	-0.046033	0.837327

C	-1.252298	-0.107141	0.555874
H	-2.794673	-0.014786	-0.928820
H	2.646998	0.077106	1.060146
H	2.862811	1.037728	-0.429603
S	-1.253554	0.268053	-3.471166
S	0.790299	0.458828	-4.023013
N	1.447556	0.320407	-2.495398
C	-0.832571	0.140914	-1.784363
C	0.583125	0.181964	-1.533728
H	2.949207	-0.723404	-0.518638

BTA$^{\bullet}$-CBPQT$^{2(\bullet+)}$(T)

Atom	X	Y	Z
N	3.010678	3.526788	1.395739
N	-2.850168	-3.515170	-1.330418
N	-3.589171	2.847554	-1.064962
N	3.761235	-2.831880	1.098013
C	4.397325	3.365021	1.881944
C	-4.241034	-3.352713	-1.797335
C	1.963451	3.273232	2.234128
C	-1.804616	-3.398870	-2.201263
C	0.678959	3.225729	1.788099
C	-0.512132	-3.359099	-1.772604
C	0.360084	3.414953	0.408308
C	-0.187297	-3.406343	-0.380809
C	1.483190	3.711862	-0.426386
C	-1.310778	-3.578936	0.487019
C	2.749831	3.756517	0.075661
C	-2.582104	-3.620835	0.005335
C	-0.969014	3.283904	-0.090280
C	1.143081	-3.263848	0.111137
C	-2.099887	3.137255	0.772495

C	2.293320	-3.231154	-0.739127
C	-3.348273	2.920105	0.278147
C	3.543073	-3.019474	-0.236713
C	-2.547373	3.035604	-1.928010
C	2.695167	-2.891726	1.948666
C	-1.278835	3.262221	-1.486237
C	1.428828	-3.110825	1.502482
C	-4.908342	2.407359	-1.560594
C	5.078897	-2.390860	1.602787
C	-4.951567	0.898915	-1.681315
C	5.111057	-0.884559	1.756667
C	-5.231030	0.106352	-0.564244
C	5.539845	-0.068589	0.708589
C	-5.071710	-1.275094	-0.621500
C	5.377733	1.312339	0.776927
C	-4.629267	-1.889531	-1.796390
C	4.783767	1.901143	1.894100
C	-4.442310	-1.107636	-2.937272
C	4.442435	1.093022	2.980665
C	-4.603438	0.275504	-2.880346
C	4.605155	-0.286153	2.912520
H	4.449122	3.803390	2.880808
H	-4.309408	-3.784361	-2.797544
H	5.045988	3.948291	1.226019
H	-4.880872	-3.938841	-1.134796
H	2.212571	3.103459	3.273616
H	-2.062807	-3.323844	-3.250162
H	-0.083620	2.996208	2.520686
H	0.252886	-3.246902	-2.530521
H	1.364247	3.914735	-1.482769
H	-1.188204	-3.684162	1.556784
H	3.607820	3.970885	-0.549186

H	-3.438745	-3.741339	0.656172
H	-2.002226	3.196440	1.848390
H	2.210208	-3.379403	-1.808062
H	-4.207642	2.798660	0.924931
H	4.419068	-2.983828	-0.872447
H	-2.784404	2.979001	-2.982959
H	2.911631	-2.745082	2.998739
H	-0.511372	3.381722	-2.240616
H	0.644718	-3.122096	2.248007
H	-5.079345	2.889864	-2.524551
H	5.251203	-2.894621	2.556277
H	-5.661662	2.775827	-0.861316
H	5.833779	-2.739994	0.896427
H	-5.549154	0.568083	0.366121
H	5.969651	-0.512021	-0.185182
H	-5.267035	-1.870389	0.265999
H	5.682118	1.928549	-0.064646
H	-4.141399	-1.572550	-3.871842
H	4.025426	1.534937	3.880975
H	-4.428597	0.869840	-3.772800
H	4.315118	-0.897089	3.762654
S	-0.596213	-0.204618	3.139422
S	-2.269844	-0.333886	1.837662
N	1.036462	0.146259	-0.305076
N	0.582809	-0.015596	1.976535
C	-1.697390	-0.169834	-0.870285
C	2.457192	0.306076	-0.060529
C	0.152361	-0.008954	0.746794
C	-1.243204	-0.161876	0.440902
H	-2.748235	-0.290381	-1.078892
H	2.619197	0.357879	1.012188
H	2.808740	1.218409	-0.543507

S	-1.177240	-0.011183	-3.598645
S	0.870471	0.230741	-4.122086
N	1.498349	0.256542	-2.571771
C	-0.785174	-0.024810	-1.898741
C	0.619191	0.130913	-1.621138
H	3.000092	-0.538083	-0.487620

BTA$^{\bullet}$-CBPQT$^{2(\bullet+)}$(S)

Atom	X	Y	Z
N	-3.735697	3.180464	0.017831
N	3.418470	-3.185450	0.128116
N	3.339915	3.211328	0.255737
N	-3.658838	-3.220975	0.002650
C	-5.179232	2.865728	-0.033712
C	4.861199	-2.873782	0.173806
C	-3.017989	3.408242	-1.121093
C	2.701262	-3.299364	1.285778
C	-1.657381	3.502214	-1.108589
C	1.344145	-3.396193	1.282110
C	-0.909725	3.359128	0.102897
C	0.595065	-3.366545	0.064509
C	-1.708829	3.167621	1.272949
C	1.391162	-3.306925	-1.122032
C	-3.063659	3.073754	1.201940
C	2.748311	-3.205225	-1.061722
C	0.515111	3.370121	0.149293
C	-0.830716	-3.365748	0.042123
C	1.334288	3.390643	-1.022836
C	-1.612413	-3.190644	1.225443
C	2.691369	3.300538	-0.943462
C	-2.969826	-3.108617	1.175717
C	2.599830	3.240600	1.403361

C	-2.956712	-3.432292	-1.149364
C	1.242109	3.324365	1.380032
C	-1.595053	-3.505132	-1.159364
C	4.787920	2.926535	0.312286
C	-5.109149	-2.935005	-0.026847
C	5.036046	1.432998	0.298820
C	-5.355281	-1.441015	-0.033469
C	5.197299	0.756022	-0.911144
C	-5.418782	-0.732069	1.167497
C	5.215647	-0.636999	-0.945085
C	-5.438576	0.658987	1.165516
C	5.074923	-1.375940	0.230777
C	-5.392153	1.366437	-0.037334
C	5.015091	-0.697166	1.451052
C	-5.424843	0.656310	-1.238546
C	4.994923	0.694494	1.484814
C	-5.407678	-0.735116	-1.236550
H	-5.586205	3.333361	-0.931619
H	5.278393	-3.372373	1.050707
H	-5.649253	3.334570	0.833475
H	5.323143	-3.310318	-0.713682
H	-3.590180	3.502978	-2.035687
H	3.274257	-3.309239	2.204246
H	-1.170366	3.688875	-2.056962
H	0.858293	-3.497619	2.243747
H	-1.263521	3.051363	2.251960
H	0.940500	-3.293152	-2.106328
H	-3.669435	2.899824	2.081630
H	3.356963	-3.120006	-1.953115
H	0.902330	3.441488	-2.014234
H	-1.151696	-3.070170	2.197094
H	3.317389	3.281974	-1.826589

H	-3.563206	-2.939066	2.064753
H	3.154769	3.193114	2.331726
H	-3.542815	-3.533940	-2.054249
H	0.739601	3.352034	2.337879
H	-1.119536	-3.683899	-2.115105
H	5.179486	3.389962	1.219854
H	-5.520194	-3.414583	-0.916626
H	5.255720	3.416123	-0.543902
H	-5.556025	-3.410051	0.849025
H	5.278821	1.313320	-1.840145
H	-5.431808	-1.263398	2.115079
H	5.310878	-1.146709	-1.899600
H	-5.467284	1.192041	2.111726
H	4.952325	-1.253271	2.382238
H	-5.425583	1.188876	-2.185513
H	4.915149	1.202822	2.441471
H	-5.395590	-1.269876	-2.182248
S	-0.574780	-0.064933	3.387314
S	1.454615	-0.088949	2.756703
N	-0.916096	0.018523	-0.424326
N	-1.281463	-0.038010	1.877125
C	1.856576	-0.002867	0.013838
C	-2.339302	0.012145	-0.700757
C	-0.448770	-0.016811	0.875914
C	0.973786	-0.029156	1.082943
H	2.919692	-0.011451	0.189396
H	-2.876452	0.061819	0.242018
H	-2.602005	-0.897342	-1.242610
S	2.314245	0.085779	-2.729214
S	0.568952	0.088973	-3.943066
N	-0.562713	0.061013	-2.712552
C	1.354421	0.034366	-1.274544

C	-0.064569	0.038426	-1.510410
H	-2.589196	0.867363	-1.328998

BTA$^{\bullet}$-CBPQT$^{\bullet+}$

Atom	X	Y	Z
N	2.895989	3.503427	1.387486
N	-2.734872	-3.378396	-1.331576
N	-3.813766	3.068903	-1.065510
N	3.840132	-2.928381	1.223158
C	4.263532	3.314071	1.868163
C	-4.116697	-3.221164	-1.826968
C	1.821671	3.260358	2.230139
C	-1.670506	-3.249546	-2.176091
C	0.550505	3.178813	1.791904
C	-0.385975	-3.262816	-1.722229
C	0.192968	3.313052	0.380046
C	-0.090199	-3.384203	-0.328335
C	1.370037	3.505241	-0.469620
C	-1.236172	-3.539576	0.512979
C	2.620319	3.585532	0.031352
C	-2.497784	-3.530303	0.004687
C	-1.098353	3.266978	-0.086961
C	1.235275	-3.318006	0.190319
C	-2.269352	3.120132	0.776635
C	2.401827	-3.320406	-0.638075
C	-3.519771	3.005542	0.291160
C	3.644509	-3.121608	-0.114279
C	-2.735693	3.219902	-1.929268
C	2.760426	-2.994109	2.057073
C	-1.464131	3.344281	-1.501481
C	1.499813	-3.196244	1.588831
C	-5.072099	2.512668	-1.557436

C	5.134136	-2.435267	1.741217
C	-5.017093	1.002227	-1.684644
C	5.110628	-0.925782	1.858840
C	-5.258943	0.185779	-0.574974
C	5.528035	-0.121427	0.797380
C	-5.023994	-1.184643	-0.636043
C	5.316236	1.254379	0.827967
C	-4.541347	-1.769540	-1.809978
C	4.675224	1.853923	1.913504
C	-4.376383	-0.969842	-2.941645
C	4.335409	1.057579	3.009662
C	-4.611857	0.402160	-2.878539
C	4.553014	-0.315741	2.984998
H	4.332371	3.760700	2.864865
H	-4.154244	-3.635148	-2.836404
H	4.929333	3.880905	1.211370
H	-4.759981	-3.833559	-1.191651
H	2.070171	3.153337	3.280233
H	-1.906728	-3.121414	-3.225021
H	-0.212465	2.991430	2.538478
H	0.397183	-3.132255	-2.458532
H	1.264178	3.598981	-1.544283
H	-1.138574	-3.677775	1.581684
H	3.484372	3.732927	-0.608010
H	-3.371360	-3.645887	0.633425
H	-2.158886	3.097819	1.854404
H	2.334260	-3.475319	-1.707377
H	-4.378442	2.891244	0.944309
H	4.532720	-3.095989	-0.733561
H	-2.990152	3.254699	-2.983376
H	2.962047	-2.856997	3.111466
H	-0.703018	3.483305	-2.261270

H	0.703097	-3.206587	2.320605
H	-5.288903	2.972439	-2.525929
H	5.304973	-2.912735	2.708649
H	-5.864404	2.811903	-0.864724
H	5.912219	-2.774649	1.055289
H	-5.611818	0.626088	0.353557
H	5.990961	-0.573014	-0.076009
H	-5.199112	-1.795635	0.245342
H	5.619290	1.860418	-0.021986
H	-4.039564	-1.413213	-3.874673
H	3.878194	1.508033	3.886294
H	-4.452947	1.011777	-3.763943
H	4.264838	-0.915500	3.844150
S	-0.684324	-0.326671	3.162476
S	-2.345675	-0.255433	1.841489
N	1.018814	0.002630	-0.247733
N	0.523263	-0.197528	2.020720
C	-1.726415	0.015440	-0.846353
C	2.440382	0.106159	0.021078
C	0.110454	-0.098139	0.788359
C	-1.289189	-0.099938	0.464492
H	-2.780648	0.061160	-1.066153
H	2.605000	-0.044684	1.083596
H	2.790070	1.094421	-0.277664
S	-1.159669	0.207656	-3.562135
S	0.907933	0.303461	-4.055745
N	1.519736	0.193325	-2.499617
C	-0.789960	0.095693	-1.858881
C	0.618967	0.100409	-1.563414
H	2.975515	-0.647092	-0.556961

BTA$^{\bullet}$-CBPQT^{0}

Atom	X	Y	Z
N	2.701184	3.345711	1.396756
N	-2.578652	-3.279282	-1.072293
N	-4.027489	3.229262	-1.016633
N	4.133049	-3.281894	1.329900
C	4.064779	3.147351	1.882472
C	-3.954332	-3.122087	-1.532995
C	1.619603	3.247035	2.255403
C	-1.508410	-3.122264	-1.934797
C	0.341746	3.255056	1.826585
C	-0.225203	-3.178393	-1.522855
C	-0.012857	3.349792	0.410562
C	0.140143	-3.388054	-0.120832
C	1.169226	3.376158	-0.452655
C	-1.032270	-3.457990	0.751173
C	2.425141	3.371010	0.040256
C	-2.291595	-3.405175	0.275424
C	-1.305435	3.398558	-0.049777
C	1.430524	-3.478296	0.333876
C	-2.479327	3.356366	0.821785
C	2.613339	-3.426030	-0.529744
C	-3.732451	3.245482	0.342544
C	3.858376	-3.295667	-0.034266
C	-2.945792	3.330877	-1.886907
C	3.052129	-3.483196	2.184989
C	-1.672029	3.446053	-1.465442
C	1.786322	-3.611668	1.748566
C	-5.249595	2.565683	-1.469650
C	5.307263	-2.565736	1.823423
C	-5.090357	1.058929	-1.525889
C	5.100570	-1.063185	1.897072

C	-5.194573	0.288985	-0.362274
C	5.595180	-0.213680	0.906838
C	-4.869495	-1.063596	-0.373608
C	5.299747	1.148865	0.923662
C	-4.430230	-1.682562	-1.547691
C	4.494071	1.692441	1.924201
C	-4.393402	-0.931396	-2.722559
C	4.041155	0.850363	2.943155
C	-4.717170	0.424558	-2.711833
C	4.342024	-0.505967	2.931061
H	4.124330	3.590008	2.881530
H	-4.024964	-3.551653	-2.536699
H	4.736279	3.720789	1.236458
H	-4.592034	-3.725391	-0.879705
H	1.867314	3.175498	3.308710
H	-1.769896	-2.943015	-2.972171
H	-0.429242	3.177044	2.583876
H	0.540957	-3.026066	-2.274839
H	1.063567	3.397830	-1.531368
H	-0.916012	-3.553239	1.824009
H	3.294967	3.395843	-0.607841
H	-3.153658	-3.465346	0.930429
H	-2.367918	3.415855	1.897879
H	2.510454	-3.488730	-1.607951
H	-4.595010	3.202227	0.999921
H	4.730626	-3.224364	-0.677141
H	-3.202904	3.333113	-2.941289
H	3.309239	-3.565898	3.236569
H	-0.909760	3.555209	-2.228970
H	1.025669	-3.820430	2.492474
H	-5.503790	2.962282	-2.457319
H	5.545165	-2.966244	2.814528

H	-6.054299	2.846034	-0.783083
H	6.147998	-2.801872	1.163599
H	-5.511766	0.755846	0.566523
H	6.203934	-0.619759	0.102682
H	-4.939406	-1.636884	0.547117
H	5.681974	1.789537	0.132521
H	-4.087581	-1.401653	-3.653403
H	3.433365	1.253925	3.748742
H	-4.656799	0.996734	-3.634231
H	3.967416	-1.142204	3.728384
S	-0.387026	-0.367762	3.017587
S	-2.111679	0.041208	1.852146
N	1.087255	-0.027368	-0.495357
N	0.740105	-0.288312	1.794136
C	-1.685734	0.237466	-0.880565
C	2.529424	-0.045786	-0.327984
C	0.250497	-0.098407	0.603017
C	-1.162950	0.057086	0.390502
H	-2.743994	0.390628	-1.020480
H	2.749439	-0.167260	0.727583
H	2.942925	0.896445	-0.691677
S	-1.311742	0.400245	-3.631418
S	0.716463	0.366502	-4.277536
N	1.434279	0.166533	-2.773374
C	-0.823631	0.245084	-1.958905
C	0.599197	0.121771	-1.772984
H	2.959160	-0.875442	-0.888107
